# Acupuncture-related therapies for drug addiction: a systematic review and network meta-analysis

**DOI:** 10.3389/fnhum.2026.1800440

**Published:** 2026-05-20

**Authors:** HuiYan Zhao, Jung-Hee Jang, Yeon-Hee Ryu, Chang-Hyun Han

**Affiliations:** 1 KM Science Research Division, Korea Institute of Oriental Medicine, Daejeon, Republic of Korea; 2 Campus of Korea Institute of Oriental Medicine, Korean Convergence Medical Science Major, University of Science and Technology, Daejeon, Republic of Korea

**Keywords:** acupuncture-related therapy, drug addiction, network meta-analysis, systematic review, traditional medicine

## Abstract

**Introduction:**

Drug addiction is a major global health problem marked by compulsive drug-seeking and profound neuropsychiatric disturbances. This systematic review and network meta-analysis aimed to evaluate the effectiveness of various acupuncture-related therapies for managing drug addiction.

**Methods:**

Overall, 10 databases were searched for acupuncture-related therapies for drug addiction from inception to December 20, 2025. Methodological quality was assessed using Cochrane Handbook risk of bias 2.0. Pairwise meta-analyses were performed using RevMan 5.4 software and the network meta-analyses using R software.

**Results:**

A total of 35 randomized controlled trials encompassing 15 intervention types with 2,812 participants were included. The most frequently targeted acupoints were PC6, ST36, and SP6, while commonly used auricular points included TF4 (Shenmen), AH6a (Jiaogan), and CO14 (lung). Network meta-analysis indicated that acupuncture therapies, particularly when integrated with usual care, significantly improved drug addiction symptoms, although efficacy varied by symptom type. Treatment duration emerged as a potential moderating factor; specifically for withdrawal symptoms, long-term interventions (>20 days) demonstrated more consistent and certain effects compared to short-term treatments. While manual acupuncture (MA) and auricular acupuncture (AA) combined with usual care showed favorable trends for depression and anxiety, respectively, these rankings should be interpreted with caution due to the limited number of studies and observed heterogeneity.

**Discussion:**

Overall, the findings suggest that acupuncture is a promising adjunct therapy, with treatment duration playing a critical role in achieving stable clinical outcomes in addiction management.

**Systematic review registration:**

https://www.crd.york.ac.uk/PROSPERO/recorddashboard.

## 1 Introduction

Drug addiction is a chronic condition that affects an individual’s brain and behavior, and leads to cravings and the uncontrolled use of legal or illegal drugs (Nestler, 2004). This condition is rising with easier online access and several contributing factors, including expanded drug-supply networks, increased international travel and study, and the over-prescription of opioid painkillers such as fentanyl (Mi-kyung, 2023). Based on data from 73 countries in the World Health Organization (WHO) mortality databases between 1990 and 2021, drug use disorder mortality rates increased from 1.84 to 13.09 deaths per 1,000,000 people, and they are expected to significantly rise until 2040 (Kim et al., 2025). The most common treatment for drug addiction is behavioral interventions, including motivational interviewing, cognitive-behavioral therapy, contingency management, and 12-step programs (Volkow and Blanco, 2023). However, these interventions are limited by the associated economic burden and the need for sustained self-regulation (Acuff et al., 2020). Pharmacotherapy with methadone, buprenorphine, naltrexone, and lofexidine (Stotts et al., 2009) has demonstrated higher efficacy for symptoms including abstinence, long-term recovery, and prevention of relapse (Heidbreder, 2005). However, pharmacotherapy also leads to addiction and new substance use disorders, with consequent reduction in the medicine’s dose or duration of treatment (McElrath, 2018). Therefore, alternative treatment methods for drug addiction are needed.

Acupuncture therapies represent complementary and alternative treatments that have been widely used in 113 of 120 countries, based on WHO reports (Zhang et al., 2022). Internationally, the National Acupuncture Detoxification Association (NADA) provides a protocol for the use of auricular acupuncture (AA) in the treatment of trauma and substance misuse. Furthermore, numerous previous clinical and experimental studies have shown that acupuncture may be an effective adjunctive treatment for drug addiction withdrawal symptoms by suppressing the hyper-glutamatergic status and modulating the excitability of postsynaptic neurons in the dorsal CA1 (dCA1) (He et al., 2022; Ge et al., 2020). In addition, electroacupuncture (EA) can decrease the dopamine and tyrosine hydroxylase levels and increase the monoamine oxidase A levels to treat methamphetamine-administered mice (Ho et al., 2017).

To date, only one systematic review has assessed the effectiveness of acupuncture in the treatment of drug addiction (Ge et al., 2020). However, this review combined different methods of acupuncture, making it difficult to identify the most effective approach for drug addiction. Thus, in this review, we aimed not only to assess the effectiveness of combined methods of acupuncture but also to conduct subgroup analyses for each specific intervention. Because various acupuncture-related therapies involve drug addiction, we also conducted a network meta-analysis (NMA) to assess the ranking of interventions with direct and indirect comparisons among different interventions. The study can offer evidence supporting acupuncture as a treatment for drug addiction and help physicians to develop treatment strategies and governments to make decisions.

## 2 Methods

In this systematic review, we followed the Preferred Reporting Items for Systematic Reviews and Meta-Analyses (PRISMA) for Network Meta-Analyses (PRISMA-NMA) statement guidelines (Hutton et al., 2015). Moreover, the protocol for this study was registered in the international prospective review (PROPERO) under the registration number: CRD420251049076.

### 2.1 Databases and search strategy

Two authors (HYZ and JHJ) independently searched the databases using predefined search terms. We searched the following 10 databases: PubMed, EMBASE, Cochrane Central Register of Controlled Trials (CENTRAL), China Science and Technology Journal Database, SinoMed, Weipu, WanFang, Oriental Medicine Advanced Searching Integrated System, and KoreaMed, Korean Studies Information Service System for all-related studies from the inception of the study to December 20, 2025, without language limitations. To identify possible acupuncture therapies in drug addiction, we used a comprehensive search strategy based on the index of MeSH terms. The search strategies for each database are presented in Supplementary material 1.

### 2.2 Eligibility criteria

#### 2.2.1 Types of studies

We only included randomized controlled trials (RCTs). Animal studies, case studies, experimental studies (including observation studies or cross-sectional studies), and conference reports were excluded.

#### 2.2.2 Types of participants

Studies on people diagnosed with drug addiction or suffering from drug withdrawal syndrome were considered, irrespective of sex, race, or gender. Moreover, drug addiction was identified as the abuse of substances such as heroin, cocaine, morphine, pethidine, opioids, or methamphetamine (Hoots et al., 2018). Individuals with other medical conditions and those who used these drugs as anesthetics or analgesics were excluded.

#### 2.2.3 Types of interventions and comparisons

Studies that compared the use of manual acupuncture (MA), EA, warm acupuncture (WA), transcutaneous electric acupoint stimulation (TEAS), usual care (including relaxation or psychotherapy), no treatment, Western Medicine (WM) (including methadone, clonidine, opioid, buprenorphine, lofexidine tablets, buprenorphine, and palmitate long-acting injection), AA, and control placebo (including, sham with MA, sham with EA, sham with TEAS, or sham with AA) were included.

#### 2.2.4 Outcomes

The primary outcome was withdrawal symptoms based on the Clinical Institute Narcotic Assessment (Tompkins et al., 2009), Himmelsbach Withdrawal Symptom Rating Scale, Methamphetamine Withdrawal Questionnaire (Zhang, 1998), or Heroin Protracted Withdrawal Symptom Scale (Liu et al., 2000). Secondary outcomes included anxiety [Hamilton Anxiety Rating Scale (Clark and Donovan, 1994), Beck Anxiety Inventory (Fydrich et al., 1992), hospital anxiety scale (Turk et al., 2015), Visual Analogue Scale (Heller et al., 2016), or Self-rating anxiety scale (Dunstan and Scott, 2020)], depression [Hamilton Depression Rating Scale (Bagby et al., 2004), hospital depression scale (Turk et al., 2015), or self-rating depression scale (Biggs et al., 1978)], and adverse events.

### 2.3 Study selection and data extraction

Two reviewers (HYZ and JHJ) performed study selection and data extraction. Any disagreement about the process of study selection and data extraction was resolved by a third party (CHH). We reviewed the titles and abstracts of all screened studies and then examined the full texts to select the final studies. All studies were managed using the EndNote™ software (Clarivate Analytics). The included studies were extracted using a standardized Excel data sheet (Microsoft Office Professional Plus 2019). The following data were included: the first author, year of publication, condition of participants (including type of drug addiction and period of drug addiction), intervention (type of acupoint, treatment duration, and way of intervention), comparisons (treatment duration and way of comparisons), outcome measurements, and adverse events.

### 2.4 Quality assessment

Two researchers (HYZ and JHJ) independently evaluated the included studies by utilizing the Cochrane Handbook for risk of bias assessment tool 2.0 (Sterne et al., 2019), which encompassed five key domains: the randomization process, deviations from intended interventions, missing outcome data, outcome measurement, and selection of reported outcomes. Each item was assessed as being of “low,” “high,” or “some concerns.” Any discrepancies were resolved through the discussion with a third party (CHH). The figures illustrating the risk of bias were created using an Excel-based tool for implementing ROB2 (Creative Commons Attribution-Non Commercial-No Derivatives 4.0 International License).

### 2.5 Data synthesis

We classified the interventions into 10 categories based on similar methods, which included (1) WA, (2) Acupuncture therapies (MA or AA), (3) Electronic stimulation (TEAS or EA), (4) Usual care (relaxation or psychotherapy), (5) No treatment, (6) WM (including Methadone, Clonidine, Opioid, Buprenorphine, Lofexidine tablets, Buprenorphine, and palmitate long-acting injection), (7) placebo control (sham with MA, sham with EA, or sham with AA), (8) Acupuncture + WM (MA + WM or AA+WM), (9) Electronic stimulation + WM (TEAS+WM or EA + WM), and (10) Acupuncture + Usual care (MA + Usual care or AA + Usual care). We performed pair-wise meta-analysis and network meta-analysis based on these 10 criteria. To further evaluate the effectiveness of specific interventions, we conducted subgroup analyses for each intervention mentioned in the included studies.

#### 2.5.1 Pair-wise meta-analysis

Pair-wise meta-analysis was conducted on primary and secondary outcomes using Reviewer Manager Version 5.4 software (Cochrane, London, UK). In general, we used the mean difference with 95% confidence intervals (CIs). However, if the included studies used different questionnaires, we employed the standardized mean difference (SMD) with 95% confidence intervals (CIs). Furthermore, we used the I-squared statistic (*I*^2^) to assess the heterogeneity, with *I*^2^ ≥ 75% indicating considerable heterogeneity, and used the random-effects model; otherwise, a fixed-effects model was used.

#### 2.5.2 NMA

NMA was performed using the statistical software R (Version 4.4.2). Because of the different types of acupuncture therapies and acupoints included in the NMA, we used a random-effects model. Different questionnaires were used, and the SMD was calculated using the Netmeta package (Version 2.9–0). The Netsplit function was used to estimate the consistency test for a local approach, and the global approach was tested by using decomp.design methods. Finally, the surface under the cumulative ranking curve (SUCRA) was used to assess the treatment rank. In the NMA, the reference group was set up as WM. If there was no WM group among the comparison groups, no treatment was set up.

#### 2.5.3 Publication bias

If the RCTs included more than 10 studies, we used a funnel plot with Egger’s test to estimate the publication bias.

#### 2.5.4 Missing data

If the selected studies contained missing data, the required information was obtained by contacting the corresponding authors via phone or e-mail. If the corresponding author could not be reached, the study was excluded.

#### 2.5.5 Additional analysis

If a sufficient number of studies were available, sensitivity analyses of the primary and secondary outcomes were performed to test the robustness of the meta-analysis. In addition, we performed meta-regression analysis based on treatment duration. To explore the heterogeneity, we also performed subgroup analyses based on treatment duration and type of drug addiction.

## 3 Results

### 3.1 Characteristics of included studies

**Figure 1:**
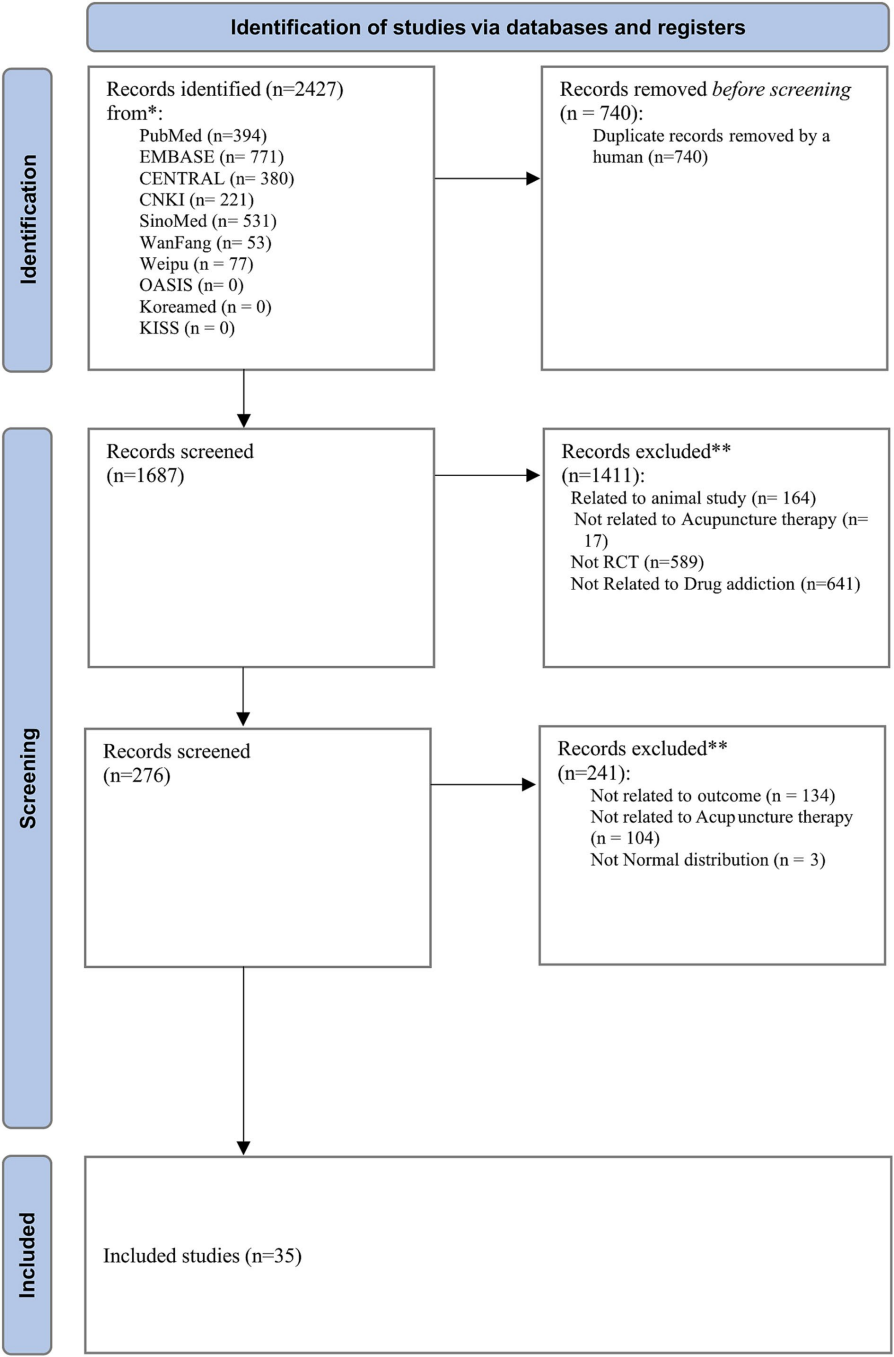
PRISMA flow diagram. CENTRAL, Cochrane Central Register of Controlled Trials; CNKI, China Science and Technology Journal Database; OASIS, Oriental Medicine Advanced Searching Integrated System; KISS, Korean Studies Information Service System.

A total of 2,427 studies were identified from 10 databases. After removing 740 duplicate records using EndNote software, 1,687 studies remained for screening. Based on title screening, 1,411 studies were excluded because they involved animal studies, were not related to acupuncture therapies, were not RCTs, or were not related to drug addiction. In the next step, full-text articles and abstracts were assessed for eligibility. A further 238 studies were excluded due to irrelevance to the outcomes of interest, inappropriate data distribution, or lack of relevance to acupuncture (which had not been identified during the initial title screening). A final total of 35 RCTs (Zhao et al., 1997; Zhang et al., 2000; Song et al., 2002; Shu et al., 2003; Zeng, 2003; Wu et al., 2003; Zhang et al., 2004; Rong, 2005; Zhang et al., 2005; Wen et al., 2005; Mu et al., 2005; Li et al., 2006a; Li et al., 2006b; Song et al., 2006; Riang et al., 2008; Mu et al., 2009; Li et al., 2012; Liang et al., 2014; Li et al., 2014; Rickard et al., 2015; Jackson et al., 2016; Luo, 2017; Wang, 2020; Gu et al., 2023; Kong, 2021; Gao et al., 2021; Sun et al., 2021; Yu et al., 2023; Chen et al., 2024; Wei et al., 2011; Zhu et al., 2008; Xu et al., 2016; Jing-ping et al., 2013; Chen, 2015; Liao, 2017), including 2,812 participants across 74 arms, were selected (Figure 1). Fourteen types of interventions were used: (1) MA, (2) EA,(3) WA, (4) TEAS, (5) Usual care, (6) No treatment, (7) WM, (8) AA,(9) placebo control, (10) MA + WM, (11) EA + WM, (12)TEAS+WM, (13) AA+Usual care, and (14) MA + Usual care.

The mean sample size in the included RCTs was 38.6, and participant ages ranged from 27 to 55 years. One study was conducted in the USA (Jackson et al., 2016), another one in Sweden (Rickard et al., 2015), and 33 in China (Zhang et al., 2000; Song et al., 2002; Shu et al., 2003; Zeng, 2003; Wu et al., 2003; Zhang et al., 2004; Rong, 2005; Zhang et al., 2005; Wen et al., 2005; Mu et al., 2005; Li et al., 2006a; Li et al., 2006b; Song et al., 2006; Riang et al., 2008; Mu et al., 2009; Li et al., 2012; Liang et al., 2014; Li et al., 2014; Luo, 2017; Wang, 2020; Gu et al., 2023; Kong, 2021; Gao et al., 2021; Sun et al., 2021; Yu et al., 2023; Chen et al., 2024; Wei et al., 2011; Zhu et al., 2008; Xu et al., 2016; Jing-ping et al., 2013; Chen, 2015; Liao, 2017; Zhao et al., 1997). Twenty-two studies were related to opioid or heroin (Zhang et al., 2000; Song et al., 2002; Shu et al., 2003; Zeng, 2003; Wu et al., 2003; Zhang et al., 2004; Rong, 2005; Zhang et al., 2005; Wen et al., 2005; Mu et al., 2005; Li et al., 2006a; Li et al., 2006b; Song et al., 2006; Riang et al., 2008; Mu et al., 2009; Li et al., 2012; Jackson et al., 2016; Wang, 2020; Gao et al., 2021; Wei et al., 2011; Zhu et al., 2008; Jing-ping et al., 2013); 11 to methamphetamine (Liang et al., 2014; Li et al., 2014; Luo, 2017; Gu et al., 2023; Kong, 2021; Sun et al., 2021; Yu et al., 2023; Chen et al., 2024; Xu et al., 2016; Chen, 2015; Liao, 2017); one to heroin, morphine, and pethidine (Zhao et al., 1997); and one to opioids, cannabinoids, sedatives, or hypnotics (Rickard et al., 2015). One study was a four-arm study (Wu et al., 2003), two were three-arm studies (Zhang et al., 2000; Wang, 2020), and the remaining 32 were two-arm studies. Meanwhile, 23 (63.7%) studies reported the withdrawal symptoms scale score (Zeng, 2003; Wu et al., 2003; Zhang et al., 2004; Rong, 2005; Zhang et al., 2005; Mu et al., 2005; Li et al., 2006a; Li et al., 2006b; Song et al., 2006; Liang et al., 2014; Jackson et al., 2016; Luo, 2017; Wang, 2020; Gu et al., 2023; Kong, 2021; Gao et al., 2021; Sun et al., 2021; Yu et al., 2023; Chen et al., 2024; Wei et al., 2011; Zhu et al., 2008; Xu et al., 2016; Zhao et al., 1997), 24 (68.6) the anxiety related score (Zhang et al., 2000; Song et al., 2002; Shu et al., 2003; Wen et al., 2005; Mu et al., 2005; Riang et al., 2008; Mu et al., 2009; Li et al., 2012; Liang et al., 2014; Li et al., 2014; Rickard et al., 2015; Jackson et al., 2016; Wang, 2020; Gu et al., 2023; Kong, 2021; Sun et al., 2021; Zhu et al., 2008; Xu et al., 2016; Jing-ping et al., 2013; Chen, 2015; Liao, 2017), and 13 (37.1%) the depression score (Shu et al., 2003; Mu et al., 2005; Mu et al., 2009; Li et al., 2012; Liang et al., 2014; Jackson et al., 2016; Wang, 2020; Gu et al., 2023; Kong, 2021; Zhu et al., 2008; Xu et al., 2016; Chen, 2015; Liao, 2017). The characteristics of each study are shown in Table 1.

**Table 1:** Characteristics of included studies.

No	First author (Year) Country	Type of drug	Drug period time	Mean age (range)	Intervention (Way of treatment) (Sample size)	Acupoint	Control (Way of treatment) (Sample size)	Treatment duration	Outcome measure	Effect estimate
1	Zhao et al. (1997) China	Heroin, Morphine, Phetidine	Total: 12.46 ± 18.64 months	Total: 28.60 ± 6.96	(A) EA: 12–20 mA, Every 2–3 times, 30 min, 10 days. (54)	SJ5, PC6, LI4, PC8	(B) WM: Clonidine, 0.075 mg or 0.1 mg for 3 days; from the 4th day, reduce the number of medicines; three times a day, 4th day, 1/4–1/6, 10 days (56)	10 days	Withdrawal symptoms (CINA)	MD, −0.36 [−2.32, 1.60], *p* = 0.72
2.	Zhang et al. (2000) China	Heroin	(A) 3.2 ± 0.2 years (B) 3.4 ± 0.3 years	(A) 27.1 ± 0.7 (B) 25.4 ± 1.2	(A) TEAS: 2/100HZ, 6 s for one period, 4 times a day, from the 4th day, 2 times a day, from the 5th day, once a day (121)	SJ5, PC6, LI4, PC8, ST36, SP6	(B) Sham_TEAS (Same way) as (A), but no electronic stimulation (60)	15 days	Anxiety (VAS)	MD, −3.49 [−4.19, −2.79], *p* < 0.00001
3	Song et al. (2002) China	Heroin	(A) 32.61 ± 18.12 months (B) 40.56 ± 16.08 months	(A) 27.38 ± 5.95 (B) 27.57 ± 4.55	(A) MA + WM: 0.5–0.1cun; once a day, 60 min plus (B) (30)	PC6, LI4, SP6	(B) WM: (Methadone, reduce the dosage) (30)	7 days	Anxiety (HAMA)	MD, −2.32 [−3.35, −1.29], *p* < 0.00001
4	Shu et al. (2003) China	Heroin	(A) 6.73 ± 2.64 years (B) 7.92 ± 1.51 years	(A) 33.60 ± 5.20 (B) 30.92 ± 5.74	(A) TEAS: 2.5HZ, once a day, 30 min for one time. (78)	PC6, SP6, GV4	(B) No treatment (12)	5 days	(1) Anxiety (SAS) (2) Depression (SDS)	(1) MD, −5.31 [−16.87, 6.25], *p* = 0.37 (2) MD, −7.48 [−17.35, 2.39], *p* = 0.14
5	Zeng (2003) China	Heroin	(A) 6.00 ± 2.82 years (B) 6.23 ± 2.93 years	(A) 33.16 ± 5.51 (B) 34.23 ± 4.83	(A) EA + WM: 30 min plus (B) (31)	GV4, GV20, DU4, DU11, GV10, GV9	(B) WM: methadone, reduce the daily dose by about 20%, once a day (26)	10 days	Withdrawal symptoms (Heroin Protracted Withdrawal Symptom Scale)	MD, −2.69 [−4.88, −0.50], *p* = 0.02
6	Wu et al. (2003) China	Heroin	n.r.	n.r.	(A) MA: 30 min, 2 times a day for first 3 days, once a day, 7 days (30) (B) WM: Opioid, Buprenorphine, and reduce the doses, 3 times a day/for the first 3 days with 100 mg; 2 times a day/for 4–6 days with 50 mg; 2 times a day/for 7–10 days with 50 mg (30)	PC6, LI4, ST36, SP6	(C) MA + WM: (A) plus (B) (30) (D) TEAS plus WM: 3 times a day for first 3 days, 3 times a day, 2 times for 7 days plus (B) (30)	10 days	Withdrawal symptoms (Heroin Protracted Withdrawal Symptom Scale)	(A) vs. (B) MD, −16.27 [−20.79, −11.75], *p* < 0.00001 (C) vs. (A) MD, 1.60 [0.28, 2.92], *p* = 0.02 (A) vs. (D) MD, −12.04 [−17.63, −6.45], *p* < 0.00001 (B) vs. (C) MD, −14.67 [−19.30, −10.04], *p* < 0.00001 (B) vs. (D) MD, −4.23 [−11.36, 2.90], *p* = 0.25 (C) vs. (D) MD, −10.44 [−16.11, −4.77], *p* = 0.0003
7	Zhang et al. (2004) China	Heroin	(A) 8.2 years (B) 7.96 years	(A) 26.52 (B) 28.11	(A) EA + WM: 1–1.2 cun, 2 times for every day, 30 min plus (B) (43)	GV20, GV19, EX-HN5, LI11, PC6, ST36, GB34, HT7	(B) WM: methadone, reduce the daily dose (43)	10 days	Withdrawal symptoms (Himmelsbach Withdrawal Symptom Rating Scale)	MD, 0.02 [−0.01, 0.05], *p* = 0.15
8	Rong (2005) China	Heroin	(A) 7.10 ± 3.28 years (B) 7.10 ± 4.09 years	(A) 31.87 ± 5.30 (B) 29.23 ± 6.98	(A) MA: 1.0–2.0cun, Once a day, 20 min (31)	EX-HN1, PC6, LI4, ST36, SP6	(B) WM: methadone, reduce the daily dose (30)	10 days	Withdrawal symptoms (Himmelsbach Withdrawal Symptom Rating Scale)	MD, −14.88 [−19.30, −10.46], *p* < 0.00001
9	Zhang et al. (2005) China	Heroin	(A) 8.2 years (B) 8.0 years	(A) 26.5 (B) 28.1	(A) EA + WM: 1.2–1.5 cun, twice every day, 30 min plus (B) (43)	GV20, GV19, EX-HN5, LI11, PC6, ST36, GB34, HT7	(B) WM: Initial dose was used for 3 days. If the initial dose was less than 10 mg, 3 days later, 5 mg was reduced every second day. If the initial dose was more than 10 mg, 3 days later, the dose was reduced by 10 mg every day (43)	10 days	Withdrawal symptoms (Himmelsbach Withdrawal Symptom Rating Scale)	MD, 0.10 [0.07, 0.13], *p* < 0.00001
10	Wen et al. (2005) China	Heroin	(A) 20.08 ± 8.26 months (B) 20.19 ± 8.44 months	(A) 33.85 ± 6.39 (B) 33.75 ± 7.5	(A) MA: 30 min, once a day (111)	SJ5, PC6, LI4, ST36	(B) WM: Lofexidine tablets, 0.1 mg, 2times a day (109)	10 days	(1) Anxiety (HAMA) (2) Withdrawal symptoms (Heroin Protracted Withdrawal Symptom Scale)	(1) MD, −3.64 [−6.99, −0.29], *p* = 0.03 (2) MD, −1.49 [−4.54, 1.56], *p* = 0.34
11	Mu et al. (2005) China	Heroin	(A) 4.9 ± 3.69 years (B) 4.6 ± 3.24 years	(A) 28.56 ± 8.56 (B) 31.68 ± 10.74	(A) Sham_EA: 20 min with no electronic stimulation (30)	ST36, SP6	(B) No treatment (30)	10 weeks	(1) Withdrawal symptoms (Heroin Protracted Withdrawal Symptom Scale) (2) Anxiety (HAMA) (3) Depression (SDS)	(1) MD, −1.96 [−3.28, −0.64], *p* = 0.004 (2) MD, −3.88 [−5.63, −2.13], *p* < 0.0001 (3) MD, −2.40 [−4.03, −0.77], *p* = 0.004
12	Li et al. (2006a) China	Heroin	Total: 6–120 months	Total: 28.25 ± 5.45	(A) AA + Usual care: press for 3 times a day, 30–60s, change the pill, every 3 days plus (B) (45)	CO17, TF1, TF2, CO18, CO7	(B) Usual care: psychotherapy (45)	3 months	Withdrawal symptoms (Heroin Protracted Withdrawal Symptom Scale)	MD, −6.36 [−6.84, −5.88], *p* < 0.00001
13	Li et al. (2006b) China	Heroin	(A) 5.00 ± 2.82 years (B) 5.14 ± 2.93 years	(A) 30.16 ± 5.51 (B) 28.45 ± 6.95	(A) MA + WM: 30 min, 2 times a day (31)	GV20, GV19, EX-HN5, LI11, PC6, ST36, GB34, HT7	(B) WM: Buprenorphine, Reduce the dosage (33)	10 days	Withdrawal symptoms (Heroin Protracted Withdrawal Symptom Scale)	MD, 0.00 [−1.71, 1.71], *p* = 1.00
14	Song et al. (2006) China	Heroin	(A) 78.71 ± 41.09 months (B) 73.81 ± 29.71 months	n.r.	(A) AA: press 2–3 times a day, 1–3 min (70)	TF1, CO18, CO15, CO10, AT4	(B) No treatment (80)	20 days	Withdrawal symptoms (Heroin Protracted Withdrawal Symptom Scale)	MD, −2.06 [−2.75, −1.37], *p* < 0.00001
15	Riang et al. (2008) China	Heroin	Total: 64.94 ± 52.73 months	Total: 34.28 ± 9.24	(A) MA (30)	PC6, HT7, ST36, SP 6, EX-B2, BL23	(B) No treatment (30)	18 days	Anxiety (HAMA)	MD, −9.40 [−10.96, −7.84], *p* < 0.00001
16	Mu et al. (2009) China	Heroin	(A) 4.79 ± 3.52 years (B) 4.84 ± 3.15 years	(A) 28.65 ± 8.87 (B) 30.23 ± 9.74	(A) Sham_EA: 20 min with no electronic stimulation (30)	ST36, SP6	(B) No treatment (30)	10 weeks	(1) Anxiety (SAS) (2) Depression (SDS)	(1) MD, −0.03 [−0.38, 0.32], *p* = 0.87 (2) MD, −1.09 [−2.72, 0.54], *p* = 0.19
17	Li et al. (2012) China	Heroin	(A) 11.7 ± 7.7 years (B) 9.4 ± 8.1 years	(A) 36.3 ± 7.4 (B) 38.1 ± 8.2	(A) MA: 30 min, Once a day, 5 times a week (48)	PC6, LI4, ST36, SP6	(B) sham_MA: not acupoint (41)	8 weeks	(1) Anxiety (SAS) (2) Depression (SDS)	(1) MD, −7.80 [−11.49, −4.11], *p* < 0.0001 (2) MD, −4.80 [−7.59, −2.01], *p* = 0.0007
18	Liang et al. (2014) China	Methamphetamine	(A) 48.2 ± 31.7 months (B) 57.4 ± 27.9 months (C) 51.4 ± 30.0 months	(A) 37.0 ± 8.4 (B) 39.9 ± 8.0 (C) 38.5 ± 7.3	(A) EA: 120 min (26) (B) AA: 20 min (26)	EA: PC6, HT7, ST36, SP6 AA: AH6a, TF4, CO14, CO12	(C) No treatment (25)	4 weeks	(1) Anxiety (HAMA) (2) Depression (HAMD) (3) Withdrawal symptoms (Methamphetamine Withdrawal Questionnaire)	(A) vs. (B) (1) MD, −2.11 [−3.74, −0.48], *p* = 0.11 (2) MD, −4.39 [−6.11, −2.67], *p* < 0.00001 (3) MD, −2.04 [−3.58, −0.50], *p* = 0.009 (A) vs. (C) (1) MD, −6.06 [−7.88, −4.24], *p* < 0.00001 (2) MD, −7.93 [−10.04, −5.82], *p* < 0.00001 (3) MD, −3.83 [−5.42, −2.24], *p* < 0.00001 (B) vs. (C) (1) MD, −6.06 [−7.88, −4.24], *p* < 0.00001 (2) MD, −7.93 [−10.04, −5.82], *p* < 0.00001 (3) MD, −3.83 [−5.42, −2.24], *p* < 0.00001
19	Li et al. (2014) China	Methamphetamine	(A) 2.3 years (B) 2.2 years	(A) 36 (B) 36	(A) MA: 5 times a week (45)	PC6, ST36, GV20, HT7	(B) No treatment (45)	3 weeks	(1) Anxiety (HAMA) (2) Depression (HAMD)	(1) MD, −20.78 [−22.74, −18.82], *p* < 0.00001 (2) MD, −12.74 [−13.66, −11.82], *p* < 0.00001
20	Chen (2015) China	Methamphetamine	(A) 67.52 ± 18.15 months (B) 63.74 ± 20.11 months	(A) 30.23 ± 6.46 (B) 29.70 ± 6.15	(A) MA + Usual care: 30 min, 2 times a week plus (B) (30)	PC6, SP6, GV20, HT7	(B) Usual care: psychotherapy (30)	2 months	(1) Anxiety (SAS) (2) Depression (SDS)	(1) MD, −2.10 [−6.84, 2.64], *p* = 0.39 (2) MD, −1.30 [−7.35, 4.75], *p* = 0.67
21	Rickard et al. (2015) Sweden	opioids, cannabinoids, sedatives, or hypnotics	n.r.	(A) 44.1 ± 14.0 (B) 44.8 ± 13.6	(A) AA: each week during another 2 weeks; each week for the following 2 weeks (37)	TF1, CO10, AH6a, CO14, CO12	(B) Usual care: relaxation (36)	2 weeks	Anxiety (ISI)	MD, 3.30 [−2.06, 8.66], *p* = 0.23
22	Jackson et al. (2016) USA	opioids	n.r.	Total: 56.5 ± 17.3	(A) AA: 35 min, once per month (9)	TF1, CO10, AH6a, CO14, CO12	(B) Usual care: psychotherapy (6)	2 weeks	(1) Anxiety (HADS-A) (2) Depression (HADS-D) (3) Withdrawal symptoms (CINA)	(1) MD, 0.80 [−2.30, 3.90], *p* = 061 (2) MD, −1.90 [−4.77, 0.97], *p* = 0.19 (3) MD, −0.70 [−4.54, 3.14], *p* = 0.72
23	Liao (2017) China	Methamphetamine	(A) 7.1 ± 3.889 years (B) 6.57 ± 2.75 years	(A) 35.23 ± 7.798 (B) 33.30 ± 7.512	(A) TEAS: 60 min, treat 5 days and rest 2 days (30)	BL23, BL15, BL18, BL20	(B) Sham_TEAS: 60 min, treat 5 days and rest 2 days; no stimulation (30)	28 days	(1) Anxiety (SAS) (2) Depression (SDS)	(1) MD, −5.06 [−8.60, −1.52], *p* = 0.005 (2) MD, −4.14 [−8.96, 0.68], *p* = 0.09
24	Luo (2017) China	Methamphetamine	(A) 7.5 (B) 7.0	(A) 31.3 (B) 29.2	(A) TEAS: 60 min, treat 5 days (28)	ST36, SP6, PC6, HT7	(B) Sham_TEAS: 60 min, treat 5 days and rest 2 days; no stimulation (30)	4 weeks	Withdrawal symptoms (Methamphetamine Withdrawal Questionnaire)	MD, −1.56 [−2.03, −1.09], *p* < 0.00001
25	Wang (2020) China	Opioid	(A) 19.10 ± 5.88 years (B) 17.59 ± 7.93 years (C) 16.90 ± 7.97 years	(A) 46.00 ± 6.37 (B) 43.86 ± 8.44 (C) 43.00 ± 6.16	(A) EA: 2/100HZ, 10-15 mA (20)	ST36, SP6, PC6, HT7	(B) Sham_EA: not an acupoint (21) (C) No treatment (20)	4 weeks	(1) Anxiety (HAMA) (2) Depression (HAMD) (3) Withdrawal symptoms (The clinical opiate withdrawal scale)	(A) vs. (B) (1) MD, −0.19 [−2.34, 1.96], *p* = 0.86 (2) MD, −1.09 [−3.46, 1.28], *p* = 0.37 (3) MD, −0.61 [−2.13, 0.91], *p* = 0.43 (B) vs. (C) (1) MD, −0.38 [−3.14, 2.38], *p* = 0.79 (2) MD, −1.00 [−3.25, 1.25], *p* = 0.38 (3) MD, −1.58 [−3.40, 0.24], *p* = 0.09 (A) vs. (C) (1) MD, −0.57 [−3.06, 1.92], *p* = 0.65 (2) MD, −2.09 [−4.26, 0.08], *p* = 0.06 (3) MD, −2.19 [−3.92, −0.46], *p* = 0.01
26	Gu et al. (2023) China	Methamphetamine	(A) 8 (6,15) months (B) 8 (6,30) months	(A) 45 ± 10 (B) 43 ± 11	(A) EA: 2HZ, 20 min, every day for 3 days, rest 2 days, and 3 times after days. (39)	PC6, HT7, ST36, SP6, EX-B2, EX-HN5, BL23, GV29	(B) Usual care: psychotherapy (39)	10 days	(1) Anxiety (HAMA) (2) Depression (HAMD) (3) Withdrawal symptoms (Methamphetamine Withdrawal Questionnaire)	(1) MD, −3.64 [−5.12, −2.16], *p* < 0.00001 (2) MD, −4.95 [−7.02, −2.88], *p* < 0.00001 (3) MD, −4.05 [−5.41, −2.69], *p* < 0.00001
27	Kong (2021) China	Methamphetamine	(A) 85 (8, 34) months (B) 83 (22, 170) months	(A) 46.03 ± 10.36 (B) 43.96 ± 9.34	(A) EA: 20 Hz, 20 min, every day for 3 days, rest 2 days, and 3 times after days (35)	PC6, HT7, ST36, SP6, EX-B2, EX-HN5, BL23, GV29	(B) Usual care: psychotherapy (35)	3 weeks	(1) Anxiety (HAMA) (2) Depression (HAMD) (3) Withdrawal symptoms (Methamphetamine Withdrawal Questionnaire)	(1) MD, −1.62 [−3.03, −0.21], *p* = 0.02 (2) MD, −3.20 [−5.44, −0.96], *p* = 0.005 (3) MD, −5.11 [−6.45, −3.77], *p* < 0.00001
28	Gao et al. (2021) China	Heroin	(A) 189.3 ± 70.5 months (B) 181.8 ± 72.6 months	(A) 47 ± 8 (B) 43 ± 9	(A) WA: 20 min, once every 2 days (39)	EX-B2, BL23, PC6, HT7, ST36, SP6	(B) Usual care: psychotherapy (39)	20 days	Withdrawal symptoms (Heroin Protracted Withdrawal Symptom Scale)	MD, −5.85 [−8.81, −2.89], *p* = 0.0001
29	Sun et al. (2021) China	Methamphetamine	(A) 3.44 ± 0.84 years (B) 3.29 ± 1.04 years	(A) 33.32 ± 7.29 (B) 32.63 ± 6.03	(A) AA+Usual care: 3–5 times/a day; 3–5 min/one time plus (B) (32)	CO18, CO15, AT4, AH6a, TF4	(B) Usual care: psychotherapy (32)	20 days	(1) Anxiety (HAMA) (2) Withdrawal symptoms (Quality of life for drug addicts)	(1) MD, −4.87 [−6.86, −2.88], *p* < 0.00001 (2) MD, 1.13 [0.08, 2.18], *p* = 0.04
30	Yu et al. (2023) China	Methamphetamine	(A) 115.45 ± 87.32 months (B) 114.12 ± 103.94 months	(A) 44.07 ± 9.35 (B) 42.51 ± 11.40	(A) EA: 20 min, 3 times a week (44)	EX-B2, BL23, PC6, HT7, ST36, SP6	(B) Usual care: psychotherapy (43)	20 days	Withdrawal symptoms (Methamphetamine Withdrawal Questionnaire)	MD, −4.99 [−6.01, −3.97], *p* < 0.00001
31	Chen et al. (2024) China	Methamphetamine	n.r.	(A) 35 (24–46) (B) 36.13 (25.68–46.58)	(A) EA + WM: 0.5–0.8cun, 2 Hz, 20 min, once every other day for 3 months plus (B) (55)	PC6, ST36, SP6, HT7, EX-B2	(B) WM: palmitate long-acting injection, 100 mg was injected (54)	3 months	Withdrawal symptoms (Methamphetamine Withdrawal Questionnaire)	MD, −6.95 [−7.80, −6.10], *p* < 0.00001
32	Jing-ping et al. (2013) China	Heroin	(A) 2.45 ± 1.62 (B) 2.54 ± 1.34	(A) 32.51 ± 6.35 (B) 33.43 ± 5.77	(A) EA: 5HZ, 20 min, once a day (30)	EX-B2, BL23	(B) WM: Methadone and Doxepin; 10 mg of Methadone for each dose, 3 doses a day, and 25 mg of Doxepin for each dose, 3 doses a day (30)	2 weeks	Anxiety (SAS)	MD, −4.58 [−7.76, −1.40], *p* = 0.005
33	Wei et al. (2011) China	Heroin	(A) 10.10 ± 6.27 years (B) 9.70 ± 5.69 years	(A) 35.27 ± 6.45 (B) 35.87 ± 7.71	(A) AA: 3–5 times/a day; change every 2 days (30)	CO17, CO10, AT4, AH6a, TF4, CO14	(B) Sham_AA: 3–5 times/a day; change every 2 days, no stimulation (30)	15 days	Withdrawal symptoms (Heroin Protracted Withdrawal Symptom Scale)	MD, 0.84 [−2.30, 3.98], *p* = 0.60
34	Zhu et al. (2008) China	Heroin	(A) 3.73 ± 2.61 years (B) 4.08 ± 3.74	(A) 31.7 ± 7.32 (B) 30.6 ± 8.39	(A) TEAS: 100HZ, 3 times a week (30)	SJ5, PC6, LI4, PC8	(B) Sham_TEAS: 100HZ, 3 times a week (30)	12 weeks	(1) Anxiety (HAMA) (2) Depression (HAMD) (3) Withdrawal symptoms (Heroin Protracted Withdrawal Symptom Scale)	(1) MD, −14.10 [−15.73, −12.47], *p* < 0.00001 (2) MD, −20.70 [−22.65, −18.75], *p* < 0.00001 (3) MD, −10.10 [−11.72, −8.48], *p* < 0.00001
35	Xu et al. (2016) China	Methamphetamine	Total: 51.90 ± 28.15 months	n.r.	(A) AA: 25 min, 3 times a week (26)	AH6a, TF4, CO14, CO12	(B) No treatment (25)	28 days	(1) Anxiety (HAMA) (2) Depression (HAMD) (3) Withdrawal symptoms (Methamphetamine Withdrawal Questionnaire)	(1) MD, −7.80 [−9.54, −6.06], *p* < 0.00001 (2) MD, −12.22 [−14.15, −10.29], *p* < 0.00001 (3) MD, −5.78 [−7.27, −4.29], *p* < 0.00001

AA, auricular acupuncture; MA, Manual acupuncture; EA, electroacupuncture; TEAS, transcutaneous electric acupoint stimulation; WM, Western medicine; HAMA, Hamilton Anxiety Rating Scale; SAS, Self-rating anxiety scale; HADA, Hamilton Depression Rating Scale; SDS, self-rating depression scale; WA, Warm acupuncture; MD, Mean difference; CINA, clinical institute narcotic assessment; VAS, Visual Analogue Scale.

### 3.2 Risk of bias

**Figure 2:**
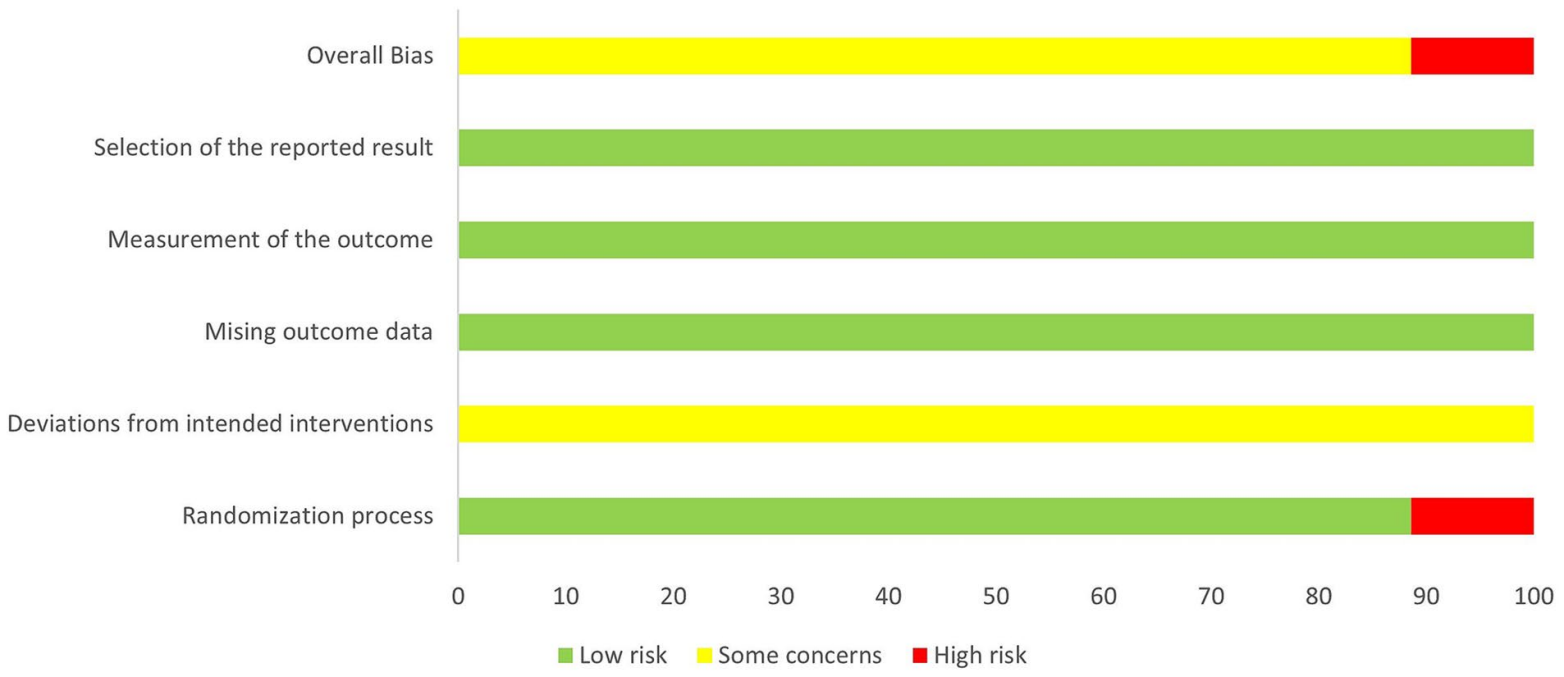
Summary of risk for bias.

The risk of bias in the included studies is presented in Figure 2. Regarding the randomization methods, four studies (Zeng, 2003; Wu et al., 2003; Rong, 2005; Chen, 2015) used visiting sequence methods, which resulted in a high risk of bias, while the remaining 31 studies utilized appropriate randomization methods. Because of the inherent nature of acupuncture, all included studies were rated as having some concerns regarding deviations from the intended interventions. No issues with missing outcome data, measurement of outcomes, or selection of reported results were observed, which was considered to indicate low risk of bias. Overall, the low risk of bias was 88.6% and the high risk of bias 11.4%.

### 3.3 Distribution of acupoints

**Figure 3:**
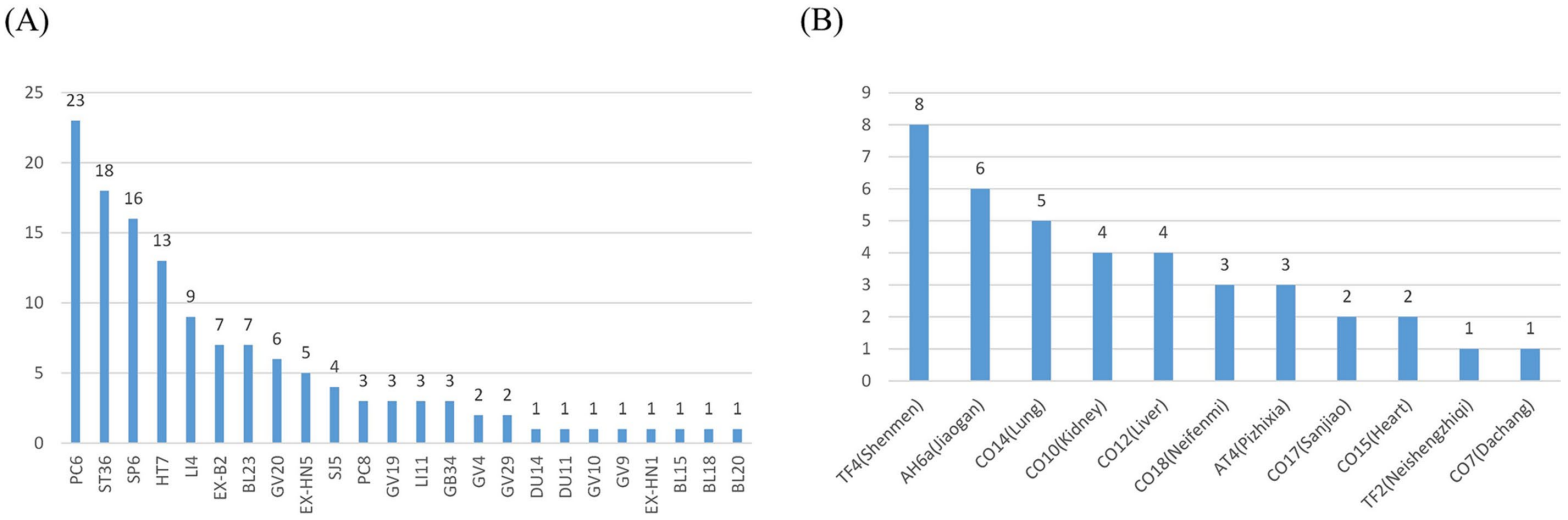
Frequency of acupoint. **(A)** Acupoint of acupuncture. **(B)** Acupoint of auricular acupuncture.

The three most frequent acupoints were PC6 (23), ST36 (18), and SP6 (13) in the MA, EA, WA, and TEAS. Moreover, the most frequent top 3 AA acupoints were TF4 (Shenmen) (8), AH6a (Jiaogan) (6), and CO14 (lung) (5). The distribution of acupoints is shown in Figure 3.

### 3.4 Comparative effectiveness

#### 3.4.1 Withdrawal symptoms (primary outcome)

##### 3.4.1.1 Pairwise meta-analysis

Pairwise meta-analysis revealed statistically significant differences across the following comparisons. (1) Acupuncture + WM vs. Acupuncture (MD, 1.60 [0.28, 2.92], *p* = 0.02); (2) Acupuncture vs. No treatment (SMD, −1.38 [−2.05, −0.71], *p* < 0.0001, *I*^2^ = 79%); (3) Electronic stimulation + WM vs. Acupuncture (MD, 12.04 [6.45, 17.63], *p* < 0.0001); (4) Electronic stimulation + WM vs. Acupuncture + WM (MD, 10.44 [4.77, 16.11], *p* = 0.0003), (5) Electronic stimulation vs. No treatment (SMD, −1.52 [−3.00, −0.03], *p* = 0.05, *I*^2^ = 89%); (6) Electronic stimulation vs. Placebo (SMD, −1.67 [−3.22, −0.11], *p* = 0.04, *I*^2^ = 94%); (7) Electronic stimulation vs. Usual care (SMD, −1.70 [−2.13, −1.26], *p* < 0.00001, *I*^2^ = 52%); (8) Electronic stimulation vs. Acupuncture (MD, −2.04 [−3.58, −0.50], *p* = 0.009); (9) Placebo vs. No treatment (SMD, −0.65 [−1.05, −0.25], *p* = 0.001, *I*^2^ = 0%); and (10) Warm acupuncture vs. Usual care (MD, −5.85 [−8.81, −2.89], *p* = 0.0001) (Supplementary material 2).

##### 3.4.1.2 NMA for withdrawal symptoms

**Figure 4:**
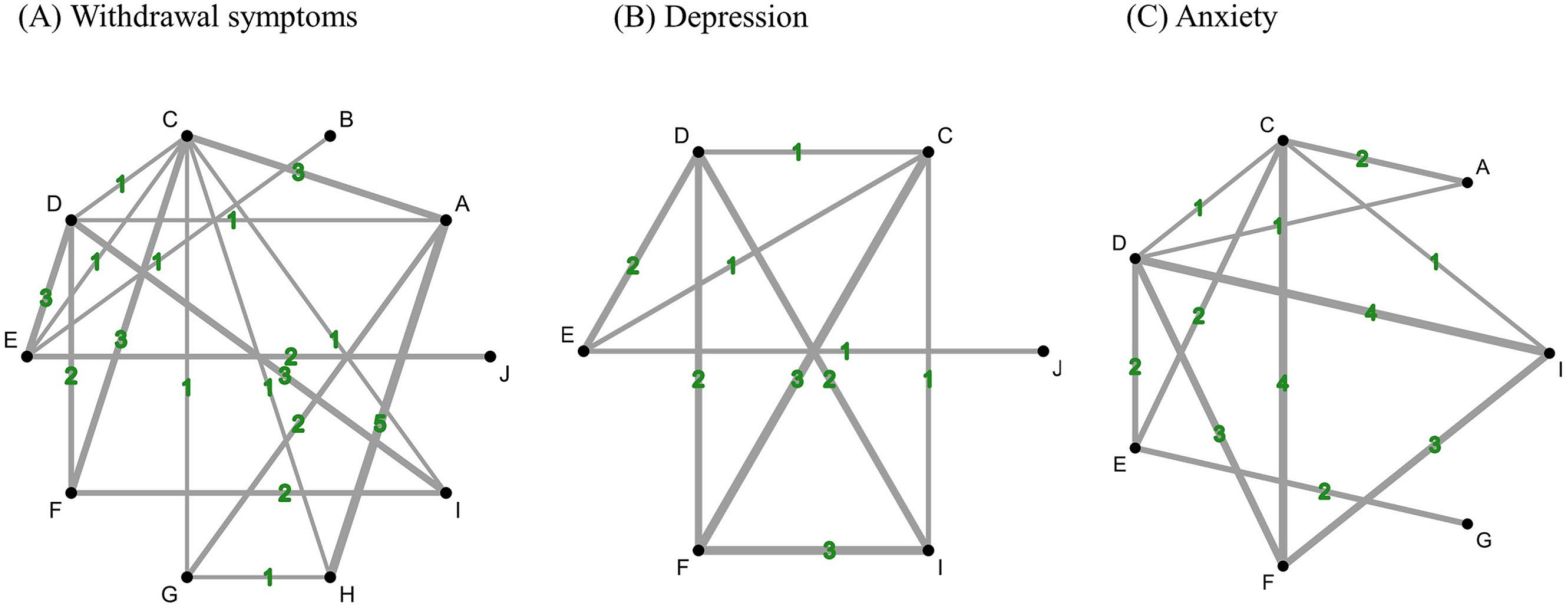
Network evidence diagram for different outcomes. A: Western medicine, B: Warm acupuncture, C: Acupuncture therapies, D: Electronic stimulation, E: Usual care, F: No treatment, G: Acupuncture therapies + Western medicine, H: Electronic stimulation + Western medicine, I: Placebo, J: Acupuncture therapies + Usual care.

For the NMA for withdrawal symptoms, we evaluated 10 interventions (Figure 4A). Based on the inconsistency test, the consistency model could be accepted (*p* = 0.9749). Based on the SUCRA ranking (Table 2), the priorities in terms of effectiveness measures for withdrawal symptoms were as follow: (1) Acupuncture therapies + Usual care, (2) Electronic stimulation, (3) Acupuncture therapies, (4) Warm acupuncture, (5) Acupuncture therapies + WM, (6) Electronic stimulation + WM, (7) WM, (8) Placebo, (9) Usual care, and (10) No treatment. According to the netleague table showing each comparative effectiveness of treatments, Acupuncture therapies + WM (SMD −2.33, 95% CI −4.12 to −0.54) and Electronic stimulation (SMD −1.51, 95% CI −2.81 to −0.22) were both significantly inferior to Usual care. Moreover, Acupuncture therapies + WM (SMD −2.76, 95% CI −5.26 to −0.26), Electronic stimulation (SMD −1.94, 95% CI −3.27 to −0.61), and Acupuncture therapies (SMD −1.33, 95% CI −2.55 to −0.11) were significantly superior to no treatment. Compared to placebo, Electronic stimulation (SMD, −1.41, 95% CI −2.64 to −0.17) was significantly superior. However, no significant differences were observed in the remaining comparisons (Table 3). The inconsistency assessment revealed substantial heterogeneity within the network. Consequently, we conducted subgroup analyses based on specific treatment types to explore potential sources of variation (Supplementary material 3A).

**Table 2:** SUCRA ranking.

Intervention	Withdrawal symptoms		Depression		Anxiety	
Acupuncture therapies + Usual care	1	89.7	3	59.1	2	54.2
Electronic stimulation	2	80.1	1	79.8	4	49.3
Acupuncture therapies	3	61.9	2	78.9	5	48.2
Warm acupuncture	4	59.2				
Acupuncture therapies + WM	5	57.8				
Electronic stimulation + WM	6	45.4				
WM	7	32.6			3	53.0
Placebo	8	31.6	5	22.1	7	42.7
Usual care	9	27.6	4	55.4	1	57.2
No treatment	10	14.0	6	4.8	6	45.4

**Table 3:** Netleague table for withdrawal symptoms (SMD, 95%CI).

Acupuncture therapies + Usual care	Electronic stimulation	Acupuncture therapies	Warm acupuncture	Acupuncture therapies + WM	Electronic stimulation + WM	WM	Placebo	Usual care	No treatment
Acupuncture therapies + Usual care								−**2.33 [−4.12; −0.54]**	
−0.82 [−3.03; 1.39]	Electronic stimulation	−0.74 [−3.23; 1.76]				−0.07 [−2.53; 2.39]	−1.68 [−3.14; −0.23]	**−1.72 [−3.16; −0.28]**	−1.46 [−3.24; 0.32]
−1.43 [−3.83; 0.97]	−0.61 [−1.87; 0.66]	Acupuncture therapies		−0.16 [−2.64; 2.33]	−1.19 [−3.68; 1.30]	−1.13 [−2.56; 0.30]	0.14 [−2.35; 2.62]	−0.19 [−2.83; 2.45]	**−1.46 [−2.90; −0.02]**
−1.30 [−4.37; 1.78]	−0.48 [−3.29; 2.33]	0.13 [−2.83; 3.10]	Warm acupuncture			.		−1.03 [−3.53; 1.46]	
−1.48 [−4.43; 1.47]	−0.66 [−2.76; 1.45]	−0.05 [−1.90; 1.80]	−0.18 [−3.61; 3.24]	Acupuncture therapies + WM	−1.03 [−3.52; 1.46]	−0.72 [−2.47; 1.04]			
−1.85 [−4.60; 0.91]	−1.03 [−2.83; 0.78]	−0.42 [−1.94; 1.11]	−0.55 [−3.80; 2.70]	−0.37 [−2.16; 1.42]	Electronic stimulation + WM	−0.44 [−1.55; 0.67]			
−2.14 [−4.72; 0.45]	−1.32 [−2.85; 0.22]	−0.71 [−1.93; 0.52]	−0.84 [−3.95; 2.27]	−0.66 [−2.26; 0.95]	−0.29 [−1.37; 0.79]	WM			
−2.23 [−4.71; 0.25]	**−1.41 [−2.64; −0.17]**	−0.80 [−2.19; 0.59]	−0.93 [−3.96; 2.09]	−0.75 [−2.98; 1.48]	−0.38 [−2.34; 1.58]	−0.09 [−1.81; 1.63]	Placebo		−0.66 [−2.43; 1.10]
**−2.33 [−4.12; −0.54]**	**−1.51 [−2.81; −0.22]**	−0.90 [−2.50; 0.69]	−1.03 [−3.53; 1.46]	−0.85 [−3.20; 1.49]	−0.48 [−2.57; 1.60]	−0.20 [−2.06; 1.67]	−0.10 [−1.81; 1.61]	Usual care	
**−2.76 [−5.26; −0.26]**	**−1.94 [−3.27; −0.61]**	**−1.33 [−2.55; −0.11]**	−1.46 [−4.50; 1.58]	−1.28 [−3.44; 0.88]	−0.91 [−2.80; 0.97]	−0.62 [−2.26; 1.01]	−0.53 [−1.88; 0.82]	−0.43 [−2.16; 1.31]	No treatment

The comparison must be read from left to right. * The bolding result means there was a statistically significant difference between the interventions and controls. In the case of effect, when the standard mean difference is < 0, it means the effectiveness of the column treatment is better; otherwise, it is not.

##### 3.4.1.3 Subgroup analysis

**Figure 5:**
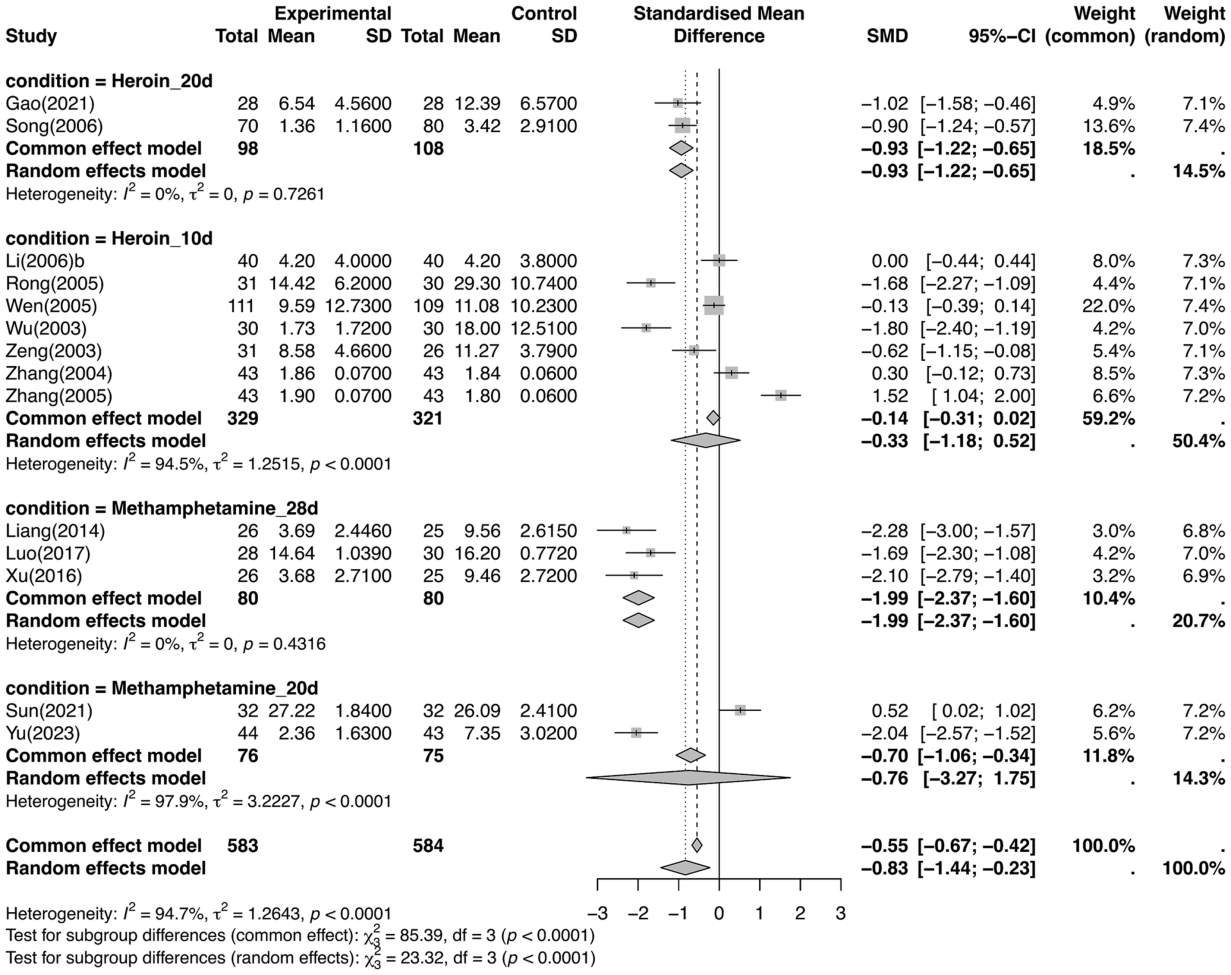
Subgroup analysis based on drug type addiction and treatment duration (withdrawal symptoms).

Moreover, to assess the effectiveness of a specific intervention, we performed subgroup meta-analyses for each comparison (Supplementary material 4). Nine comparisons showed significant difference between intervention and comparison groups: (1) TEAS vs. Placebo (sham_TEAS) (SMD −2.38, 95% CI −3.78 to −0.99; *p* = 0.0008, *I*^2^ = 88%), (2) AA vs. No treatment (SMD −1.38, 95% CI −2.05 to −0.71; *p* < 0.0001, *I*^2^ = 79%), (3) Placebo (Sham_EA) vs. No treatment (SMD −0.65, 95% CI −1.05 to −0.25; *p* = 0.001, *I*^2^ = 0%), (4) EA vs. Usual care (MD −4.78, 95% CI −5.47 to −4.08; *p* < 0.0001, *I*^2^ = 0%), (5) MA + WM vs. MA (MD 1.60, 95% CI 0.28 to 2.92, *p* = 0.03), (6) MA vs. TEAS + WM (MD, −12.04, 95% CI −17.63 to −6.45, *p* < 0.0001), (7) EA vs. AA (MD −2.04, 95% CI −3.58 to −0.50, *p* = 0.009), (8) EA vs. No treatment (SMD, −1.05, 95% CI −1.49 to −0.60; *p* < 0.00001, *I*^2^ = 27%), and (9) MA + WM vs. TEAS+WM (MD −10.44, 95% CI −16.11 to −4.77, *p* = 0.0003). Furthermore, we assessed 13 interventions (Supplementary material 5A) for NMA. The consistency model was accepted (*p* = 0.9917) based on the inconsistency test. The SUCRA ranking probability of different intervention measures in withdrawal symptoms was as follows: (1) TEAS, (2) MA, (3) MA + WM, (4) EA + WM, (5) EA, (6) AA+ Usual care, (7) TEAS+WM, (8) WM, (9) Placebo, (10) WA, (11) No treatment, (12) Usual care, (13) AA (Table 4). According to the netleague table, TEAS was significantly superior to Placebo, no treatment, or usual care. Meanwhile, EA and AA+ Usual care were also both significantly superior to usual care. EA had a significantly better effect than no treatment (Supplementary material 6). In the subgroup analysis, we categorized interventions into specific modalities (AA, EA, and MA). Sensitivity analyses showed similar results, except when the study by Zhao et al. (1997) was removed (Supplementary material 7). In the node-splitting analysis, the result showed that EA showed the most pronounced therapeutic effect compared to usual care and no treatment. Although high heterogeneity persisted in some MA-related comparisons, the consistency between direct and indirect estimates was maintained across most nodes (Supplementary material 3B). To explore the potential sources of heterogeneity, a meta-regression was conducted using duration (days) as a continuous moderator. The regression analysis revealed a significant negative association between the intervention duration and treatment effect SMD. As illustrated in Supplementary material 8, a longer duration was associated with a larger treatment effect (a more negative SMD). Specifically, studies with a shorter duration (around 10 days) showed a relatively small effect size, whereas studies extending beyond 75 days exhibited a substantially enhanced effect (SMD < −3). The bubble sizes, representing the precision (1/SE) of each study, indicate that the regression model appropriately accounts for the weighting of more precise trials. To further investigate the sources of heterogeneity, we performed pairwise meta-analysis by strictly matching the drug type and treatment duration. While significant heterogeneity persisted in the short-term treatment groups, it was completely resolved (*I*^2^ = 0%) when analyzing specific long-term durations. Specifically, no heterogeneity was observed in the heroin group with a 20-day duration and the methamphetamine group with a 28-day duration (Figure 5).

**Table 4:** SUCRA ranking for subgroup analysis.

Intervention	Withdrawal symptoms		Depression		Anxiety	
TEAS	1	84.5	4	44.7	7	50.2
MA	2	75.0	6	43.7	4	53.3
MA + WM	4	65.1				
EA + WM	5	60.6				
EA	6	54.6	2	61.2	5	52.8
AA+ Usual care	3	71.1			1	56.5
TEAS+WM	7	47.4				
WM	8	46.7			2	55.6
Placebo	11	36.0	5	44.0	8	46.8
WA	9	42.7				
No treatment	12	16.0	8	42.7	9	43.2
Usual care	13	12.0	3	58.2	3	54.1
MA + Usual care			1	62.6	6	50.2
AA	10	38.6	7	42.9	10	40.9

AA, auricular acupuncture; MA, Manual acupuncture; EA, electro acupuncture; TEAS, transcutaneous electric acupoint stimulation; WM, Western medicine; WA, Warm acupuncture.

#### 3.4.2 Depression (secondary outcome)

##### 3.4.2.1 Pairwise meta-analysis

The results of the pairwise meta-analysis showed that depression symptoms were significantly reduced in the following comparisons: (1) Acupuncture vs. Placebo (MD, −4.80 [−7.59, −2.01], *p* = 0.0007); (2) Acupuncture vs. No treatment (MD, −3.61 [−5.83, −1.40], *p* = 0.001, *I*^2^ = 95%); (3) Electronic stimulation vs. No treatment (SMD, −1.22 [−2.45, −0.00], *p* = 0.05, *I*^2^ = 89%); (4) Electronic stimulation vs. Usual care (MD, −4.13 [−5.84, −2.42], *p* < 0.00001, *I*^2^ = 21%); (5) Electronic stimulation vs. Acupuncture (MD, −2.11 [−3.74, −0.48], *p* = 0.01); and (6) Placebo vs. No treatment (SMD, −0.46 [−0.77, −0.15], *p* = 0.004, *I*^2^ = 0%). However, the remaining comparisons did not show statistically significant differences (Supplementary material 9).

##### 3.4.2.2 NMA for depression

NMA for depression was used to evaluate six interventions (Figure 4B). The consistency model was accepted (*p* = 0.0778) based on the inconsistency test. Based on the SUCRA ranking (Table 2), the priorities in terms of the effectiveness of the measures for depression were as follows: (1) Electronic stimulation, (2) Acupuncture, (3) Acupuncture + Usual care, (4) Placebo, (5) Usual care, (6) No treatment. Moreover, according to the netleague table showing each comparative effectiveness of treatments, compared to no treatment, Electronic stimulation (SMD, −2.96 [−4.36; −1.56]), Acupuncture therapies (SMD, −2.96 [−4.28; −1.63]), and Usual care (SMD, −2.18 [−4.13; −0.23]) showed a significant difference. Compared with Placebo, Electronic stimulation (SMD, −2.28 [−3.69; −0.87]) and Acupuncture therapies (SMD, −2.28 [−3.77; −0.78]) had better effects (Supplementary material 10). Given the evidence of significant inconsistency and heterogeneity within the global network, we performed stratified analyses by treatment modality to identify the underlying drivers of this variability (Supplementary material 11A).

##### 3.4.2.3 Subgroup analysis

Moreover, to assess the effectiveness of a specific intervention, we also compared each intervention. The meta-analysis results on depression showed that EA vs. Usual care (MD, −4.14 [−5.66, −2.62], *p* < 0.0001, *I*^2^ = 21%), AA vs. No treatment (MD, −10.10 [−14.30, −5.89] *p* < 0.00001, *I*^2^ = 88%), MA vs. No treatment (MD, −12.74 [−13.66, −11.82], *p* < 0.00001), MA vs. Placebo (Sham_MA) (MD, MD, −4.80 [−7.59, −2.01], *p* = 0.0007), EA vs. AA (MD, MD, −4.39 [−6.11, −2.67], *p* < 0.00001), and Placebo (sham_EA) vs. No treatment (MD, −0.46 [−0.77, −0.15], *p* = 0.004, *I*^2^=0%) showed significant differences (Supplementary material 4). Meanwhile, in the NMA on depression, we evaluated eight interventions. The consistency model was deemed acceptable (*p* = 0.9836) (Supplementary material 5B), based on the test of inconsistency. The SUCRA ranking probability of different intervention measures for depression was as follows: (1) MA + Usual care, (2) EA, (3) Usual care, (4) TEAS, (5) Placebo, (6) MA, (7) AA, and (8) No treatment (Supplementary material 5). According to the netleague table, EA, MA, and TEAS were significantly superior to Placebo. EA, MA, AA, and Usual care were significantly better than No treatment (Supplementary material 12). The findings remained largely unchanged in the sensitivity analyses, with the exception of cases where Li et al. (2012) or Chen (2015) were removed (Supplementary material 12). Despite stratifying the network by treatment characteristics, high levels of within-subgroup heterogeneity persisted, particularly in comparisons involving ‘No treatment’ controls. These results indicate that treatment-specific factors only partially account for the overall network-wide disparity (Supplementary material 11B). Thus, we performed the additional meta-regression analysis based on the treatment duration. Network meta-regression analysis was conducted to explore the impact of treatment duration on the observed efficacy. A negative linear trend was observed, indicating that the SMD tended to decrease as the duration of treatment increased. While the regression slope (beta) varied across treatment comparisons, the overall direction suggested a potential reduction in effect size over time. Notably, study-level estimates were widely dispersed around the regression line, and the 95% confidence intervals widened substantially for interventions exceeding 60 days, reflecting increased uncertainty in long-term data (Supplementary material 14). Meanwhile, we performed a pairwise meta-analysis by strictly matching the drug type and treatment duration. Subgroup analysis based on drug type and treatment duration revealed a significant effect in the Methamphetamine Short-term treatment group (SMD = −0.72; 95% CI [−1.08, −0.37]). However, the overall pooled effect size did not reach statistical significance in the random-effects model (SMD = −0.70; 95% CI [−1.69, 0.29]). Although categorizing treatment duration partially explained the variance, high heterogeneity persisted in the heroin long-term treatment group (*I*^2^ = 97.1%, *p* < 0.0001), suggesting that other moderating factors beyond treatment duration may influence outcomes (Supplementary material 15).

#### 3.4.3 Anxiety (secondary outcome)

##### 3.4.3.1 Pairwise meta-analysis

The results of the pairwise meta-analysis showed that anxiety symptoms were significantly reduced in the following comparisons: (1) Acupuncture vs. No treatment (SMD, −2.94 [−3.92, −1.97], *p* < 0.00001, *I*^2^ = 86%); (2) Acupuncture vs. Placebo (MD, −7.80 [−11.49, −4.11], *p* < 0.00001); (3) Acupuncture + WM vs. +WM (MD, −2.32 [−3.35, −1.29], *p* < 0.00001); (4) Electronic stimulation vs. WM (MD, −4.58 [−7.76, −1.40], *p* = 0.005); (5) Electronic stimulation +WM vs. WM (MD, −7.95 [−8.86, −7.04], *p* < 0.00001); (6) Electronic stimulation vs. Placebo (SMD, −1.40 [−2.33, −0.48], *p* = 0.003, *I*^2^ = 93%); (7) Electronic stimulation vs. Usual care (MD, −2.62 [−4.60, −0.64], *p* = 0.010, *I*^2^ = 75%); and (8) Electronic stimulation vs. Acupuncture (MD, −4.39 [−6.11, −2.67], *p* < 0.00001) (Supplementary material 16).

##### 3.4.3.2 NMA on anxiety

For the NMA on anxiety, we estimated seven interventions (Figure 4C). The consistency model was accepted (*p* = 0.1000). Based on the SUCRA ranking (Table 2), the priorities in terms of effectiveness measures by anxiety were as follows: (1) Usual care, (2) Acupuncture + Usual care, (3) WM, (4) Electronic stimulation, (5) Acupuncture, (6) Placebo, and (7) No treatment. However, according to the netleague table showing each comparative effectiveness of treatments, compare to no treatment, Acupuncture therapies + Usual care (SMD, −1.98 [−3.79; −0.17]), Electronic stimulation (SMD, −1.67 [−2.62; −0.72]), Acupuncture therapies (SMD, −1.49 [−2.29; −0.69]), and Usual care (SMD, −1.27 [−2.50; −0.05]) were significantly different from Placebo. Acupuncture therapies + Usual care (SMD, −2.54 [−4.33; −0.75]), Electronic stimulation (SMD, −2.23 [−3.04; −1.41]), Acupuncture therapies (SMD, −2.04 [−2.88; −1.21]), and Usual care (SMD, −1.83 [−3.02; −0.64]), and WM (SMD, −1.44 [−2.74; −0.14]) were statistically significantly better than No treatment (Supplementary material 17). However, given the substantial heterogeneity identified in the initial assessment, the data were further partitioned into subgroups based on treatment to achieve a more nuanced understanding of the results (Supplementary material 18A).

##### 3.4.3.3 Subgroup analysis

To assess the specific intervention, we also compared each intervention. The meta-analysis results on anxiety showed that AA vs. No treatment (MD, −6.95 [−8.65, −5.24], *p* < 0.0001, *I*^2^ = 45), MA vs. No treatment (MD, −15.07 [−26.23, −3.92], *p* = 0.0008, *I*^2^ = 99), EA vs. WM (MD, −4.58 [−7.76, −1.40], *p* = 0.005), MA vs. WM (MD, −3.64 [−6.99, −0.29], *p* = 0.03), and MA + WM vs. WM (MD, MD, −2.32 [−3.35, −1.29], *p* < 0.00001), MA vs. Placebo (Sham_MA) (MD, −7.80 [−11.49, −4.11], *p* < 0.0001), EA vs. AA (MD, −2.11 [−3.74, −0.48], *p* = 0.01), AA + Usual care vs. Usual care (MD, −4.87 [−6.86, −2.88], *p* < 0.00001); and EA vs. Usual care (MD, −2.62 [−4.60, −0.64], p = 0.01, *I*^2^ = 45) showed significant differences (Supplementary material 4).

Meanwhile, we estimated 10 interventions (Supplementary material 5C) in this NMA. The consistency model was accepted (*p* = 1.0000) based on the inconsistency test. The SUCRA ranking probability of different intervention measures regarding withdrawal symptoms was as follows: (1) AA + Usual care, (2) WM, (3) Usual care, (4) MA, (5) EA, (6) MA + Usual care, (7) TEAS, (8) Placebo, (9) No treatment, and (10) AA (Table 4). According to the netleague table, the results showed that MA and TEAS were both significantly better than Placebo. MA, TEAS, EA, and WM were significantly superior to no treatment (Supplementary material 19). The Sensitivity analyses also showed similar results (Supplementary material 20). Despite subgroup analyses by treatment type, substantial heterogeneity persisted across several comparisons (e.g., EA vs. No treatment, *I*^2^ = 96.3%). This suggests that the observed inconsistency in the network may be driven by complex clinical or methodological factors beyond the treatment categories themselves (Supplementary material 18B). Meta-regression analysis was performed to evaluate the impact of treatment duration on clinical outcomes. Although a trend toward greater efficacy (lower SMD) was observed with increasing treatment duration (Supplementary material 21). In the subgroup analyses for anxiety, the Methamphetamine Short-term treatment subgroup (SMD = −0.87; 95% CI [−1.17, −0.57]) and the Heroin Short-term treatment subgroup (SMD = −0.85; 95% CI [−1.32, −0.37]) groups demonstrated statistically significant reductions in anxiety levels. However, the Heroin Long-term treatment subgroup (SMD = −0.99; 95% CI [−3.26, 1.28]) and the opioids short-term subgroup did not reach statistical significance. Despite the duration-based stratification, substantial heterogeneity remained in the overall model (*I*^2^ = 90.6%, *p* < 0.0001), particularly within the Heroin Long-term subgroup (*I*^2^ = 97.1%) and Heroin Short-term subgroups (*I*^2^ = 86.5%) categories (Supplementary material 22).

### 3.5 Publication bias

The funnel plots were drawn according to the withdrawal symptoms, depression, and anxiety outcomes. The results of Egger’s test were 0.2915, 0.4852, and 0.3175, respectively (Supplementary materials 23–25). Furthermore, the results showed there is no publication bias.

### 3.6 Adverse events

Twelve studies assessed adverse events (Wu et al., 2003; Zhang et al., 2004; Zhang et al., 2005; Wen et al., 2005; Li et al., 2006b; Li et al., 2012; Wang, 2020; Gu et al., 2023; Sun et al., 2021; Zhu et al., 2008; Zhao et al., 1997; Chan et al., 2014). For MA and EA, the most commonly reported adverse events were gastrointestinal dysfunction, such as nausea and vomiting. Other reported adverse events included needle syncope. Detailed information on adverse events is provided in Table 5.

**Table 5:** Detail of adverse events.

Study	MA	EA + WM	WM	EA	MA + WM	Placebo	AA	AA+ Usual care
Zhao et al. (1997)			Dizziness, nausea, vomiting, sweating, palpitations, dry mouth, poor sleep, fatigue (18)	None				
Wu et al. (2003)	Dry month (1)	Dizziness (2); Headache (1); Nausea (1); Dry month (3); Constipation (1)	Dizziness (2); Headache (1); Dry month (3); Constipation (2) Hypotension (1); Breathing slowed (1); Excited (1)		Dizziness (1) Headache (1) Dry month (2); Constipation (1)			
Zhang et al. (2004)		None	Vomiting (8); Sweating, dilated pupils, blurred vision (6); Hallucination (2); Respiratory depression (5)					
Wen et al. (2005)	None			None				
Zhang et al. (2005)		Local muscular spasm (26)	Nausea and vomiting (8) perspiration, platycoria, and blurred vision (6) hallucination (2) respiratory inhibition (5)					
Li et al. (2006b)	Nausea and vomiting (2); Headache, dizziness (2) Hypotension (1) Constipation (5)		Nausea and vomiting (4) Headache, dizziness (3) Hypotension (4) Constipation (14)					
Li et al. (2012)	Needle syncope (1) Subcutaneous pain (2) Erythema (1)					Nausea (1); Flustered (1) Subcutaneous hematoma (1)		
Chan et al. (2014)						Slight bleeding (1)	Slight bleeding (2)	
Wang (2020)						Pain (1)		
Gu et al. (2023)				Needle syncope (1) Stuck needle (1)				
Sun et al. (2021)								Erythema (1); Pain (1); Cyanosis (1)
Zhu et al. (2008)				None			None	

AA, auricular acupuncture; MA, Manual acupuncture; EA, electroacupuncture; TEAS, transcutaneous electric acupoint stimulation; WM, Western medicine.

### 3.7 Quality of evidence

Based on the GRADE pro level method of the NMA, withdrawal symptoms were predominantly moderate to very low, owing to the lack of blinding in acupuncture therapies. The results of the quality of evidence analysis are presented in Supplementary material 26.

## 4 Discussion

### 4.1 Summary of evidence

This study provides a comprehensive evaluation of the effectiveness of 15 acupuncture-related therapies commonly used in clinical practice for treating drug addiction. We included 35 studies involving 2,812 patients in the pair-wise meta-analysis and NMA on three outcomes. In the pair-wise meta-analysis, consistent with a previously published systematic review (Ge et al., 2020), we also found that compared with no treatment or placebo control, acupuncture-related therapies were associated with improvements in withdrawal symptoms, anxiety, and depression (Supplementary materials 2, 3, 12). In subgroup analyses, AA and MA showed differences in the treatment effect between no treatment and Usual care according to three outcomes. In the NMA, Acupuncture + Usual care showed the highest ranking according to three outcomes. We also performed a subgroup analysis to determine the ranking of specific methods. The results showed that TEAS, MA, and AA + Usual care were the top three methods that improved the withdrawal symptoms. In addition, treatment duration might have a potential role in treating drug addiction. Especially, for withdrawal symptoms, the long-term treatment (more than 20 days) had a certain effect, compared with short-term treatment. Adverse events related to acupuncture included gastrointestinal dysfunction, with fewer reported events compared with those on adverse events associated with WM.

### 4.2 Implications

The therapeutic ‘dose’ of acupuncture is determined by complex stimulation parameters, including force, intensity, frequency, and overall treatment schedule (sessions and duration) (Ma, 2020). In this study, we specifically explored the impact of treatment duration by comparing short-term and long-term treatment. Regarding withdrawal symptoms, short-term treatments exhibited high heterogeneity, whereas the long-term treatment subgroup demonstrated a potentially beneficial effect with markedly lower heterogeneity, suggesting that extended duration may provide more consistent relief for withdrawal. However, for secondary outcomes such as depression and anxiety, the results across duration-based subgroups remained inconsistent and lacked statistical robustness. These disparities suggest that psychiatric symptoms may be influenced by a broader range of confounding factors beyond treatment length. Consequently, further high-quality studies are warranted to confirm these preliminary findings and establish optimized treatment.

The biological plausibility of acupuncture’s effect on drug addiction can be explained through its neuroprotective and homeostatic regulatory mechanisms. Chronic heroin exposure—a potent semi-synthetic opioid—is known to induce significant neuronal loss in key brain reward centers, such as the hippocampus and ventral tegmental area (VTA) (Doyle and Mazei-Robison, 2021). Previous experimental evidence (Gao et al., 2017) has demonstrated that such neuronal damage is often accompanied by the downregulation of mRNA and protein expression for ER stress-related markers, including PERK, eIF2α, CHOP, IRE1, and JNK. Acupuncture may exert its therapeutic effects by modulating these very pathways. Specifically, stimulation of acupoints has been suggested to restore cellular proteostasis and mitigate ER stress-induced apoptosis, thereby protecting neurons within the VTA-hippocampal circuit. This biological restoration may account for the clinical improvements observed in our study, particularly regarding the significant reduction in withdrawal and anxiety symptoms in the short-term methamphetamine and heroin subgroups. Furthermore, the instability and high heterogeneity found in our anxiety and depression analyses might reflect the varied levels of baseline neurological damage across different opioid-dependent populations. While acupuncture provides a promising non-pharmacological intervention, the degree of its efficacy may depend on the extent to which these molecular pathways, such as the PERK/eIF2α signaling, can be successfully reactivated.

The intervention methods, MA + Usual care and AA + Usual care, were found to improve withdrawal symptoms, depression, and anxiety symptoms. MA has been shown to modulate the mesolimbic dopamine pathway and influence the reinstatement and extinction of drug seeking (Lee et al., 2021). Chronic drug use leads to functional impairment of the Prefrontal Cortex (PFC), thereby undermining executive control and decision-making. Acupuncture may promote neurogenesis and synaptic plasticity within the PFC, effectively ‘restoring’ the top-down inhibitory control over drug-seeking behaviors (Yang et al., 2008). This prefrontal recovery provides a biological basis for the long-term stabilization of addiction symptoms and may explain the varied treatment outcomes based on intervention duration. Additionally, AA is a complementary and alternative treatment method that utilizes acupoints located in the ear, which are different from the traditional acupoints. This can increase the level of opiate-like compounds and the interrelationship between endogenous opiate-like peptides and plasma adrenocorticotropin levels in the morphine addicted mice model (Ng, 1996). Usual care is one of the most common methods to treat drug addiction (Volkow and Blanco, 2023). Thus, AA or MA combined with Usual care might act synergistically to improve symptoms.

The best treatment choice varied depending on the symptoms. For example, TEAS may be a preferred treatment method for withdrawal symptoms. TEAS combines transcutaneous electrical nerve stimulation (TENS) and the traditional acupuncture method. It involves the electronic stimulation of the skin at specific acupoints (Zhou et al., 2024). TENS effectively alleviates chronic hypersensitivity by modulating both peripheral and central pain pathways (Vera-Portocarrero et al., 2013). The therapeutic efficacy of TENS is largely attributed to the activation of endogenous opioid systems, whereby different frequencies trigger specific opioid receptor subtypes (Vera-Portocarrero et al., 2013). This mechanism provides a clear biological plausibility for our results, which TEAS might also improve overall symptoms. However, MA + Usual care had a better effect on depression. Decreased serotonergic neuronal activity plays an important role in depression. Gamma-aminobutyric acid (GABA) receptors are localized and interact with serotonergic neurons in the dorsal raphe nucleus (Kumar et al., 2013). MA can modulate the ventral tegmental area of the GABA neurons. However, in terms of anxiety, except for the AA+ Usual care, acupuncture-related therapies showed no better effects than WM. Anxiety is a typical symptom of drug addiction. Acupuncture-related therapy may not improve anxiety symptoms.

PC6 is the most common acupoint in the traditional acupuncture therapies for various diseases. Based on the traditional theory, upward qi of the stomach leads to nausea and vomiting, and PC6 subdues the rebellious energy of the stomach by redirecting the Qi counterflow (Zotelli et al., 2014). Furthermore, in a morphine-sensitive rat model following repeated morphine administration, Fos-like immunoreactivity in the nucleus accumbens was significantly suppressed in the striatum and nucleus accumbens by microdialysis after PC6 acupoint stimulation (Kim et al., 2005). Regarding the acupoint of AA, TF4 (Shenmen) was the most commonly used acupoint. TF4 (Shenmen) functions to relieve anxiety (Qu et al., 2014). Therefore, this acupoint has the potential to improve drug addiction symptoms.

### 4.3 Strengths and limitations

This systematic review and NMA have several strengths. We searched all relevant studies by searching international, Korean, and Chinese databases. Thus, not only did we review the Chinese studies, but also those published in the USA and Sweden. In clinical practice, physicians prefer to use different types of acupuncture to improve the symptoms and complications of drug addiction, and no standard guideline on acupuncture-related therapies for drug addiction exists. To the best of our knowledge, we evaluated 15 different types of treatments (including MA, EA, WA, TEAS, usual care, no treatment, WM, AA, placebo control, MA + WM, EA + WM, TEAS+WM, AA+WM, and MA + Usual care) and performed qualitative and quantitative analyses based on the available data using NMA to examine the comparative advantage of several treatments. In addition, we used GRADEpro to assess the quality of evidence for the primary outcome.

However, this study has some limitations. First, significant heterogeneity was observed in this meta-analysis. Owing to the limited number of studies included, it was difficult to determine the reason for heterogeneity. Second, the methodological quality of the RCT was low because of the characteristics of acupuncture-related therapies. Third, the long-term effect of acupuncture on treating drug addiction could not be determined because the treatment duration was variable, and few follow-up data existed. Moreover, we could not explore the effect of specific acupoint in this study, due to the acupoints were also variable. Fourth, the interpretation of our findings, particularly the treatment rankings derived from SUCRA values, requires careful consideration. The use of SMD was necessary due to the diverse clinical scales employed across studies; however, this approach may limit the direct clinical interpretability compared to MD. In addition, despite conducting sensitivity analyses, the small number of studies per comparison and the inherent methodological heterogeneity may affect the robustness and stability of the network estimates. As such, these rankings should be viewed as indicative of potential trends rather than definitive clinical superiority. Fifth, there may be potential bias related to population genetics and cultural differences, because there were only two non-Chinese researchers. Lastly, potential biases related to population genetics and cultural disparities among the included cohorts were not fully controlled, which may influence the generalizability of the results. Future large-scale, standardized trials are essential to confirm these findings with greater certainty. Finally, although we searched 10 databases, the sample size was small.

### 4.4 Future studies

Further well-designed RCTs with high-quality evidence are required to assess the efficacy of acupuncture-related therapies for drug addiction. Moreover, RCTs should blind the participants and physicians to reduce bias and should be prospectively registered in the clinical trial platform to reduce publication bias. Additionally, international multicenter RCTs should be performed to include follow-up with long-term treatment duration.

## 5 Conclusion

Based on low-to-moderate quality evidence, this study indicates that acupuncture-related therapies, particularly when integrated with usual care, are associated with improvements in overall addiction, withdrawal, and psychiatric symptoms. While TEAS, MA, and AA showed favorable trends across specific symptom domains, the overall certainty of these findings remains limited. Consequently, these results should be interpreted with caution, and further high-quality research is required to clarify the clinical role of acupuncture in addiction recovery.
