# Supplementary material for: Acupuncture-related therapies for drug addiction: a systematic review and network meta-analysis
Source: Front Hum Neurosci. 2026 May 20;20:1800440. doi: 10.3389/fnhum.2026.1800440 (PMC13230095; doi:10.3389/fnhum.2026.1800440)
Supplement: Supplementary file 1 [file Data_Sheet_1.docx]

Supplementary Material

# Supplementary Material 1. Search strategy

# Supplementary Material 2. Forest plot of withdrawal symptoms

# Supplementary Material 3. Forest plot of the node-splitting analysis for inconsistency assessment (Withdrawal symptoms)

# Supplementary Material 4. Pairwise meta-analysis in subgroup analysis

# Supplementary Material 5. Network evidence diagram of different outcome in subgroup analysis

# Supplementary Material 6. Netleague table for withdrawal symptoms in subgroup analysis (SMD, 95% CI)

# Supplementary Material 7. Sensitivity analysis for withdrawal symptoms in subgroup analysis

# Supplementary Material 8. Meta-regression of withdrawal symptoms

# Supplementary Material 9. Forest plot of depression

# Supplementary Material 10. Netleague table for depression (SMD, 95%CI)

# Supplementary Material 11. Forest plot of the node-splitting analysis for inconsistency assessment (Depression)

# Supplementary Material 12. Netleague table for depression in subgroup analysis (SMD, 95%CI)

# Supplementary Material 13. Sensitivity analysis for Depression in the subgroup analysis

# Supplementary Material 14. Meta-regression of depression

# Supplementary Material 15. Subgroup analysis based on the type of drug and treatment duration (Depression)

# Supplementary Material 16. Forest plot of anxiety

# Supplementary Material 17. Netleague table for anxiety (SMD, 95%CI)

# Supplementary Material 18. Forest plot of the node-splitting analysis for inconsistency assessment (Anxiety)

# Supplementary Material 19. Netleague table for anxiety in subgroup analysis (SMD, 95%CI)

# Supplementary Material 20. Sensitivity analysis for Anxiety in the subgroup analysis

# Supplementary Material 21. Meta-regression of anxiety

# Supplementary Material 22. Subgroup analysis based on the type of drug and treatment duration (Anxiety)

# Supplementary Material 23. Funnel plot of withdrawal symptoms

# Supplementary Material 24. Funnel plot of depression

# Supplementary Material 25. Funnel plot of anxiety

# Supplementary Material 26. Quality of evidence

Supplementary Material 1. Search strategy

1. Search strategy in PubMed

| #1 | "Substance-Related Disorders"[Mesh] |
| --- | --- |
| #2 | “opioid” [Title/Abstract] |
| #3 | “heroin” [Title/Abstract] |
| #4 | “marijuana” [Title/Abstract] |
| #5 | “cocaine” [Title/Abstract] |
| #6 | “methadone” [Title/Abstract] |
| #7 | “narcot” [Title/Abstract] |
| #8 | “detoxify” [Title/Abstract] |
| #9 | “desintoxi” [Title/Abstract] |
| #10 | “disintoxi” [Title/Abstract] |
| #11 | “illicit drug” [Title/Abstract] |
| #12 | “drug addiction” [Title/Abstract] |
| #13 | #1 or #2 or #3 or #4 or #5 or #6 or #7 or #8 or #9 or #10 or #11 or #12 |
| #14 | "Acupuncture"[MeSH Terms] |
| #15 | "acupuncture therapy"[Title/Abstract] |
| #16 | "acupuncture points"[Title/Abstract] |
| #17 | "manual acupuncture"[Title/Abstract] |
| #18 | "Electroacupuncture"[Title/Abstract] |
| #19 | "auricular acupuncture"[Title/Abstract] |
| #20 | "auricular acupressure"[Title/Abstract] |
| #21 | "scalp acupuncture"[Title/Abstract] |
| #22 | "acupoint injection"[Title/Abstract] |
| #23 | "pharmacopuncture"[Title/Abstract] |
| #24 | #14 or #15 or #16 or #17 or #18 or #19 or #20 or #21 or #22 or #23 |
| #25 | "Randomized Controlled Trials as Topic"[MeSH Terms] OR "Controlled Clinical Trials as Topic"[MeSH Terms] |
| #26 | "Randomized"[Title/Abstract] |
| #27 | "Randomly"[Title/Abstract] |
| #28 | #25 or #26 or #27 |
| #29 | #28 and #24 and #13 |

2. Search strategy in EMBASE

| #29 | #28 and #23 and #13 |
| --- | --- |
| #28 | #27 or #26 or #25 or #24 |
| #27 | Randomized |
| #26 | Randomly |
| #25 | ‘Randomized Controlled Trail (topic)’/exp |
| #24 | ‘Controlled Clinical Trial (topic)’/exp OR ‘Controlled Clinical Trial (topic)’ |
| #23 | #22 or #22 or #21 or #20 or #19 or #18 or #17 or #16 or #15 or #14 |
| #22 | pharmacopuncture |
| #21 | auricular AND acupuncture |
| #20 | auricular AND acupressure |
| #19 | acupoint AND injection |
| #18 | Electroacupuncture |
| #17 | acupuncture AND therapy |
| #16 | acupuncture AND points |
| #15 | manual AND acupuncture |
| #14 | ‘Acupuncture’/exp or ‘Acupuncture’ |
| #13 | #12 or #11 or #10 or #9 or #8 or #7 or #6 or #5 or #4 or #3 or #2 or #1 |
| #12 | detoxify |
| #11 | desintoxi |
| #10 | disintoxi |
| #9 | illicit drug |
| #8 | drug addiction |
| #7 | opioid |
| #6 | heroin |
| #5 | marijuana |
| #4 | cocaine |
| #3 | methadone |
| #2 | narcot |
| #1 | 'toxic substance'/exp |

3. Search strategy in Cochrane Library

| #1 | MeSH descriptor: [Substance-Related Disorders] explode all trees |
| --- | --- |
| #2 | detoxify |
| #3 | desintoxi |
| #4 | disintoxi |
| #5 | illicit drug |
| #6 | drug addiction |
| #7 | opioid |
| #8 | heroin |
| #9 | marijuana |
| #10 | cocaine |
| #11 | methadone |
| #12 | narcot |
| #13 | #1 or #2 or #3 or #4 or #5 or #6 or #7 or #8 or #9 or #10 or #11 or #12 |
| #14 | MeSH descriptor: [Randomized Controlled Trails] explode all trees |
| #15 | MeSH descriptor: [Controlled Clinical Trial] explode all trees |
| #16 | Randomized |
| #17 | Randomly |
| #18 | #14 or #15 or #16 or #17 |
| #19 | MeSH descriptor: [Acupuncture] explode all trees |
| #20 | acupuncture therapy |
| #21 | acupuncture points |
| #22 | manual acupuncture |
| #23 | Electroacupuncture |
| #24 | auricular acupuncture |
| #25 | auricular acupressure |
| #26 | scalp acupuncture |
| #27 | acupoint injection |
| #28 | pharmacopuncture |
| #29 | #19 or #20 or #21 or #22 or #23 or #24 or #25 or #26 or #27 or #28 |
| #30 | #29 and #18 and #13 |

4. CNKI

| (SU='鸦片' OR SU='海洛因' OR SU='甲基苯丙胺' OR SU='冰毒' OR SU='吗啡' OR SU='大麻' OR SU='可卡因' OR SU='毒品'OR SU='戒断') AND (SU = '针刺' OR SU='针灸' OR SU='电针' OR SU='头针' OR SU='梅花针' OR SU='针灸' OR SU='穴位注射' OR SU='穴位针刀' OR SU='穴位埋线' OR SU='穴位' OR SU='耳穴') AND (SU= '随机对照' OR SU= '随机对照试验' OR SU= '随机对照研究' OR SU= '随机对照临床研究' OR SU= '随机对照试验研究' OR SU= 'RCT' OR SU= '临床' OR SU= '疗效') |
| --- |

5. Weipu

| (K='鸦片' OR K='海洛因' OR K='甲基苯丙胺' OR K='冰毒' OR K='吗啡' OR K='大麻' OR K='可卡因' OR K='毒品'OR K='戒断') AND (K = '针刺' OR K='针灸' OR K='电针' OR K='头针' OR K='梅花针' OR K='针灸' OR K='穴位注射' OR K='穴位针刀' OR K='穴位埋线' OR K='穴位' OR K='耳穴') AND (K= '随机对照' OR K= '随机对照试验' OR K= '随机对照研究' OR K= '随机对照临床研究' OR K= '随机对照试验研究' OR K= 'RCT' OR K= '临床' OR K= '疗效') |
| --- |

6. Wanfang

| (题名或关键词='鸦片' OR 题名或关键词='海洛因' OR 题名或关键词='甲基苯丙胺' OR 题名或关键词='冰毒' OR 题名或关键词='吗啡' OR 题名或关键词='大麻' OR 题名或关键词='可卡因' OR 题名或关键词='毒品'OR 题名或关键词='戒断') AND (题名或关键词 = '针刺' OR 题名或关键词='针灸' OR 题名或关键词='电针' OR 题名或关键词='头针' OR 题名或关键词='梅花针' OR 题名或关键词='针灸' OR 题名或关键词='穴位注射' OR 题名或关键词='穴位针刀' OR 题名或关键词='穴位埋线' OR 题名或关键词='穴位' OR 题名或关键词='耳穴') AND (题名或关键词= '随机对照' OR 题名或关键词= '随机对照试验' OR 题名或关键词= '随机对照研究' OR 题名或关键词= '随机对照临床研究' OR 题名或关键词= '随机对照试验研究' OR 题名或关键词= 'RCT' OR 题名或关键词= '临床' OR 题名或关键词= '疗效') |
| --- |

7. Sinbio

| ("随机对照"[常用字段:智能] OR "随机对照试验"[常用字段:智能] OR "随机对照研究"[常用字段:智能] OR "随机对照临床研究"[常用字段:智能] OR "随机对照试验研究"[常用字段:智能] OR "RCT"[常用字段:智能] OR "临床"[常用字段:智能] OR "疗效"[常用字段:智能]) AND ("针灸"[常用字段:智能] OR "电针"[常用字段:智能] OR "头针"[常用字段:智能] OR "耳穴"[常用字段:智能] OR "针灸"[常用字段:智能] OR "穴位注射"[常用字段:智能] OR "穴位针刀"[常用字段:智能] OR "穴位埋线"[常用字段:智能] OR "穴位"[常用字段:智能]) AND ("鸦片"[常用字段:智能] OR "海洛因"[常用字段:智能] OR "甲基苯丙胺"[常用字段:智能] OR "冰毒"[常用字段:智能] OR "吗啡"[常用字段:智能] OR "大麻"[常用字段:智能] OR "可卡因"[常用字段:智能] OR "毒品"[常用字段:智能] OR "戒断"[常用字段:智能]) |
| --- |

8. OASIS

| #1 | 제목: (“마약 or 금단현상 or 코카인 or 대마 or 헤로인 or 마리화나 or 대마”) |
| --- | --- |
| #2 | 제목: ("혈위 OR 지압 OR 이압") |
| #3 | 제목: (“임상시험 or 무작위 or 무작위대조군”) |
| #4 | #1 and #2 and #3 |

8. Search strategy in KISS

| #1 | 제목: (“마약 or 금단현상 or 코카인 or 대마 or 헤로인 or 마리화나 or 대마”) |
| --- | --- |
| #2 | 제목: ("혈위 OR 지압 OR 이압") |
| #3 | 제목: (“임상시험 or 무작위 or 무작위대조군”) |
| #4 | #1 and #2 and #3 |

10 Search strategy in KCI

| #1 | 논문제목: (“마약 or 금단현상 or 코카인 or 대마 or 헤로인 or 마리화나 or 대마”) |
| --- | --- |
| #2 | 논문제목: ("혈위 OR 지압 OR 이압") |
| #3 | 논문제목: (“임상시험 or 무작위 or 무작위대조군”) |
| #4 | #1 and #2 and #3 |

Supplementary Material 2. Forest plot of withdrawal symptoms

(A) Acupuncture + WM vs Acupuncture


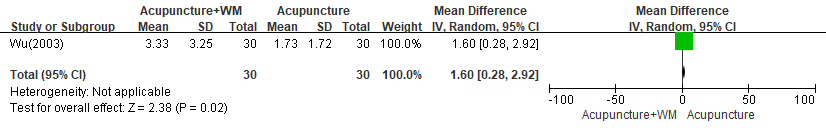


(B) Acupuncture vs No treatment


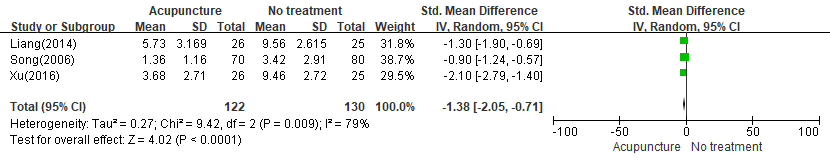


(C) Electronic stimulation + WM vs Acupuncture


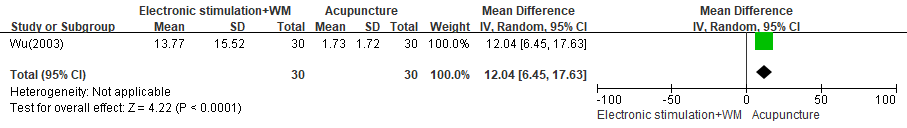


(D) Electronic stimulation + WM vs Acupuncture + WM


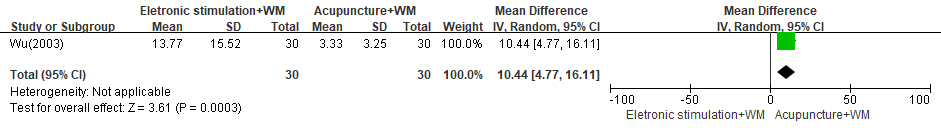


(E) Electronic stimulation vs No treatment


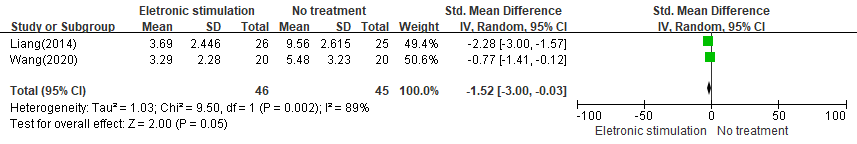


(F) Electronic stimulation vs Placebo


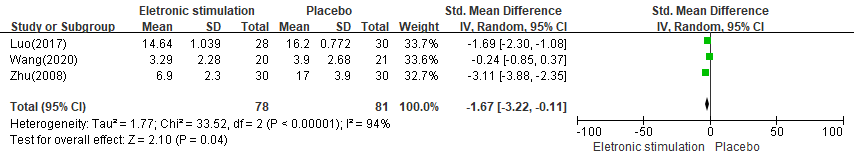


(G) Electronic stimulation vs Usual care


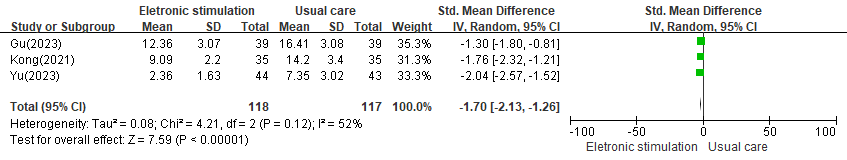


(H) Electronic stimulation vs Acupuncture


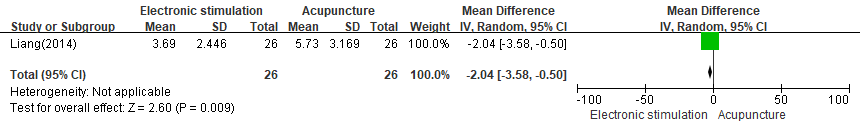


(I) Placebo vs No treatment


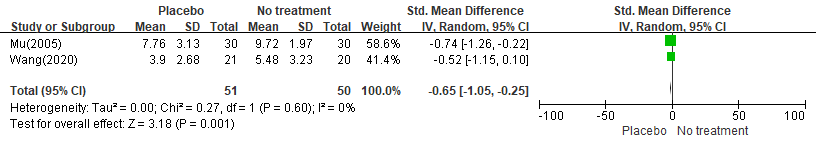


(J) Warm acupuncture vs Usual care


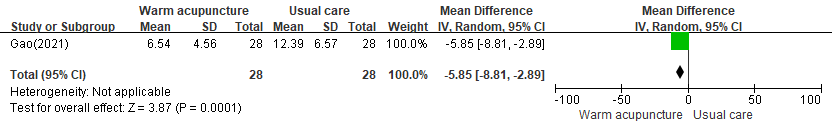


(K) Acupuncture vs WM


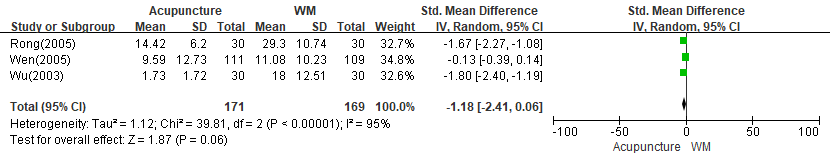


(L) Acupuncture + WM vs WM


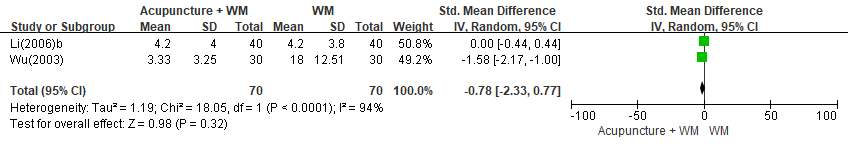


(M) Acupuncture vs Placebo


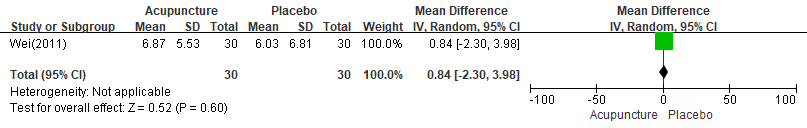


(N) Acupuncture vs Usual care


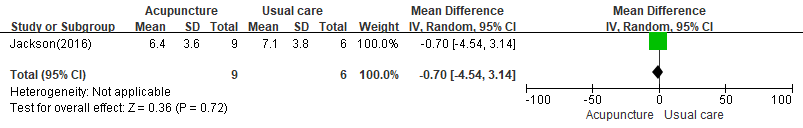


(O) Acupuncture + Usual care vs Usual care


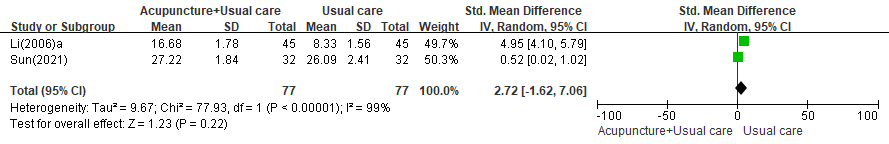


(P) Electronic stimulation vs WM


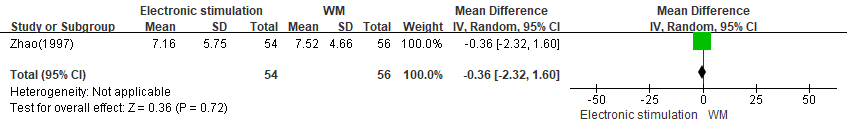


(Q) Electronic stimulation + WM vs WM


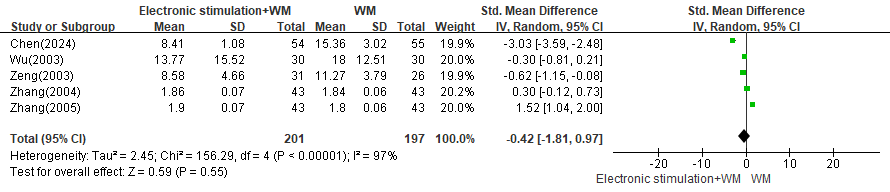


Abbreviation: WM, Western medicine

Supplementary Material 3. Forest plot of the node-splitting analysis for inconsistency assessment (Withdrawal symptoms)

| (A) Primary analysis | (B) Subgroup analysis |
| --- | --- |
| 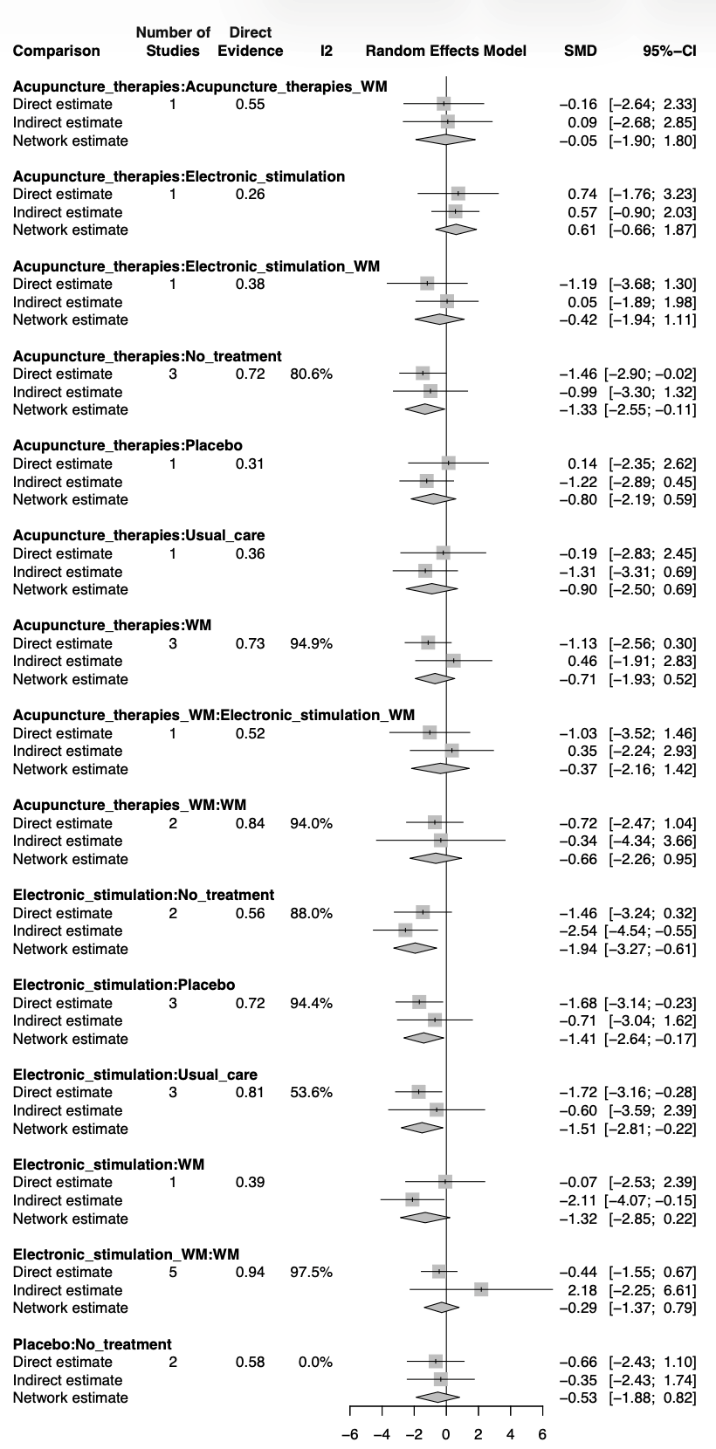 | 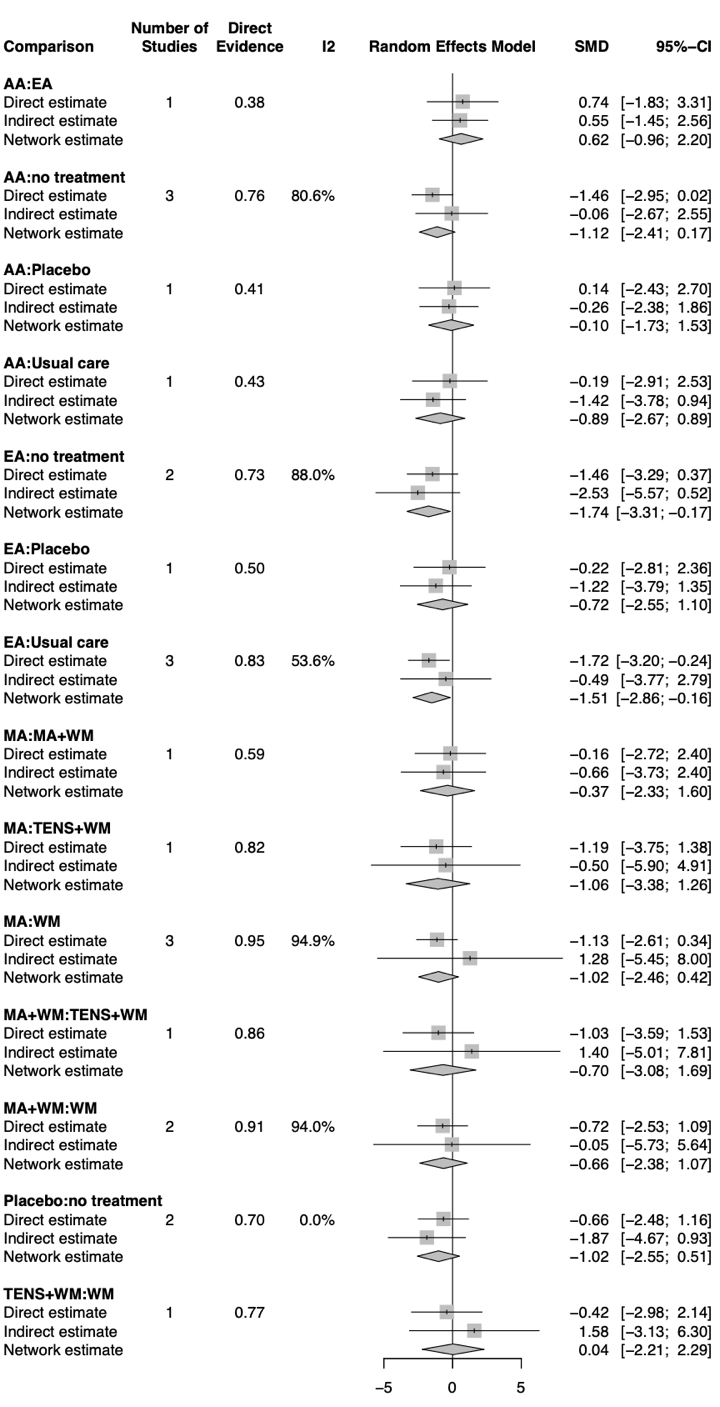 |

Supplementary Material 4. Pairwise meta-analysis in subgroup analysis

| **Outcome** |  |  | **Studies** | **Participants** | **MD/SMD [95% CI**] | ***I^2^*** | **Z** | **P** | **Model** |
| --- | --- | --- | --- | --- | --- | --- | --- | --- | --- |
| Anxiety | TEAS | Sham_TEAS | 3 | 301 | -2.02 [-3.50, -0.55] | *95* | 2.68 | 0.007 | Random |
|  | Sham_EA | No treatment | 3 | 161 | -0.41 [-1.10, 0.28] | *79* | 1.17 | 0.24 | Random |
|  | AA | No treatment | 2 | 102 | MD, -6.95 [-8.65, -5.24] | *45* | 10.85 | <0.0001 | Fixed |
|  | MA | No treatment | 2 | 150 | MD, -15.07 [-26.23, -3.92] | *99* | 2.65 | 0.0008 | Random |
|  | AA | Usual care | 2 | 88 | 0.27 [-0.15, 0.69] | *0* | 1.25 | 0.21 | Fixed |
|  | EA | Usual care | 2 | 148 | MD, -2.62 [-4.60, -0.64] | *73* | 2.59 | *0.010* | Random |
|  | EA | WM | 1 | 50 | MD, -4.58 [-7.76, -1.40] | *NA* | 2.82 | *0.005* | Random |
|  | MA | WM | 1 | 220 | MD, -3.64 [-6.99, -0.29] | *NA* | 2.13 | *0.03* | Random |
|  | TEAS | No treatment | 1 | 69 | MD, -5.31 [-16.87, 6.25] | *NA* | 0.90 | *0.37* | Random |
|  | MA+WM | WM | 1 | 60 | MD, -2.32 [-3.35, -1.29] | *NA* | 4.42 | <0.00001 | Random |
|  | MA | Sham_MA | 1 | 89 | MD, -7.80 [-11.49, -4.11] | *NA* | 4.14 | <0.0001 | Random |
|  | EA | AA | 1 | 52 | MD, -2.11 [-3.74, -0.48] | *NA* | 2.54 | 0.01 | Random |
|  | EA | No treatment | 2 | 91 | MD, -3.38 [-8.76, 1.99] | *92* | 1.23 | 0.22 | Random |
|  | MA+Usual care | Usual care | 1 | 60 | MD, -2.10 [-6.84, 2.64] | *NA* | 0.87 | 0.39 | Random |
|  | EA | Sham_EA | 1 | 41 | MD, -0.19 [-2.34, 1.96] | *NA* | 0.17 | 0.86 | Random |
|  | AA+ Usual care | Usual care | 1 | 62 | MD, -4.87 [-6.86, -2.88] | *NA* | 4.8 | <0.00001 | Random |
| Depression | TEAS | Sham_TEAS | 2 | 120 | SMD, -2.84 [-7.61, 1.93] | *98* | 1.17 | 0.24 | Random |
|  | Sham_EA | No treatment | 3 | 161 | MD, -0.46 [-0.77, -0.15] | *0* | 2.87 | 0.004 | Fixed |
|  | EA | Usual care | 2 | 148 | MD, -4.14 [-5.66, -2.62] | *21* | 5.34 | <0.0001 | Fixed |
|  | TEAS | No treatment | 1 | 69 | MD, -7.48 [-17.35, 2.39] | *NA* | 1.49 | 0.14 | Random |
|  | AA | No treatment | 2 | 102 | MD, -10.10 [-14.30, -5.89] | *88* | 4.71 | <0.00001 | Random |
|  | MA | No treatment | 1 | 90 | MD, -12.74 [-13.66, -11.82] | *NA* | 27.03 | <0.00001 | Random |
|  | MA | Sham_MA | 1 | 89 | MD, -4.80 [-7.59, -2.01] | *NA* | 3.37 | 0.0007 | Random |
|  | EA | AA | 1 | 52 | MD, -4.39 [-6.11, -2.67] | *NA* | 4.99 | <0.00001 | Random |
|  | EA | No treatment | 2 | 91 | MD, -5.02 [-10.74, 0.71] | *93* | 1.72 | 0.09 | Random |
|  | MA+Usual care | Usual care | 1 | 60 | MD, -1.30 [-7.35, 4.75] | *NA* | 0.42 | 0.67 | Random |
|  | AA | Usual care | 1 | 15 | MD, -1.90 [-4.77, 0.97] | *NA* | 1.30 | 0.19 | Random |
|  | EA | Sham_EA | 1 | 41 | MD, -1.09 [-3.46, 1.28] | *NA* | 0.90 | 0.37 | Random |
| Withdrawal symptoms | TEAS | Sham_TEAS | 2 | 118 | SMD, -2.38 [-3.78, -0.99] | *88* | 3.35 | 0.0008 | Random |
|  | EA+WM | WM | 4 | 338 | SMD, -0.46 [-2.25, 1.34] | *98* | 0.50 | 0.62 | Random |
|  | MA | WM | 3 | 341 | SMD, -1.18 [-2.42, 0.05] | *95* | 1.87 | 0.06 | Random |
|  | MA+WM | WM | 2 | 140 | MD, -7.17 [-21.54, 7.20] | *97* | 0.98 | 0.33 | Random |
|  | Sham_EA | No treatment | 2 | 111 | SMD, -0.65 [-1.05, -0.25] | *0* | 3.18 | 0.001 | Fixed |
|  | AA | No treatment | 3 | 252 | SMD, -1.38 [-2.05, -0.71] | *79* | 4.02 | <0.0001 | Random |
|  | EA | Usual care | 3 | 235 | MD, -4.78 [-5.47, -4.08] | *0* | 13.40 | <0.0001 | Fixed |
|  | EA | WM | 1 | 110 | MD, -0.36 [-2.32, 1.60] | *NA* | 0.36 | 0.72 | Random |
|  | MA+WM | MA | 1 | 60 | MD, 1.60 [0.28, 2.92] | *NA* | 2.38 | 0.03 | Random |
|  | MA | TEAS+WM | 1 | 60 | MD, -12.04 [-17.63, -6.45] | *NA* | 4.22 | <0.0001 | Random |
|  | TEAS+WM | MA | 1 | 60 | MD, -4.23 [-11.36, 2.90] | *NA* | 1.16 | 0.25 | Random |
|  | EA | AA | 1 | 52 | MD, -2.04 [-3.58, -0.50] | *NA* | 2.60 | 0.009 | Random |
|  | EA | No treatment | 2 | 91 | SMD, -1.05 [-1.49, -0.60] | *27* | 4.64 | <0.00001 | Fixed |
|  | AA | Usual care | 1 | 15 | MD, -0.70 [-4.54, 3.14] | *NA* | 0.36 | 0.72 | Random |
|  | EA | Sham_EA | 1 | 41 | MD, -0.61 [-2.13, 0.91] | *NA* | 0.79 | 0.43 | Random |
|  | AA+ Usual care | Usual care | 1 | 154 | SMD, -2.43 [-8.23, 3.38] | *NA* | 0.82 | 0.41 | Random |
|  | AA | Sham_AA | 1 | 60 | MD, 0.84 [-2.30, 3.98] | *NA* | 0.52 | 0.60 | Random |
|  | MA+WM | TEAS+WM | 1 | 60 | MD, -10.44 [-16.11, -4.77] | *NA* | 3.61 | 0.0003 | Random |

Abbreviation: AA: auricular acupuncture, MA: Manual acupuncture, EA: electro acupuncture, TEAS: transcutaneous electric acupoint stimulation, WM: Western medicine, WA: Warm acupuncture, MD: Mean difference, SMD: standardized mean difference

Supplementary Material 5. network evidence diagram of different outcome in subgroup analysis


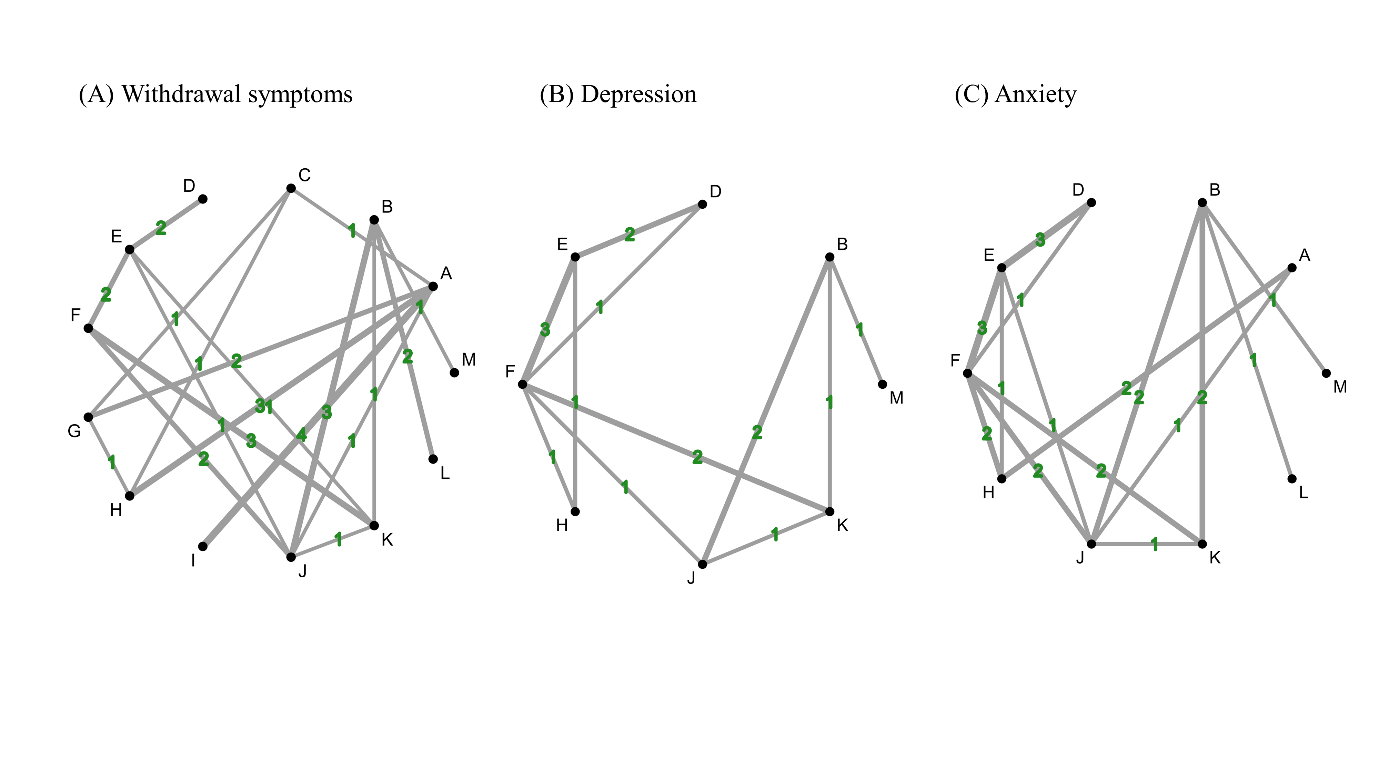


Abbreviation: A: Western medicine, B: Usual care, C: Transcutaneous electric acupoint stimulation + Western medicine, D: Transcutaneous electric acupoint stimulation, E: Placebo: F: No treatment, G: Manual acupuncture + Western medicine, H: Manual acupuncture, I: Electro acupuncture + Western medicine, J: Electro acupuncture, K: auricular acupuncture, L: auricular acupuncture + Usual care, M: Manual acupuncture + Usual care

Supplementary Material 6. Netleague table for withdrawal symptoms in subgroup analysis (SMD, 95% CI)

| TEAS |  |  |  |  |  |  |  |  |  |  |  |  |
| --- | --- | --- | --- | --- | --- | --- | --- | --- | --- | --- | --- | --- |
| -0.75  [-4.65; 3.16] | MA |  | -0.16  [-2.72; 2.40] |  |  | -1.19  [-3.75; 1.38] | -1.13  [-2.61; 0.34] |  |  | **-2.42**  **[-4.26;-0.58]** |  |  |
| -0.87  [-4.22; 2.48] | -0.13  [-3.84; 3.58] | AA+ Usual care |  |  |  |  |  |  |  |  |  |  |
| -1.11  [-5.13; 2.90] | -0.37  [-2.33; 1.60] | -0.24  [-4.07; 3.59] | MA+WM |  |  | -1.03  [-3.59; 1.53] | -0.72  [-2.53; 1.09] |  |  |  | **-2.34**  **[-4.19;-0.49]** |  |
| -1.32  [-5.16; 2.53] | -0.57  [-2.50; 1.36] | -0.44  [-4.09; 3.20] | -0.21  [-2.35; 1.94] | EA+WM |  |  | -0.45  [-1.73; 0.83] |  |  |  |  |  |
| -1.70  [-4.29; 0.89] | -0.95  [-3.87; 1.97] | -0.83  [-3.11; 1.46] | -0.59  [-3.66; 2.48] | -0.38  [-3.23; 2.46] | EA |  | -0.07  [-2.61; 2.47] |  | -0.74  [-3.31; 1.83] | -0.22  [-2.81; 2.36] | **-1.72**  **[-3.20;-0.24]** | -1.46  [-3.29; 0.37] |
| -1.81  [-6.08; 2.46] | -1.06  [-3.38; 1.26] | -0.93  [-5.03; 3.16] | -0.70  [-3.08; 1.69] | -0.49  [-3.08; 2.10] | -0.11  [-3.50; 3.29] | TEAS+WM | -0.42  [-2.98; 2.14] |  |  |  |  |  |
| -1.77  [-5.40; 1.86] | -1.02  [-2.46; 0.42] | -0.90  [-4.31; 2.52] | -0.66  [-2.38; 1.07] | -0.45  [-1.73; 0.83] | -0.07  [-2.61; 2.47] | 0.04  [-2.21; 2.29] | WM |  |  |  |  |  |
| -2.18  [-5.98; 1.62] | -1.43  [-5.55; 2.69] | -1.30  [-4.47; 1.86] | -1.07  [-5.29; 3.16] | -0.86  [-4.92; 3.20] | -0.48  [-3.38; 2.43] | -0.37  [-4.84; 4.10] | -0.41  [-4.27; 3.45] | WA |  |  | -1.03  [-3.61; 1.54] |  |
| -2.32  [-4.78; 0.14] | -1.58  [-4.90; 1.74] | -1.45  [-4.02; 1.12] | -1.21  [-4.66; 2.24] | -1.00  [-4.26; 2.25] | -0.62  [-2.20; 0.96] | -0.52  [-4.26; 3.23] | -0.55  [-3.55; 2.44] | -0.14  [-3.27; 2.98] | AA | 0.14  [-2.43; 2.70] | -0.19  [-2.91; 2.53] | -1.46  [-2.95; 0.02] |
| **-2.42**  **[-4.26; -0.58]** | -1.68  [-5.12; 1.77] | -1.55  [-4.35; 1.25] | -1.31  [-4.88; 2.26] | -1.10  [-4.48; 2.27] | -0.72  [-2.55; 1.10] | -0.61  [-4.47; 3.24] | -0.65  [-3.78; 2.47] | -0.24  [-3.57; 3.08] | -0.10  [-1.73; 1.53] | Placebo |  | -0.66  [-2.48;  1.16] |
| **-3.21**  **[-6.01; -0.42]** | -2.47  [-5.68; 0.75] | **-2.34**  **[-4.19; -0.49]** | -2.10  [-5.45; 1.25] | -1.89  [-5.04; 1.25] | **-1.51**  **[-2.86; -0.16]** | -1.41  [-5.06; 2.25] | -1.44  [-4.32; 1.43] | -1.03  [-3.61; 1.54] | -0.89  [-2.67; 0.89] | -0.79  [-2.89; 1.31] | Usual care |  |
| **-3.44**  **[-5.83; -1.05]** | -2.70  [-6.01; 0.62] | -2.57  [-5.20; 0.06] | -2.33  [-5.78; 1.12] | -2.13  [-5.37; 1.12] | **-1.74**  **[-3.31; -0.17]** | -1.64  [-5.37; 2.10] | -1.67  [-4.66; 1.31] | -1.26  [-4.45; 1.92] | -1.12  [-2.41; 0.17] | -1.02  [-2.55; 0.51] | -0.23  [-2.11; 1.65] | No treatment |

*The black bold result means it had statistically difference between the interventions and controls. In the case of effect, when the standard mean difference is < 0, it means the effectiveness of the column treatment is better, otherwise, it not.

Supplementary Material 7. Sensitivity analysis for withdrawal symptoms in subgroup analysis

| Not available  (Excluded Zhao 1997) | 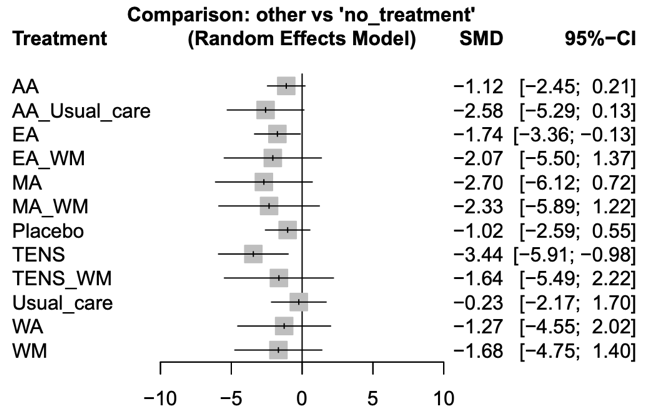  (Excluded Zeng (2003) |
| --- | --- |
| 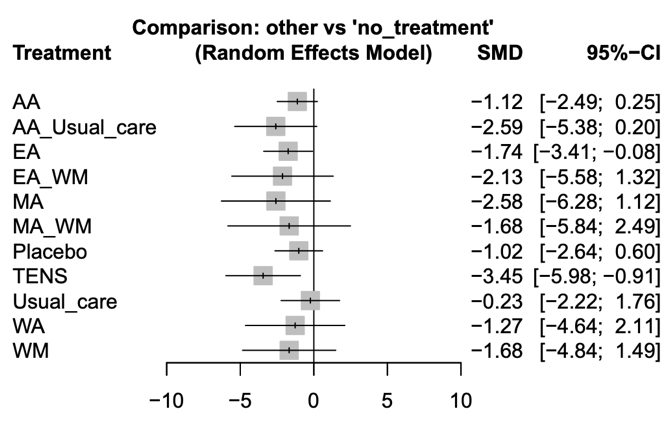  (Excluded Wu 2003) | 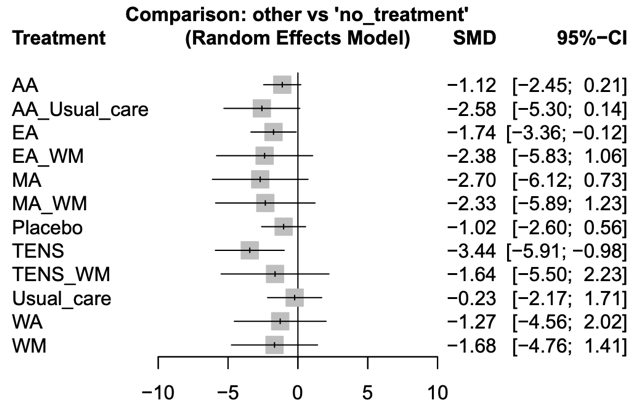  (Exclued Zhang 2004) |
| 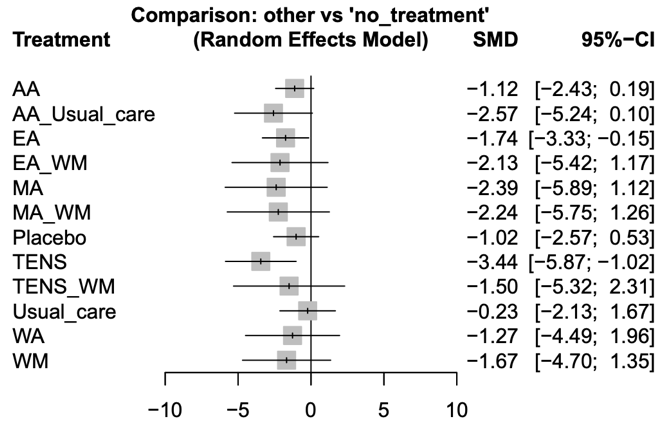  (Exclued Rong 2005) | 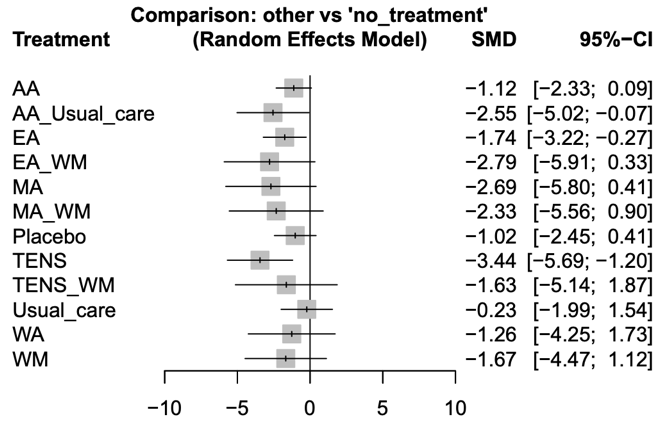  (Exclued Zhang 2005) |
| 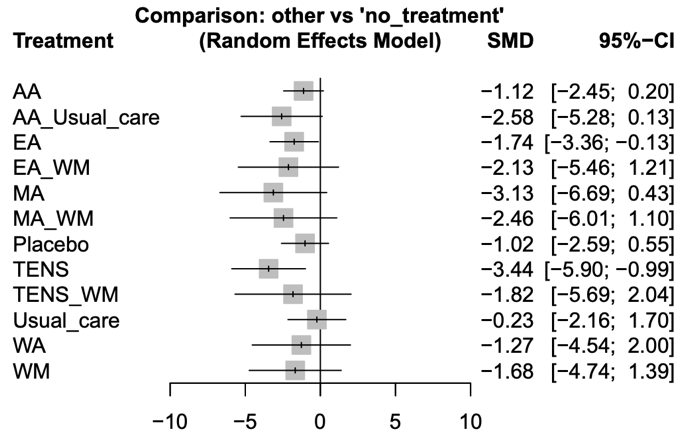  (Exclued Wen 2005) | 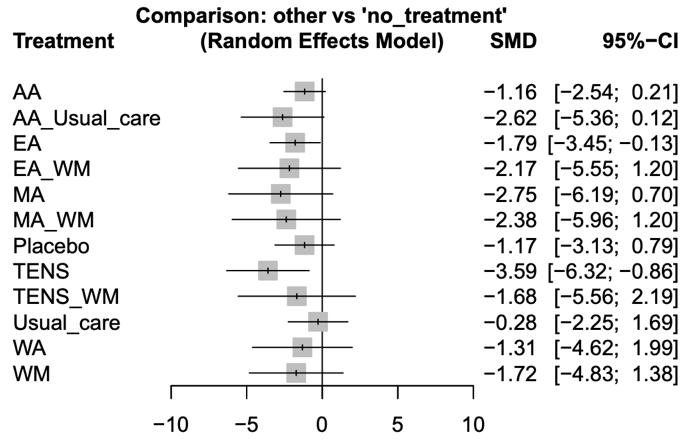  (Exclued Mu 2005) |
| 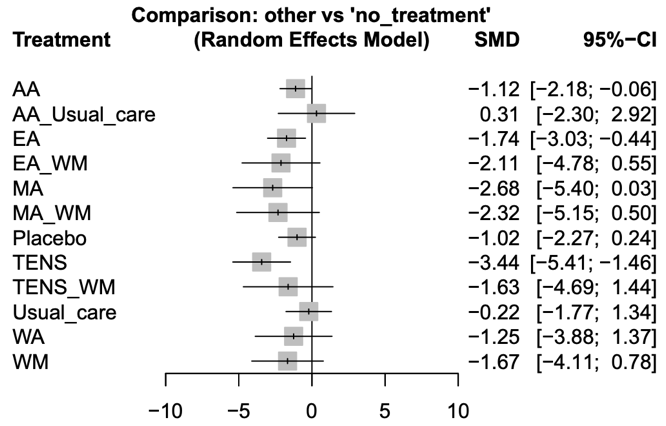  (Exclued Li 2006a) | 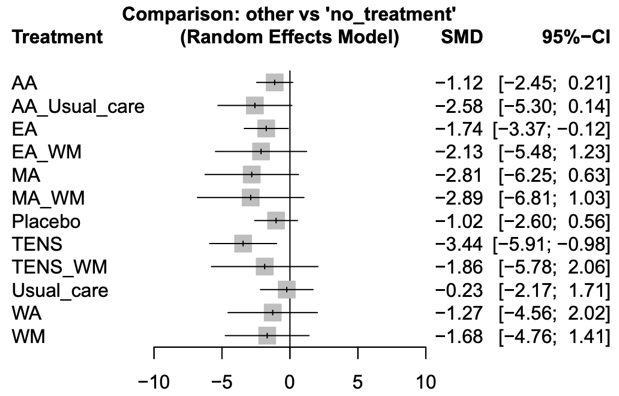  (Exclued Li 2006b) |
| 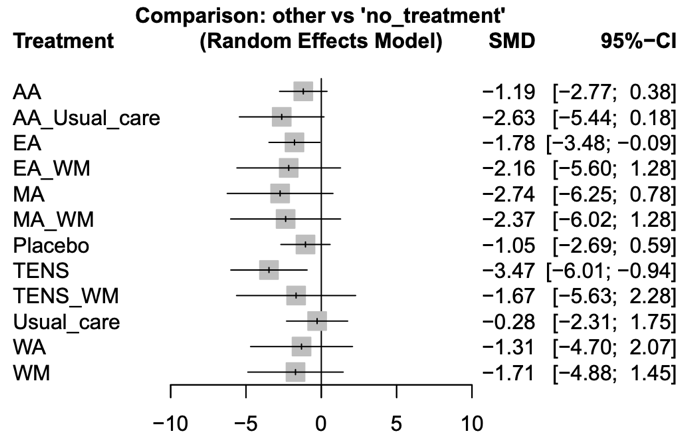  (Exclued Song 2006) | 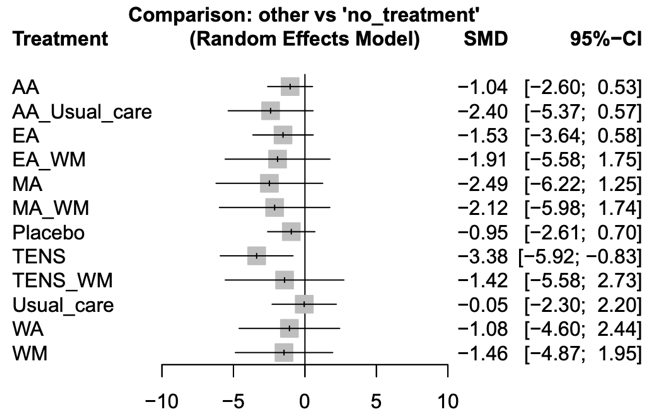  (Exclued Liang 2014) |
| 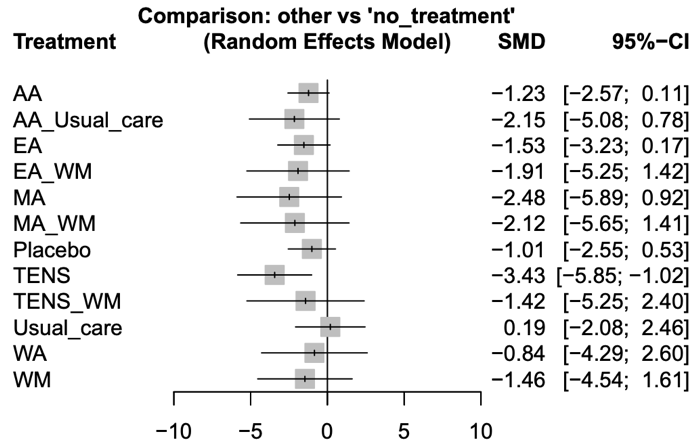  (Exclued Jackson 2016) | 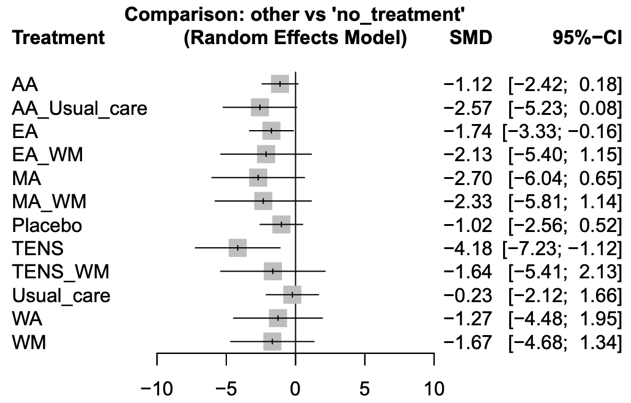  (Exclued Luo 2017) |
| 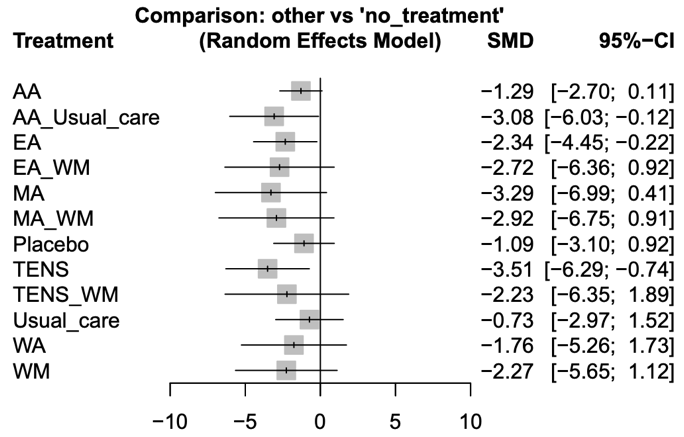  (Exclued Wang 2020) | 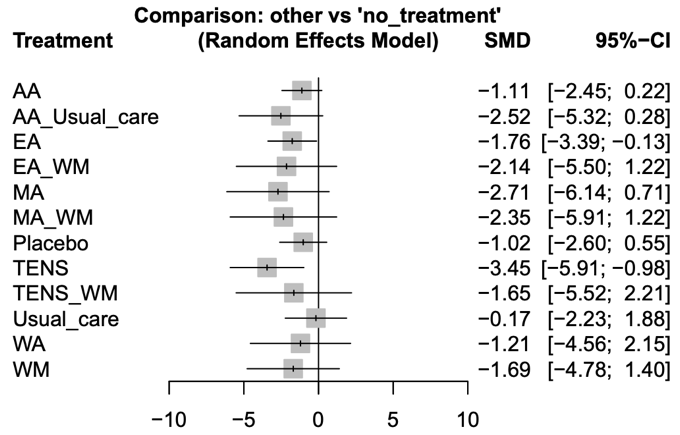  (Exclued Gu 2023) |
| 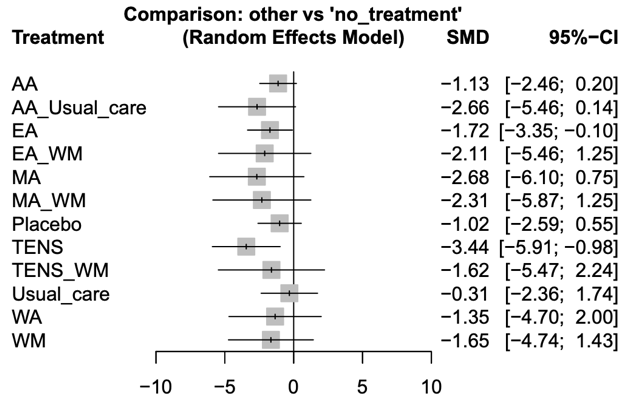  (Exclued Kong 2021) | 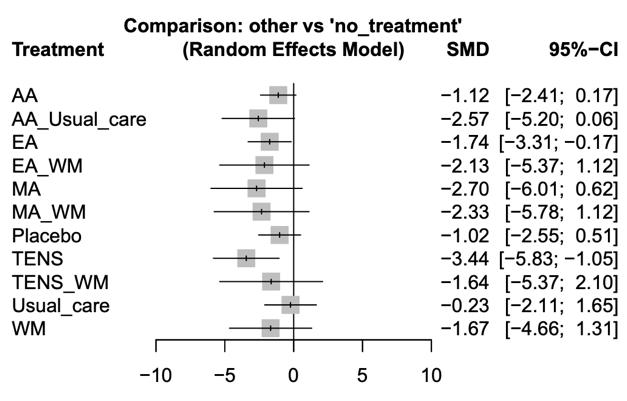  (Exclued Gao 2021) |
| 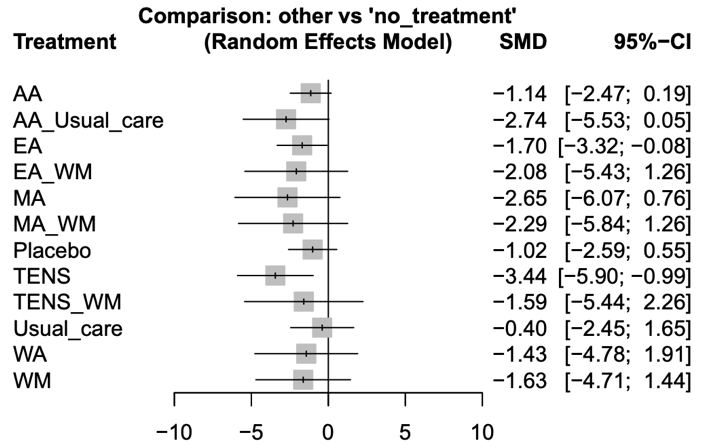  (Exclued Yu 2023) | 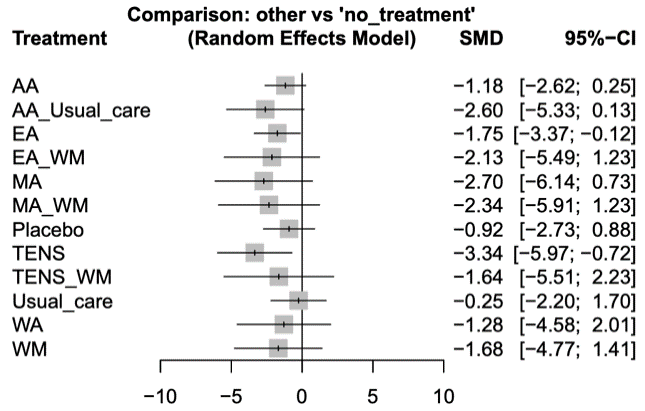  (Exclued Wei 2011) |
| 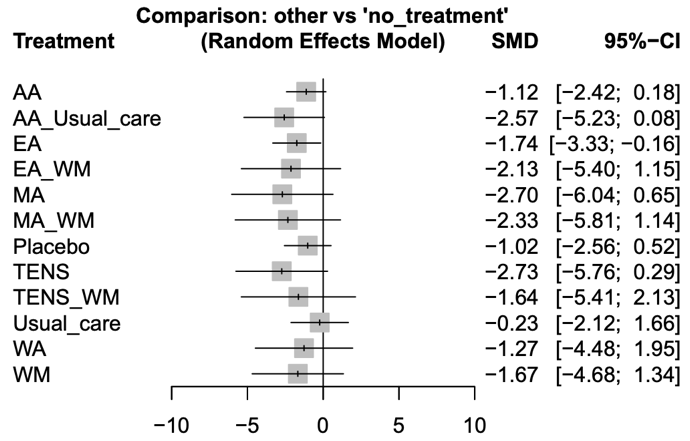  (Exclued Zhu 2008) | 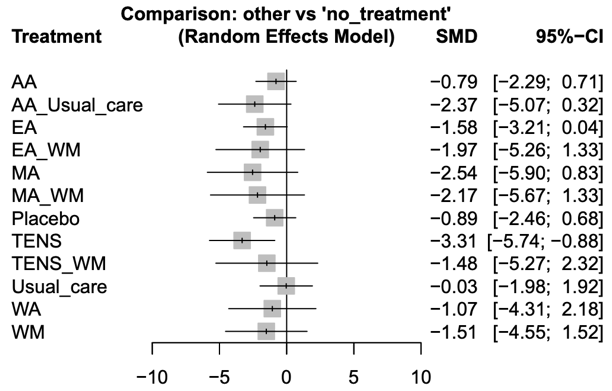  (Exclued Xu 2016) |
| 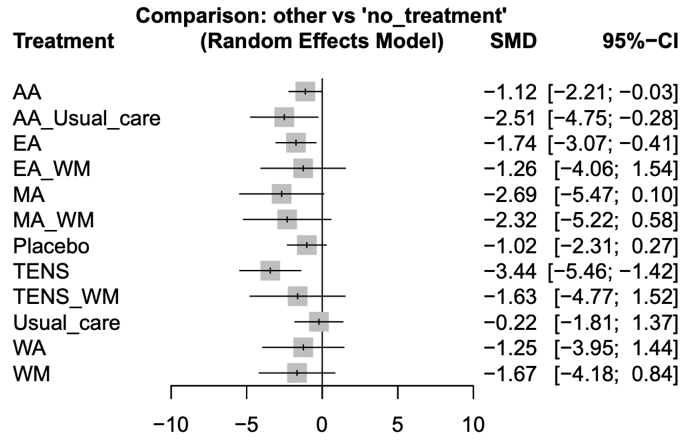  (Exclued Chen 2024) | 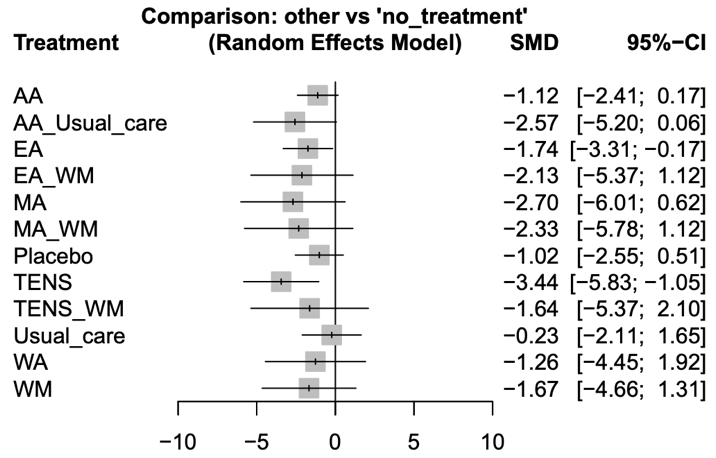  (Exclued Sun 2021) |

Supplementary Material 8. Meta-regression of withdrawal symptoms


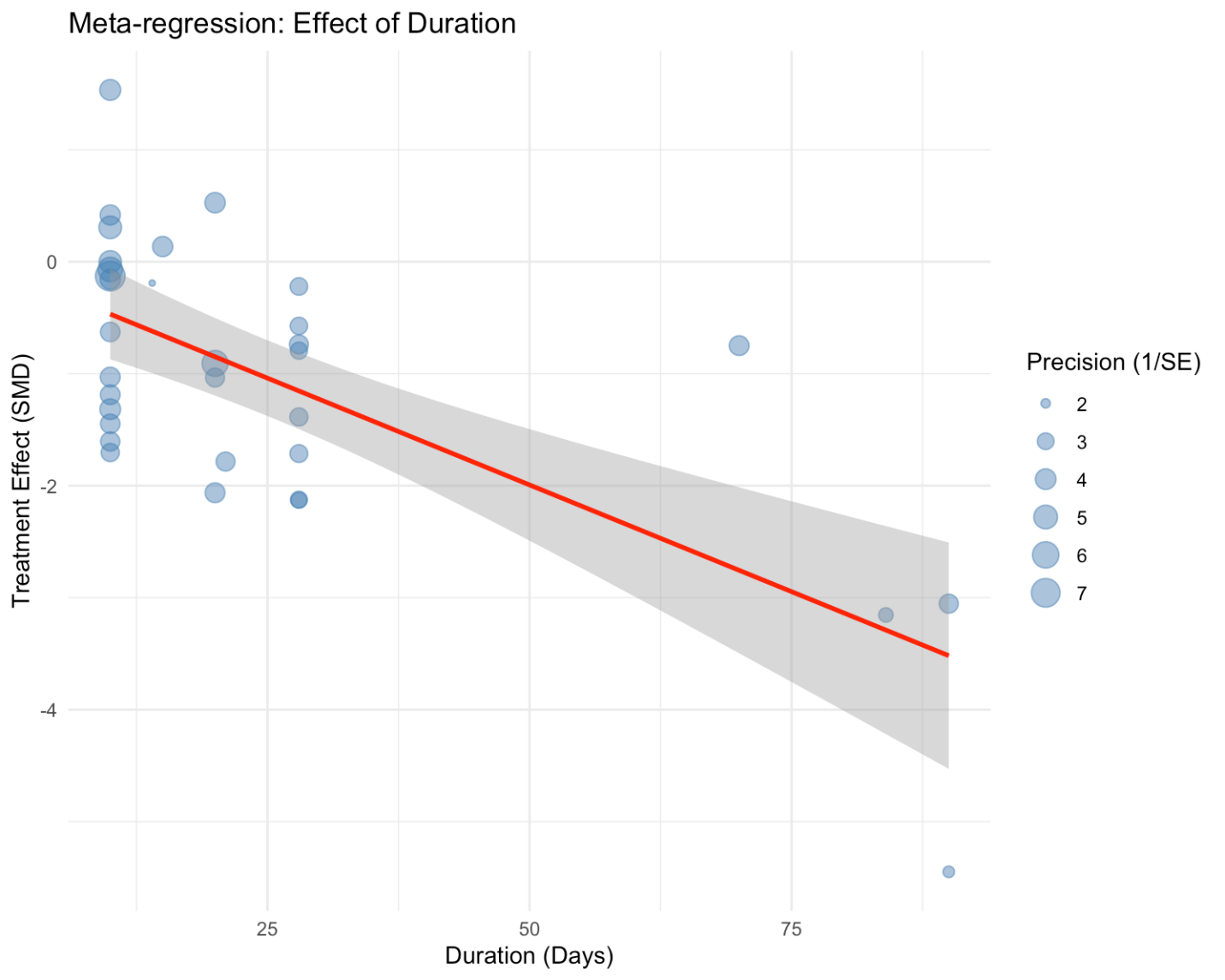


Supplementary Material 9. Forest plot of Depression

(A) Acupuncture vs Placebo


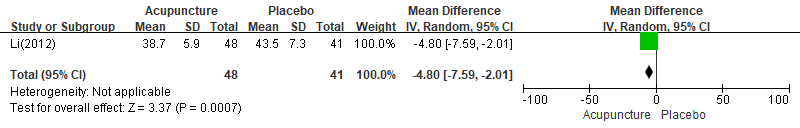


(B) Acupuncture vs No treatment


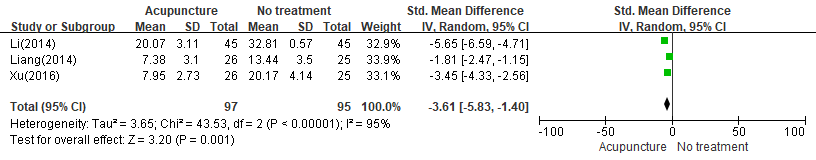


(C) Electronic stimulation vs No treatment


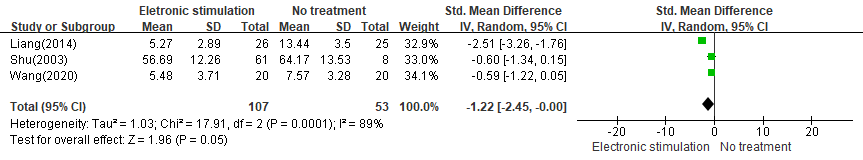


(D) Electronic stimulation vs Usual care


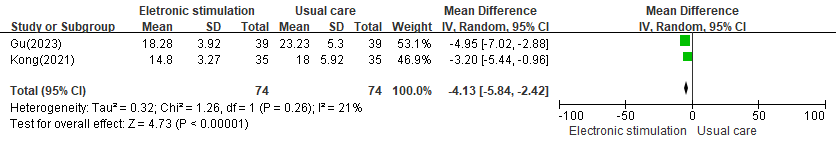


(E) Electronic stimulation vs Acupuncture


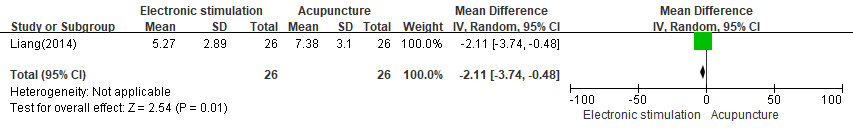


(F) Placebo vs No treatment


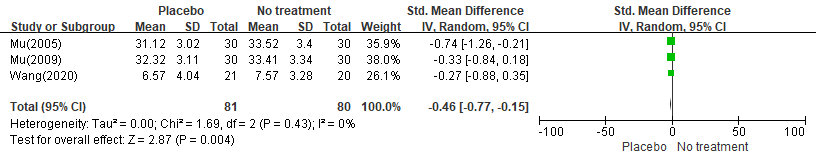


(G) Acupuncture vs Usual care


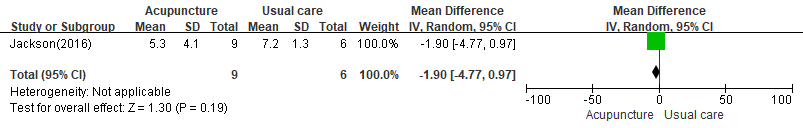


(H) Acupuncture + Usual care vs Usual care


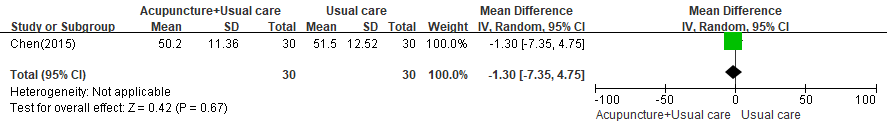


(I) Electronic stimulation vs Placebo


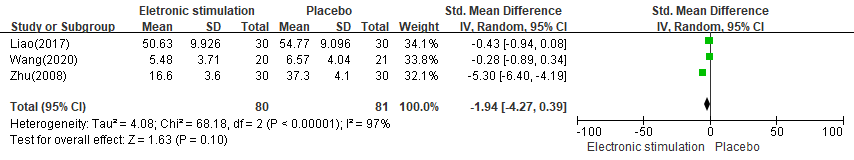


Supplementary Material 10. Netleague table for Depression (SMD, 95%CI)

| Electronic stimulation | -1.26 [-3.92; 1.40] |  | -0.87 [-2.73; 1.00] | **-2.75 [-4.68; -0.82]** | **-2.06 [-3.97; -0.15]** |
| --- | --- | --- | --- | --- | --- |
| -0.00 [-1.53; 1.53] | Acupuncture therapies |  | -0.57 [-3.37; 2.23] | -0.73 [-3.36; 1.90] | **-3.79 [-5.36; -2.22]** |
| -0.67 [-3.77; 2.43] | -0.66 [-3.91; 2.58] | Acupuncture therapies + Usual care | -0.11 [-2.75; 2.54] |  |  |
| -0.78 [-2.40; 0.85] | -0.77 [-2.65; 1.11] | -0.11 [-2.75; 2.54] | Usual care |  |  |
| **-2.28 [-3.69; -0.87]** | **-2.28 [-3.77; -0.78]** | -1.61 [-4.92; 1.70] | -1.50 [-3.50; 0.49] | Placebo | -0.45 [-1.98; 1.08] |
| **-2.96 [-4.36; -1.56]** | **-2.96 [-4.28; -1.63]** | -2.29 [-5.58; 0.99] | **-2.18 [-4.13; -0.23]** | -0.68 [-1.91; 0.55] | No treatment |

*The black bold result means it had statistically difference between the interventions and controls. In the case of effect, when the standard mean difference is < 0, it means the effectiveness of the column treatment is better, otherwise, it not.

Supplementary Material 11. Forest plot of the node-splitting analysis for inconsistency assessment (Depression)

| (A) Primary analysis | (B) Subgroup analysis |
| --- | --- |
| 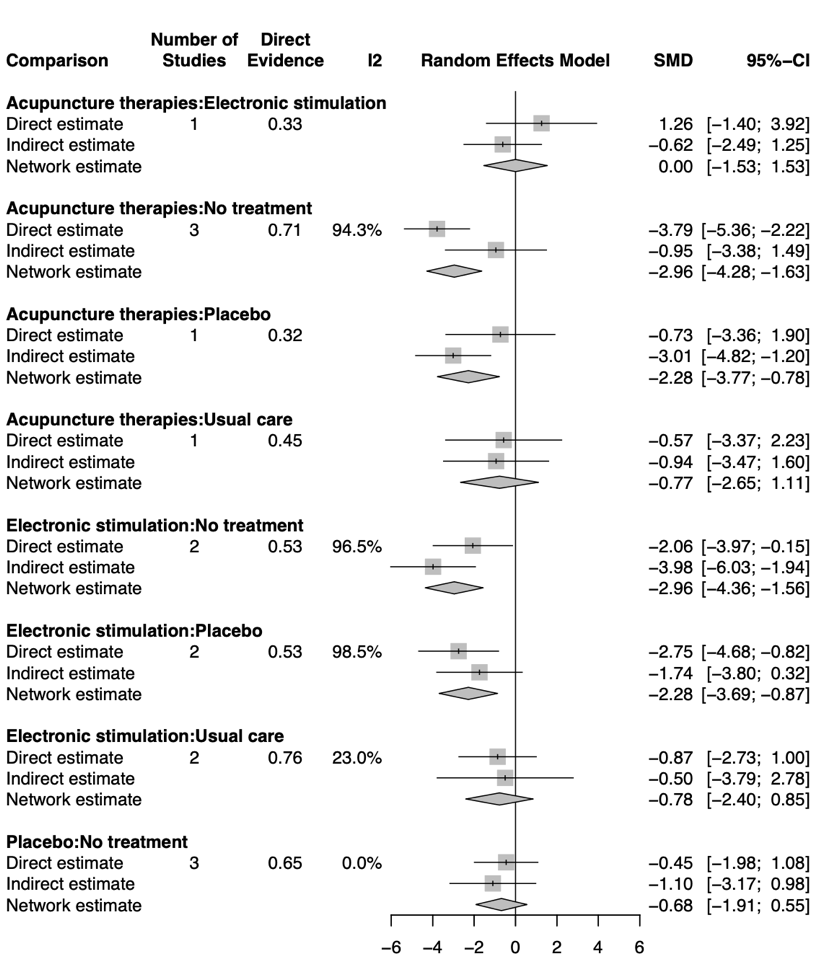 | 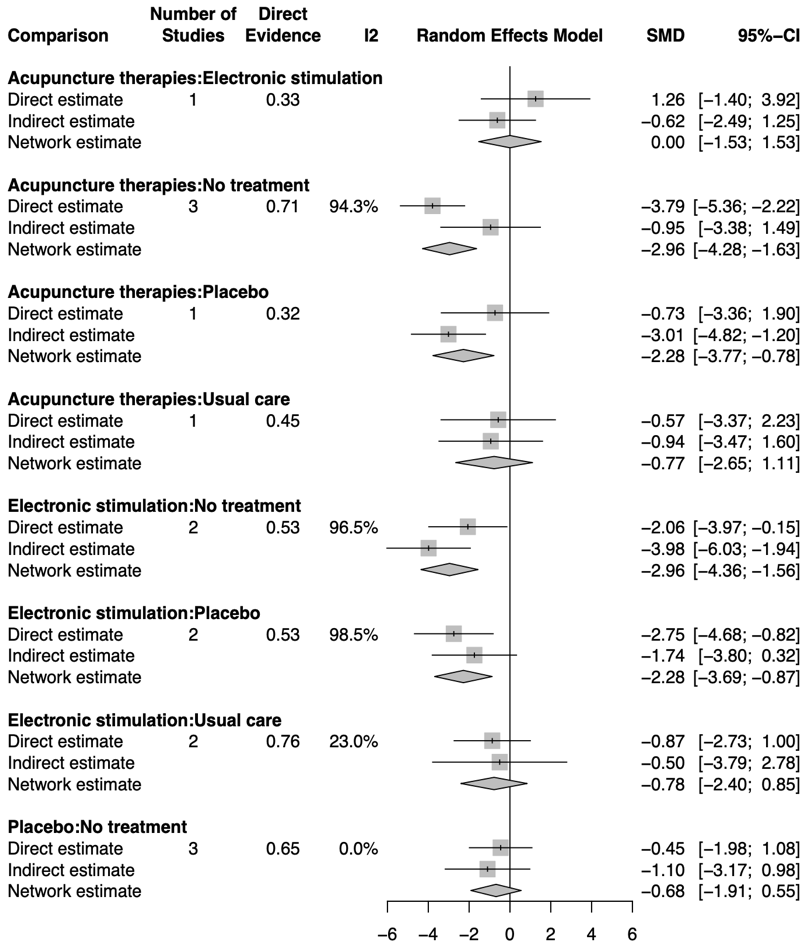 |

Supplementary Material 12. Netleague table for Depression in subgroup analysis (SMD, 95%CI)

| EA |  |  |  | -0.87 [-2.80; 1.07] |  |  | **-3.53 [-6.34; -0.73]** |
| --- | --- | --- | --- | --- | --- | --- | --- |
| -0.26 [-3.42; 2.90] | MA | -1.26 [-4.01; 1.50] |  | . |  | -0.73 [-3.46; 2.00] | **-5.70 [-8.55; -2.85]** |
| -0.73 [-2.81; 1.35] | -0.47 [-3.32; 2.38] | AA |  | -0.57 [-3.47; 2.32] |  |  | **-2.87 [-4.85; -0.89]** |
| -0.89 [-4.13; 2.35] | -0.63 [-4.94; 3.68] | -0.16 [-3.65; 3.32] | MA + Usual care | -0.11 [-2.85; 2.63] |  |  |  |
| -1.00 [-2.73; 0.73] | -0.74 [-4.07; 2.59] | -0.27 [-2.43; 1.88] | -0.11 [-2.85; 2.63] | Usual care |  |  |  |
| -1.25 [-4.26; 1.76] | -0.99 [-3.55; 1.57] | -0.53 [-3.20; 2.15] | -0.36 [-4.56; 3.84] | -0.25 [-3.44; 2.93] | TEAS | **-2.76 [-4.75; -0.76]** | -0.60 [-3.40; 2.19] |
| **-3.09 [-5.82; -0.36]** | **-2.83 [-4.90; -0.75]** | -2.36 [-4.72; 0.00] | -2.20 [-6.21; 1.81] | -2.09 [-5.01; 0.83] | **-1.84 [-3.52; -0.15]** | Placebo | -0.45 [-2.04; 1.14] |
| **-3.67 [-6.04; -1.30]** | **-3.41 [-5.50; -1.31]** | **-2.94 [-4.87; -1.01]** | -2.78 [-6.55; 0.99] | **-2.67 [-5.26; -0.08]** | **-2.42 [-4.27; -0.56]** | -0.58 [-1.93; 0.77] | No treatment |

*The black bold result means it had statistically difference between the interventions and controls. In the case of effect, when the standard mean difference is < 0, it means the effectiveness of the column treatment is better, otherwise, it not.

Abbreviation: AA: auricular acupuncture, MA: Manual acupuncture, EA: electro acupuncture, TEAS: transcutaneous electric acupoint stimulation

Supplementary Material 13. Sensitivity analysis for Depression in the subgroup analysis

| 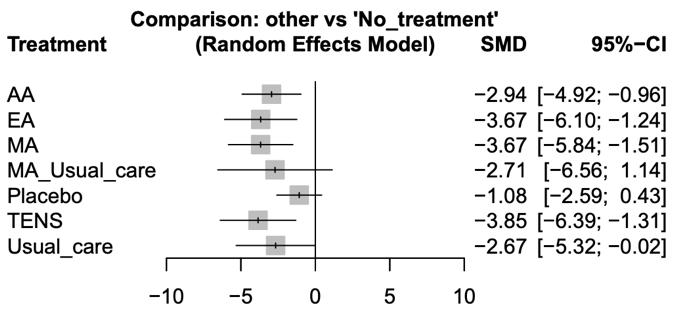  (Excluded Shu 2003) | 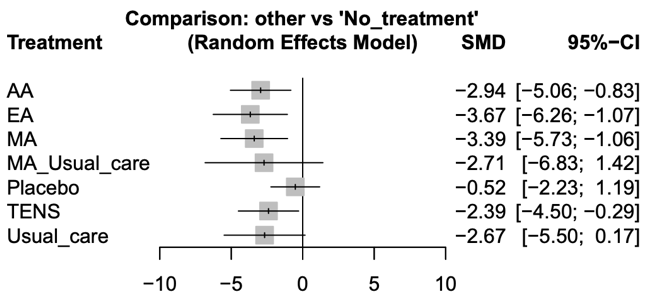  (Excluded Mu 2005) |
| --- | --- |
| 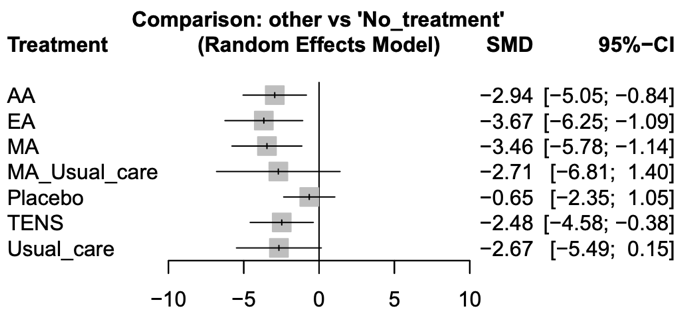  (Exclued Mu 2009) | 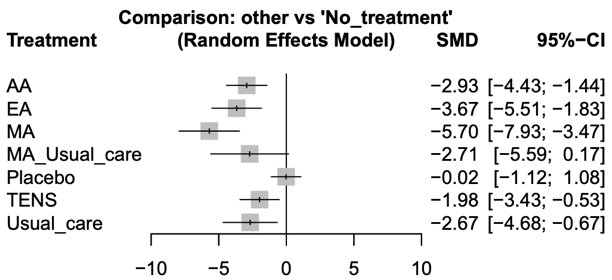  (Excluded Li 2012) |
| 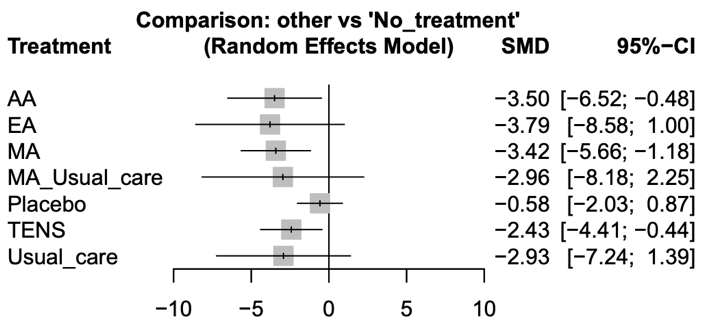  (Exclued Liang 2014) | 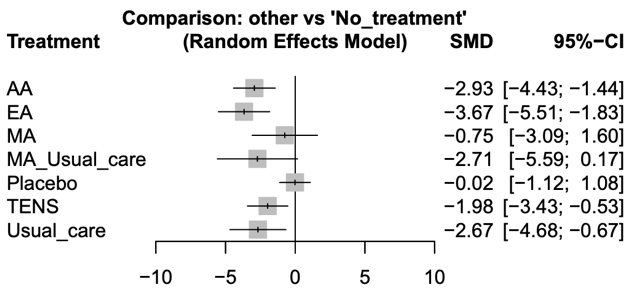  (Exclued Li 2014) |
| 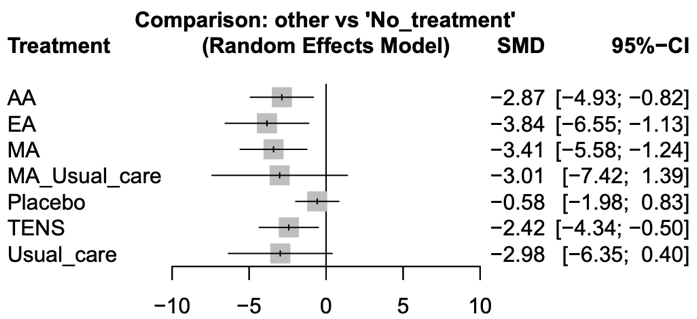  (Exclued Jackson 2016) | 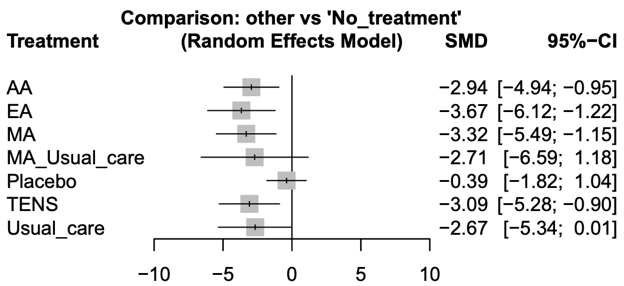  (Exclued Liao 2017) |
| 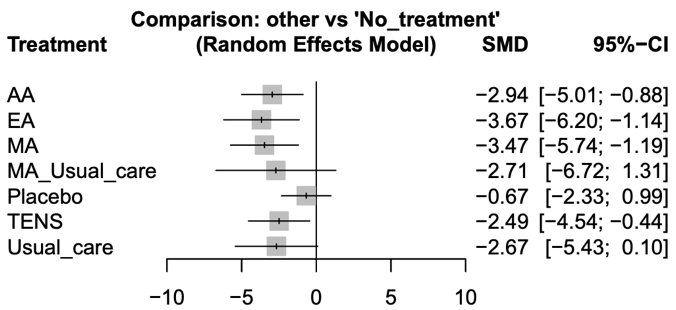  (Exclued Wang 2020) | 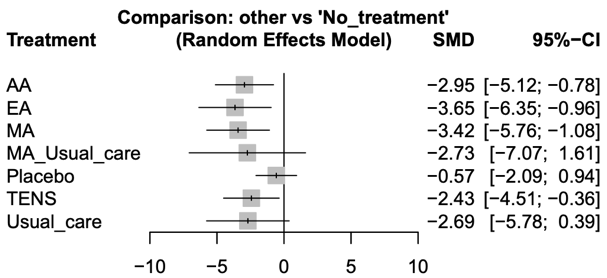  (Exclued Gu 2023) |
| 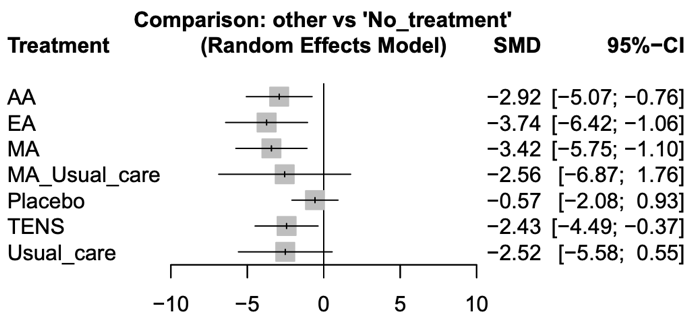  (Exclued Kong 2021) | 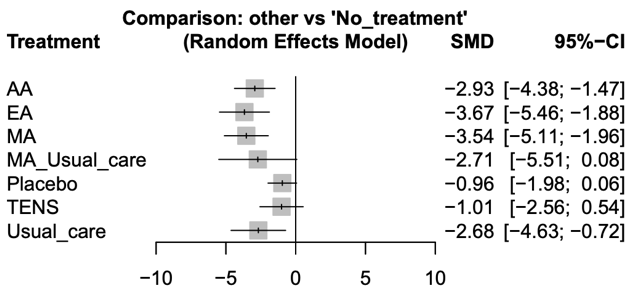  (Exclued Zhu 2008) |
| 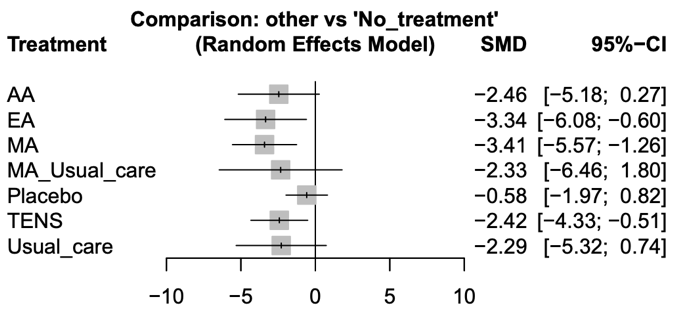  (Exclued Xu 2016) | 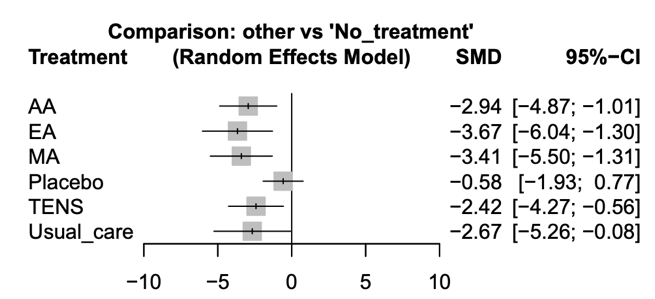  (Exclued Chen 2015) |

Supplementary Material 14. Meta-regression of depression


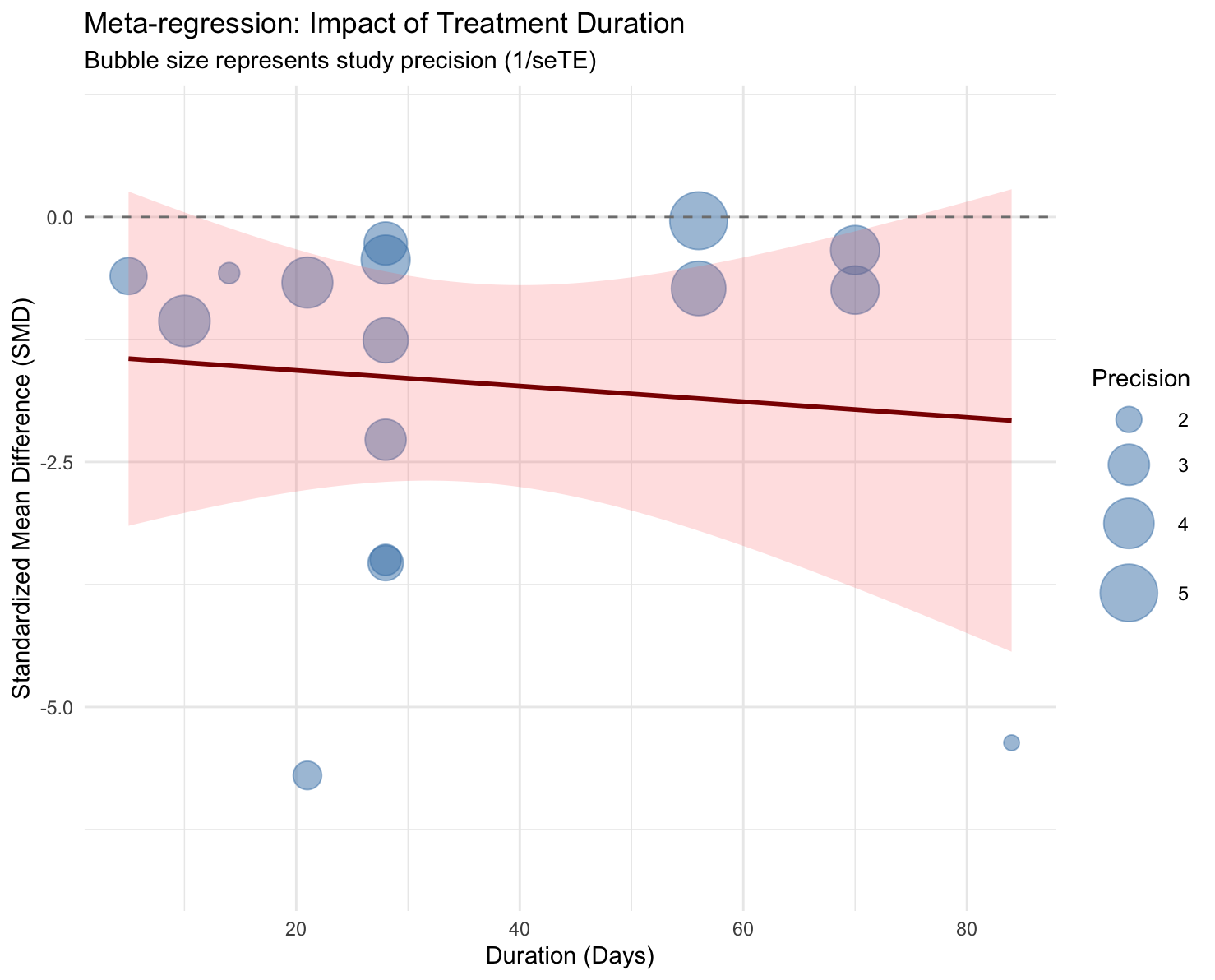


Supplementary Material 15. Subgroup analysis based on the type of drug and treatment duration (Depression)


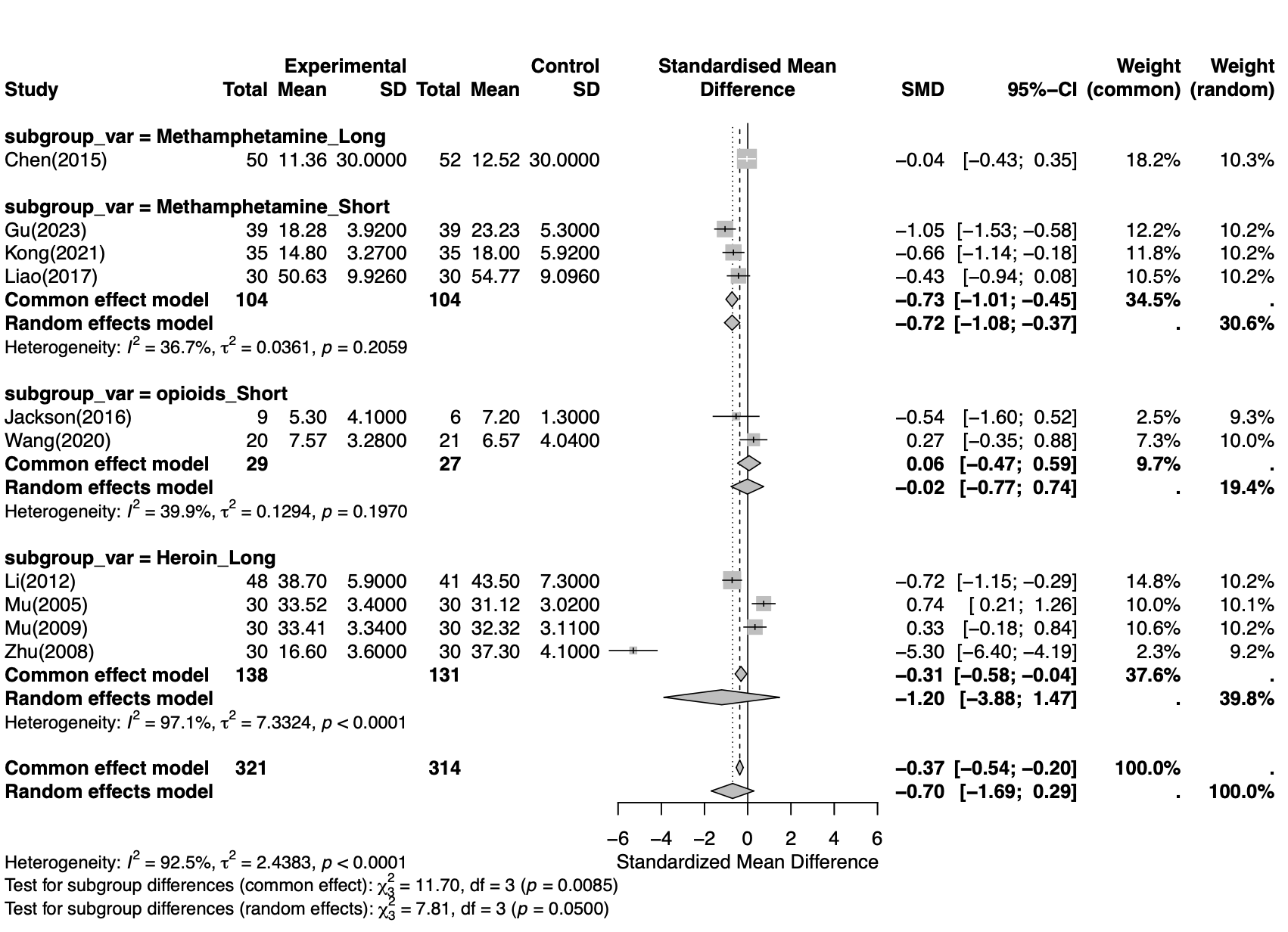


Supplementary Material 16. Forest plot of Anxiety

(A) Acupuncture vs No treatment


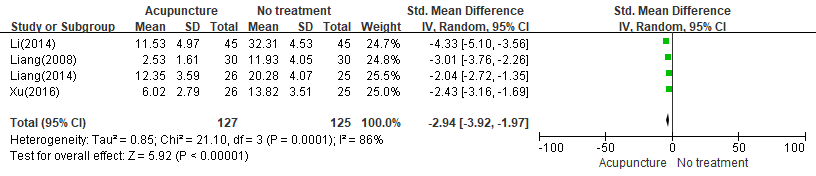


(B) Acupuncture vs Placebo


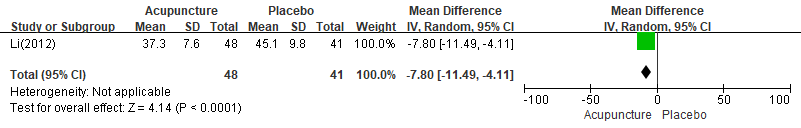


(C) Acupuncture + WM vs WM


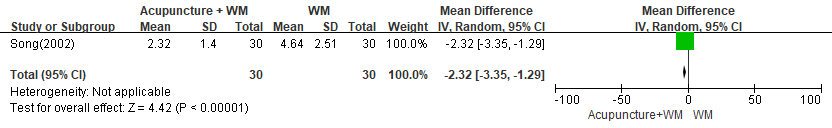


(D) Electronic stimulation vs WM


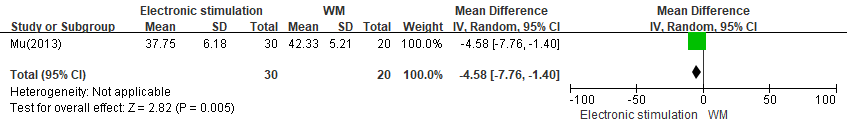


(E) Electronic stimulation + WM vs WM


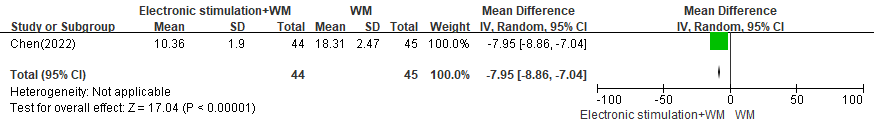


(F) Electronic stimulation vs Placebo


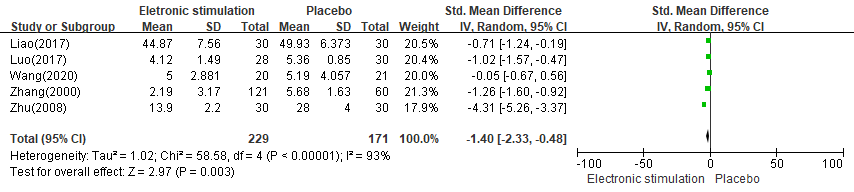


(G) Electronic stimulation vs Usual care


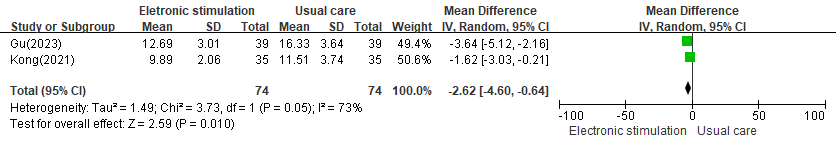


(H) Electronic stimulation vs Acupuncture


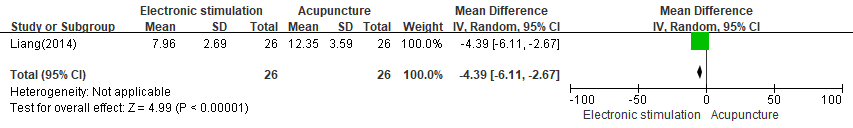


(I) Acupuncture+Usual care vs Usual care


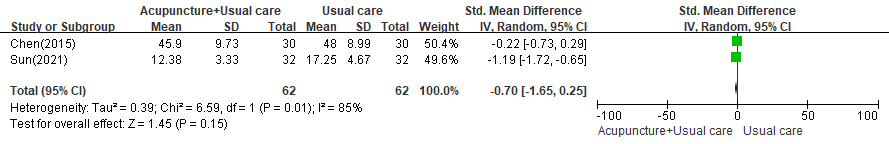


(J) Acupuncture vs Usual care


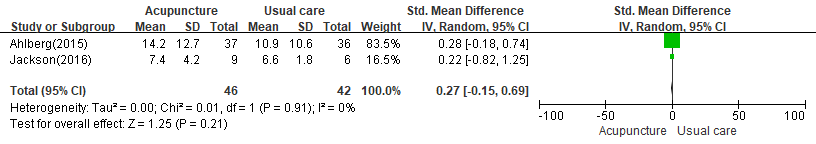


(K) Acupuncture vs WM


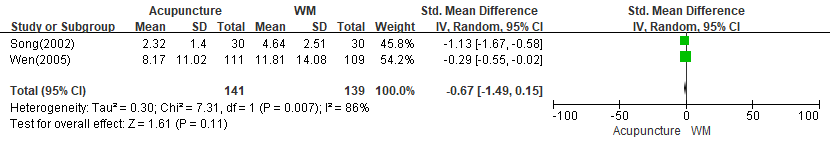


(L) Electronic stimulation vs No treatment


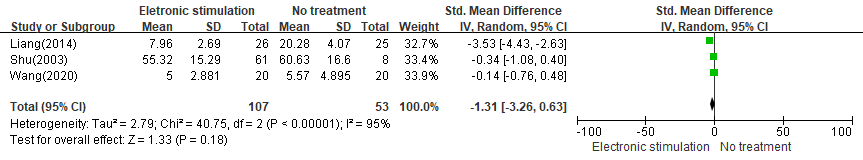


(M) Placebo vs No treatment


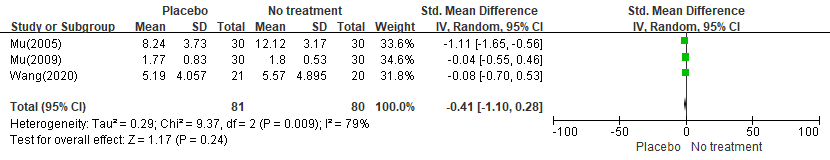


Abbreviation: WM, Western medicine

Supplementary Material 17. Netleague table for Anxiety (SMD, 95%CI)

| Acupuncture therapies + Usual care |  |  | -0.71 [-2.05; 0.62] |  |  |  |
| --- | --- | --- | --- | --- | --- | --- |
| -0.31 [-2.02; 1.40] | Electronic stimulation | 0.67 [-1.23; 2.56] | 0.26 [-1.14; 1.65] | -0.70 [-2.02; 0.62] | -0.90 [-2.77; 0.97] | **-2.94 [-3.91; -1.96]** |
| -0.49 [-2.19; 1.20] | -0.18 [-1.06; 0.69] | Acupuncture therapies | -0.81 [-2.14; 0.51] | -0.79 [-2.70; 1.12] | **-1.51 [-2.47; -0.55]** | -1.02 [-2.13; 0.10] |
| -0.71 [-2.05; 0.62] | -0.40 [-1.47; 0.67] | -0.22 [-1.26; 0.83] | Usual care |  |  |  |
| -1.10 [-3.08; 0.88] | -0.79 [-1.91; 0.33] | -0.60 [-1.84; 0.63] | -0.39 [-1.85; 1.07] | WM |  |  |
| **-1.98 [-3.79; -0.17]** | **-1.67 [-2.62; -0.72]** | **-1.49 [-2.29; -0.69]** | **-1.27 [-2.50; -0.05]** | -0.88 [-2.24; 0.47] | Placebo | -0.42 [-1.52; 0.68] |
| **-2.54 [-4.33; -0.75]** | **-2.23 [-3.04; -1.41]** | **-2.04 [-2.88; -1.21]** | **-1.83 [-3.02; -0.64]** | **-1.44 [-2.74; -0.14]** | -0.56 [-1.40; 0.29] | No treatment |

*The black bold result means it had statistically difference between the interventions and controls. In the case of effect, when the standard mean difference is < 0, it means the effectiveness of the column treatment is better, otherwise, it not.

Supplementary Material 18. Forest plot of the node-splitting analysis for inconsistency assessment (Anxiety)

| (A) Primary analysis | (B) Subgroup analysis |
| --- | --- |
| 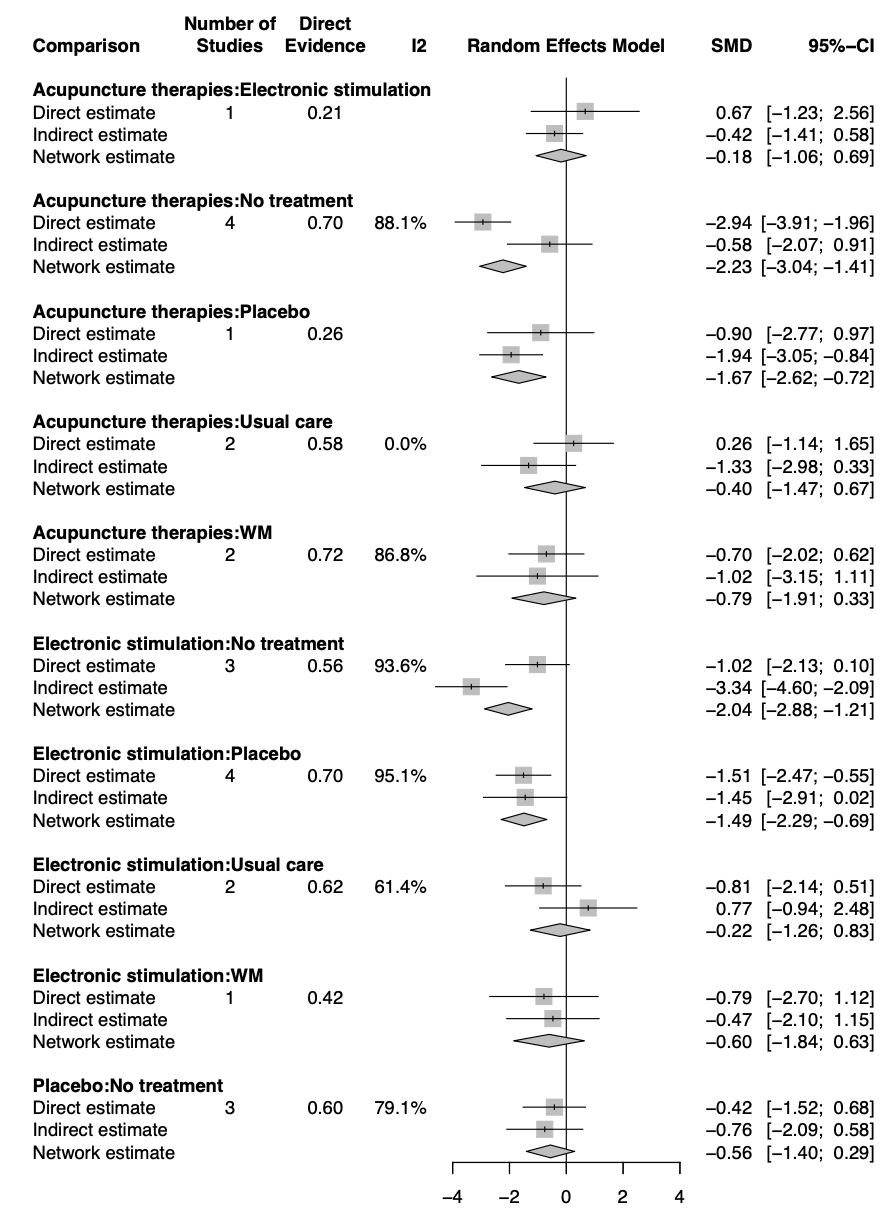 | 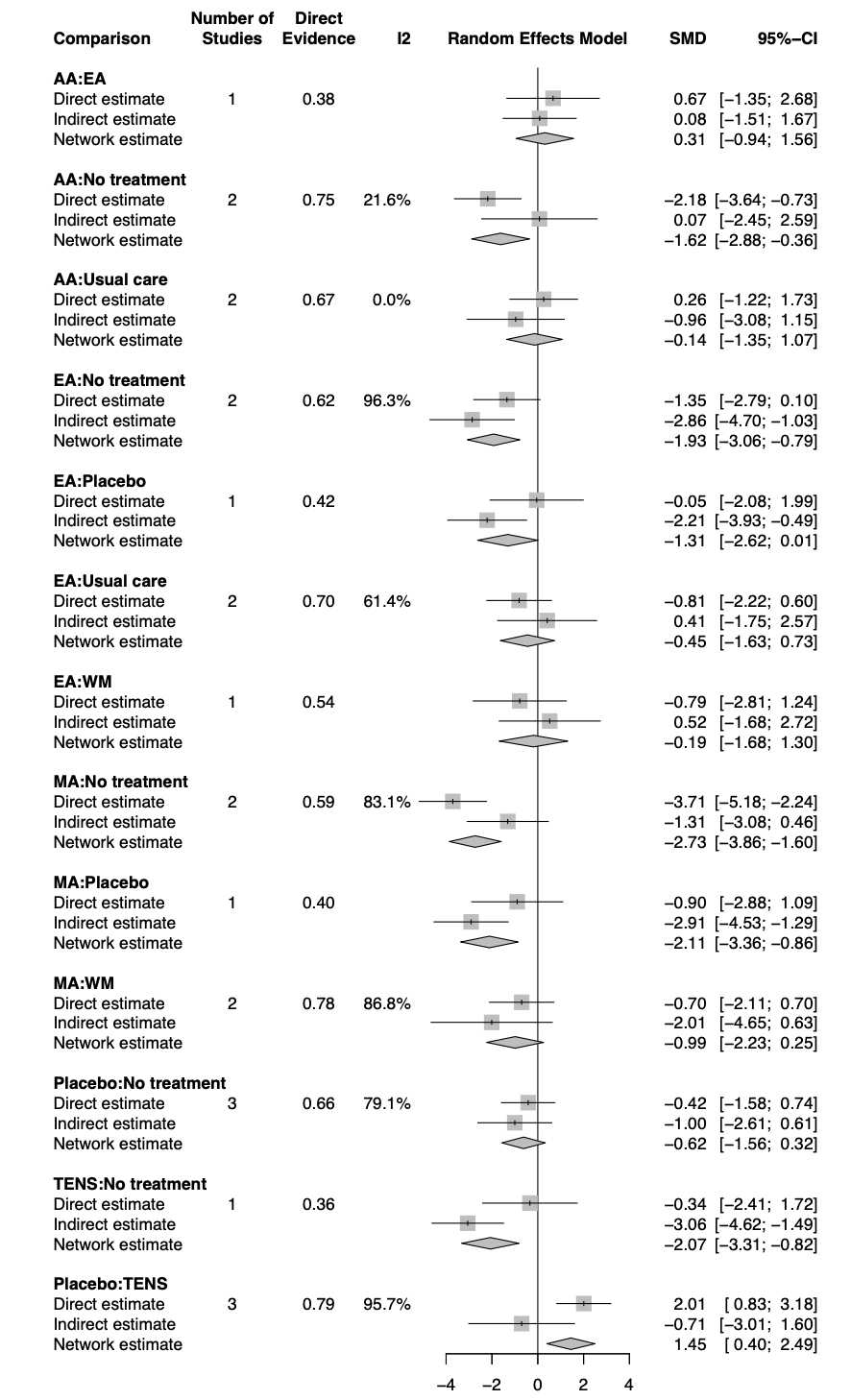 |

Supplementary Material 19. Netleague table for Anxiety in subgroup analysis (SMD, 95%CI)

| MA |  |  |  |  | -2.94  [ -9.54; 3.66] |  |  | -7.80  [-17.54; 1.94] | **-15.04**  **[-21.54; -8.54]** |
| --- | --- | --- | --- | --- | --- | --- | --- | --- | --- |
| -1.41  [-13.51; 10.70] | AA + Usual care |  |  |  |  | -4.87  [-14.10; 4.36] |  |  |  |
| -2.55  [-10.11; 5.01] | -1.15  [-13.93; 11.64] | TEAS |  |  |  |  |  | **-7.60**  **[-12.96; -2.24]** | -5.31  [-19.97; 9.35] |
| -4.18  [-17.02; 8.67] | -2.77  [-16.52; 10.98] | -1.62  [-15.11; 11.87] | MA + Usual care |  |  | -2.10  [-12.29; 8.09] |  |  |  |
| -4.89  [-11.32; 1.54] | -3.48  [-14.19; 7.23] | -2.33  [-10.02; 5.35] | -0.71  [-12.26; 10.83] | EA | -4.58  [-14.14; 4.98] | -2.63  [ -9.09; 3.83] | -2.11  [-11.27; 7.05] | -0.19  [ -9.46; 9.08] | -4.44  [-10.99; 2.12] |
| -5.05  [-10.86; 0.77] | -3.64  [-16.16; 8.87] | -2.50  [-11.34; 6.35] | -0.87  [-14.11; 12.36] | -0.16  [ -7.12; 6.80] | WM |  |  |  |  |
| -6.28  [-14.10; 1.55] | -4.87  [-14.10; 4.36] | -3.72  [-12.57; 5.12] | -2.10  [-12.29; 8.09] | -1.39  [ -6.82; 4.05] | -1.23  [ -9.68; 7.22] | Usual care | -1.93  [ -8.99; 5.12] |  | **-6.93**  **[-13.43; -0.43]** |
| -6.72  [-14.01; 0.56] | -5.32  [-16.17; 5.53] | -4.17  [-12.49; 4.14] | -2.55  [-14.22; 9.12] | -1.84  [ -7.57; 3.90] | -1.68  [ -9.86; 6.51] | -0.45  [ -6.14; 5.24] | AA |  | -1.43  [ -6.75; 3.88] |
| **-9.57**  **[-15.48; -3.67]** | -8.17  [-20.07; 3.74] | **-7.02**  **[-12.08; -1.96]** | -5.40  [-18.06; 7.26] | -4.69  [-10.77; 1.40] | -4.53  [-12.01; 2.96] | -3.30  [-10.81; 4.22] | -2.85  [ -9.76; 4.06] | Placebo |  |
| **-12.21**  **[-17.37; -7.05]** | -10.81  [-22.17; 0.56] | **-9.66**  **[-16.08; -3.24]** | -8.04  [-20.19; 4.11] | **-7.32**  **[-12.50; -2.15]** | **-7.16**  **[-14.03; -0.30]** | -5.94  [-12.56; 0.69] | -5.49  [-11.17; 0.20] | -2.64  [ -7.16; 1.88] | No treatment |

*The black bold result means it had statistically difference between the interventions and controls. In the case of effect, when the standard mean difference is < 0, it means the effectiveness of the column treatment is better, otherwise, it not.

Supplementary Material 20. Sensitivity analysis for Anxiety in the subgroup analysis

| 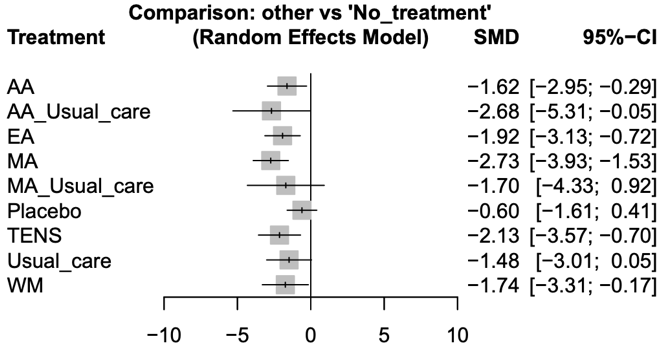  (Excluded Zhang 2000) | 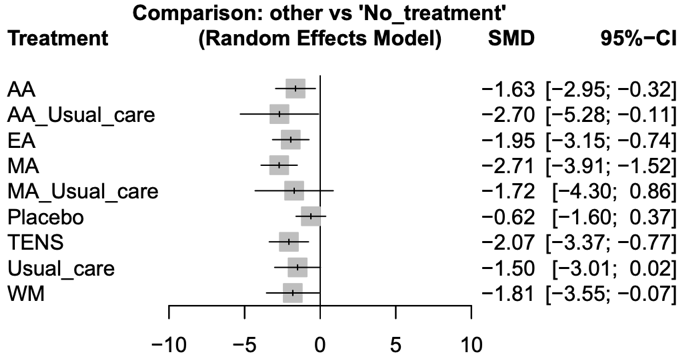  (Excluded Song 2002) |
| --- | --- |
| 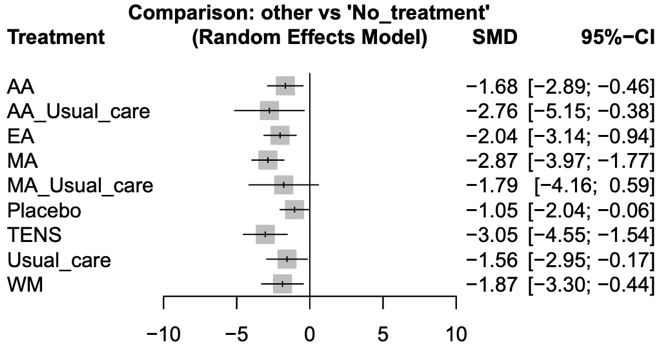  (Excluded Shu 2003) | 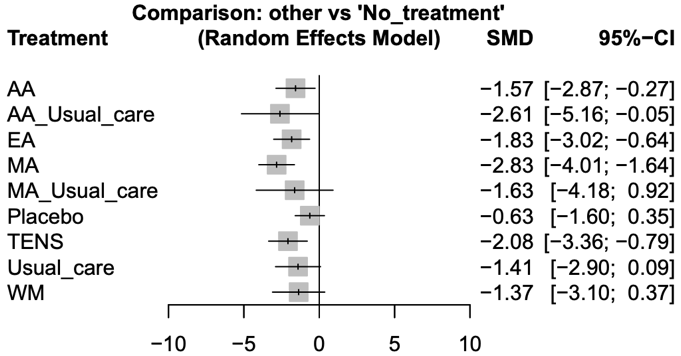  (Excluded Wen 2005) |
| 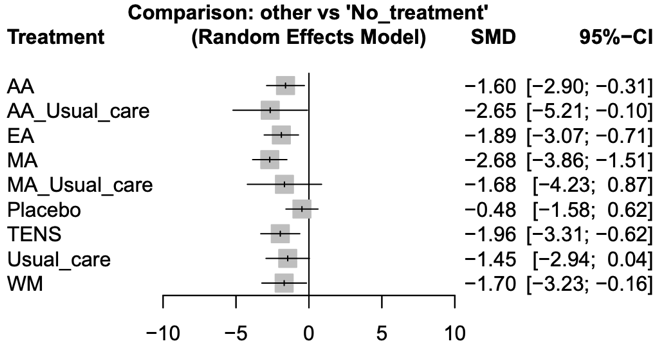  (Excluded Mu 2005) | 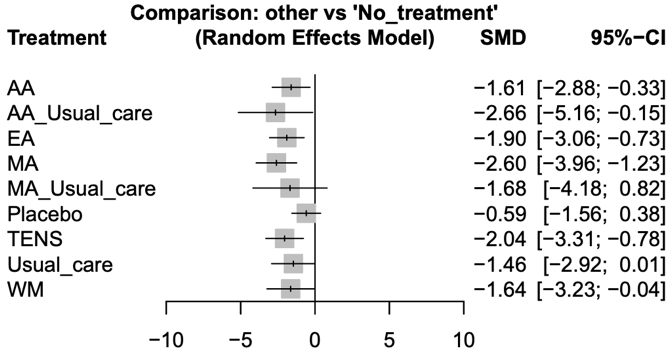  (Excluded Liang 2008) |
| 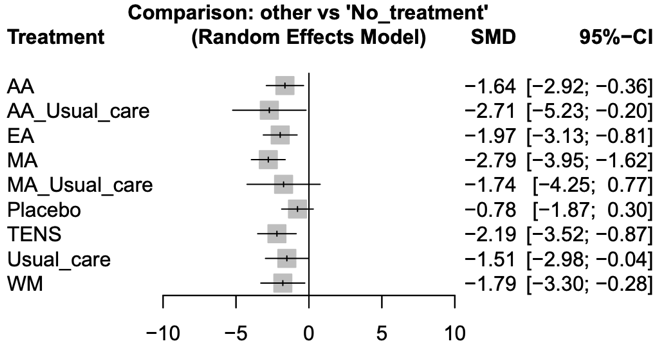  (Excluded Mu 2009) | 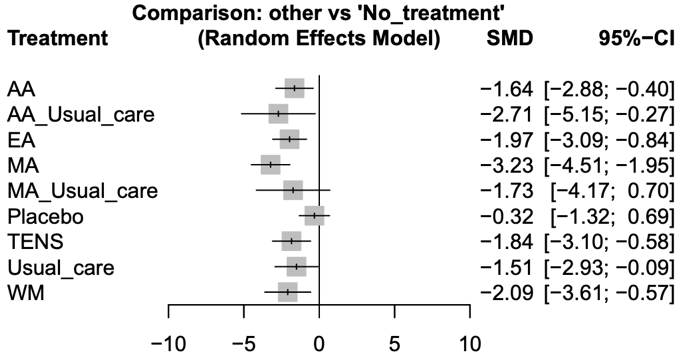  (Excluded Li 2012) |
| 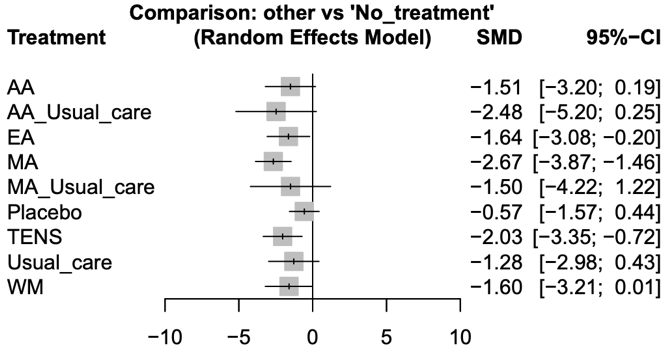  (Excluded Liang 2014) | 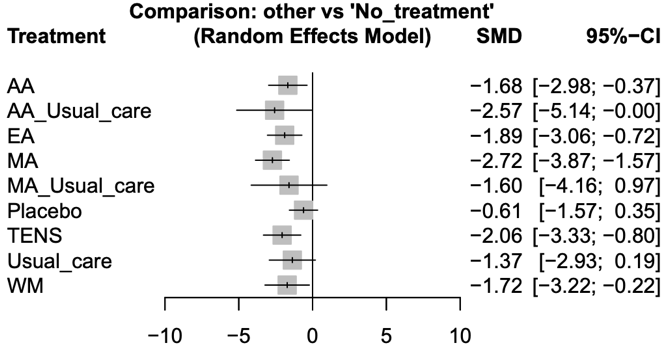  (Excluded Jackson 2016) |
| 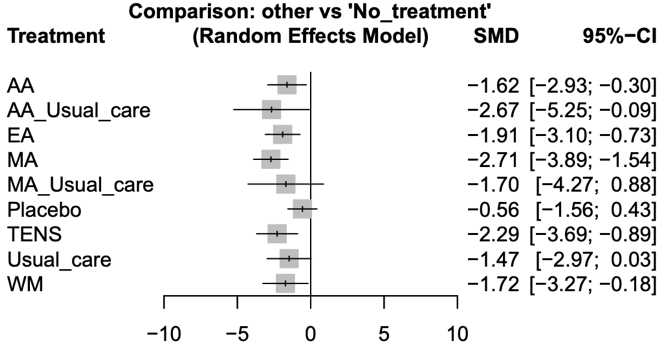  (Excluded Liao 2017) | 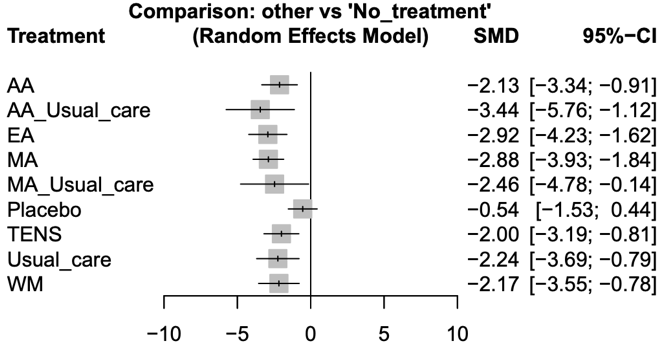  (Excluded Wang 2020) |
| 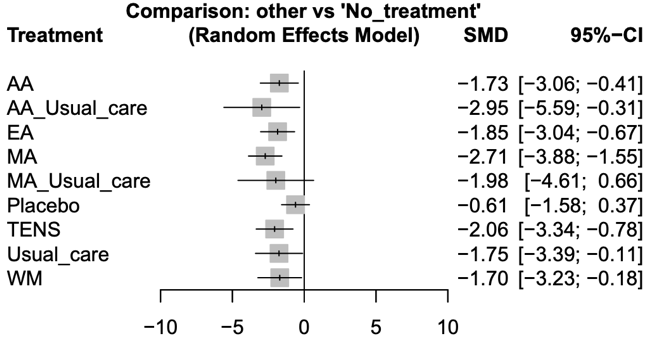  (Excluded Gu 2023) | 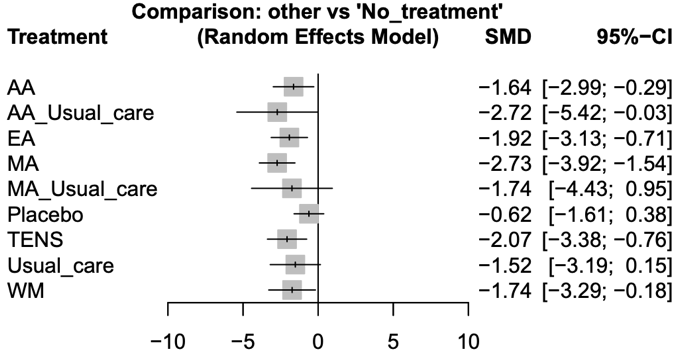  (Excluded Kong 2021) |
| (Excluded Mu 2013) | (Excluded Zhu 2008) |
| (Excluded Xu 2016) | (Excluded Li 2014) |
| (Excluded Ahlberg 2015) | (Excluded Chen 2015) |
| (Excluded Shen 2021) | (Excluded Jackson 2016 and Ahlberg 2015) |

Supplementary Material 21. Meta-regression of anxiety

Supplementary Material 22. Subgroup analysis based on the type of drug and treatment duration (Anxiety)

Supplementary Material 23. Funnel plot of withdrawal symptoms

Abbreviation: A: Western medicine, B: Warm acupuncture, C: Acupuncture therapies, D: Electronic stimulation, E: Usual care, F: No treatment, G: Acupuncture therapies + Western medicine, H: Electronic stimulation + Western medicine, I: Placebo, J: Acupuncture therapies +Usual care

Supplementary Material 24. Funnel plot of depression

Abbreviation: A: No treatment, B: Electronic stimulation, C: Acupuncture therapies, D: Usual care, E: Placebo, F: Acupuncture therapies +Usual care

Supplementary Material 25. Funnel plot of anxiety.

Abbreviation: A: Western medicine, B: Usual care, C: Electronic stimulation, D: Acupuncture therapies, E: No treatment, F: Placebo, G: Acupuncture therapies +Usual care

Supplementary Material 26. Quality of evidence

| Withdraw symptoms | | | | |
| --- | --- | --- | --- | --- |
| Comparison | | Direct evidence | Indirect evidence | Network meta-analysis |
| Acupuncture therapies + Usual care | Electronic stimulation | - | Very Low | Very Low |
| Acupuncture therapies + Usual care | Acupuncture therapies | - | Very Low | Very Low |
| Acupuncture therapies + Usual care | Warm acupuncture | - | Very Low | Very Low |
| Acupuncture therapies + Usual care | Acupuncture therapies + WM | - | Very Low | Very Low |
| Acupuncture therapies + Usual care | Electronic stimulation + WM | - | Very Low | Very Low |
| Acupuncture therapies + Usual care | WM | - | Very Low | Very Low |
| Acupuncture therapies + Usual care | Placebo | - | Moderate | Very Low |
| Acupuncture therapies + Usual care | Usual care | Very Low | Very Low | Very Low |
| Acupuncture therapies + Usual care | No treatment | - | Very Low | Very Low |
| Electronic stimulation | Acupuncture therapies | - | Very Low | Very Low |
| Electronic stimulation | Warm acupuncture | - | Very Low | Very Low |
| Electronic stimulation | Acupuncture therapies + WM | - | Very Low | Very Low |
| Electronic stimulation | Electronic stimulation + WM | Very Low | Very Low | Very Low |
| Electronic stimulation | WM | - | Very Low | Very Low |
| Electronic stimulation | Placebo | Moderate | Moderate | Moderate |
| Electronic stimulation | Usual care | Very Low | Very Low | Very Low |
| Electronic stimulation | No treatment | Very Low | Very Low | Very Low |
| Acupuncture therapies | Warm acupuncture | - | Very Low | Very Low |
| Acupuncture therapies | Electronic stimulation + WM | Very Low | Very Low | Very Low |
| Acupuncture therapies | WM | Very Low | Very Low | Very Low |
| Acupuncture therapies | Placebo | Moderate | Moderate | Moderate |
| Acupuncture therapies | Usual care | Very Low | Very Low | Very Low |
| Acupuncture therapies | No treatment | Very Low | Very Low | Very Low |
| Warm acupuncture | Acupuncture therapies + WM | - | Very Low | Very Low |
| Warm acupuncture | Electronic stimulation + WM | - | Very Low | Very Low |
| Warm acupuncture | WM | - | Very Low | Very Low |
| Warm acupuncture | Placebo | - | Moderate | Moderate |
| Warm acupuncture | Usual care | Very Low | Very Low | Very Low |
| Warm acupuncture | No treatment | - | Very Low | Very Low |
| Acupuncture therapies + WM | Electronic stimulation + WM | Very Low | Very Low | Very Low |
| Acupuncture therapies + WM | WM | Very Low | Very Low | Very Low |
| Acupuncture therapies + WM | Placebo | - | Moderate | Moderate |
| Acupuncture therapies + WM | Usual care | - | Very Low | Very Low |
| Acupuncture therapies + WM | No treatment | - | Very Low | Very Low |
| Electronic stimulation + WM | WM | Very Low | Very Low | Very Low |
| Electronic stimulation + WM | Placebo | - | Moderate | Moderate |
| Electronic stimulation + WM | Usual care | - | Very Low | Very Low |
| Electronic stimulation + WM | No treatment | - | Very Low | Very Low |
| WM | Placebo | - | Moderate | Moderate |
| WM | Usual care | - | Very Low | Very Low |
| WM | No treatment | - | Very Low | Very Low |
| Placebo | Usual care | - | Very Low | Very Low |
| Placebo | No treatment | Moderate | Moderate | Moderate |
| Usual care | No treatment | - | Very Low | Very Low |
| Withdraw symptoms (in subgroup analysis) | | | | |
| TEAS | MA | - | Very Low | Very Low |
| TEAS | AA+ Usual care | - | Very Low | Very Low |
| TEAS | MA+WM | - | Very Low | Very Low |
| TEAS | EA+WM | - | Very Low | Very Low |
| TEAS | EA | - | Very Low | Very Low |
| TEAS | TEAS+WM | - | Very Low | Very Low |
| TEAS | WM | - | Very Low | Very Low |
| TEAS | WA | - | Very Low | Very Low |
| TEAS | AA | - | Very Low | Very Low |
| TEAS | Placebo | Moderate | Moderate | Moderate |
| TEAS | Usual care | - | Very Low | Very Low |
| TEAS | No treatment | Very Low | Very Low | Very Low |
| MA | AA+ Usual care | - | Very Low | Very Low |
| MA | MA+WM | - | Very Low | Very Low |
| MA | EA+WM | Very Low | Very Low | Very Low |
| MA | EA | - | Very Low | Very Low |
| MA | TEAS+WM | Very Low | Very Low | Very Low |
| MA | WM | Very Low | Very Low | Very Low |
| MA | WA | - | Very Low | Very Low |
| MA | AA | - | Very Low | Very Low |
| MA | Placebo | Moderate | Moderate | Moderate |
| MA | Usual care | - | Very Low | Very Low |
| MA | No treatment | Very Low | Very Low | Very Low |
| AA+ Usual care | MA+WM | - | Very Low | Very Low |
| AA+ Usual care | EA+WM | - | Very Low | Very Low |
| AA+ Usual care | EA | - | Very Low | Very Low |
| AA+ Usual care | TEAS+WM | - | Very Low | Very Low |
| AA+ Usual care | WM | - | Very Low | Very Low |
| AA+ Usual care | WA | - | Very Low | Very Low |
| AA+ Usual care | AA | - | Very Low | Very Low |
| AA+ Usual care | Placebo | - | Very Low | Very Low |
| AA+ Usual care | Usual care | Very Low | Very Low | Very Low |
| AA+ Usual care | No treatment | Very Low | Very Low | Very Low |
| MA+WM | EA+WM | - | Very Low | Very Low |
| MA+WM | EA | - | Very Low | Very Low |
| MA+WM | TEAS+WM | Very Low | Very Low | Very Low |
| MA+WM | WM | Very Low | Very Low | Very Low |
| MA+WM | WA | - | Very Low | Very Low |
| MA+WM | AA | - | Very Low | Very Low |
| MA+WM | Placebo | - | Very Low | Very Low |
| MA+WM | Usual care | - | Very Low | Very Low |
| MA+WM | No treatment | - | Very Low | Very Low |
| EA+WM | EA | - | Very Low | Very Low |
| EA+WM | TEAS+WM | - | Very Low | Very Low |
| EA+WM | WM | Very Low | Very Low | Very Low |
| EA+WM | WA | - | Very Low | Very Low |
| EA+WM | AA | - | Very Low | Very Low |
| EA+WM | Placebo | - | Very Low | Very Low |
| EA+WM | Usual care | - | Very Low | Very Low |
| EA+WM | No treatment | Very Low | Very Low | Very Low |
| EA | TEAS+WM | - | Very Low | Very Low |
| EA | WM | Very Low | Very Low | Very Low |
| EA | WA | - | Very Low | Very Low |
| EA | AA | - | Very Low | Very Low |
| EA | Placebo | Moderate | Moderate | Moderate |
| EA | Usual care | Very Low | Very Low | Very Low |
| EA | No treatment | Very Low | Very Low | Very Low |
| TEAS+WM | WM | Very Low | Very Low | Very Low |
| TEAS+WM | WA | - | Very Low | Very Low |
| TEAS+WM | AA | - | Very Low | Very Low |
| TEAS+WM | Placebo | - | Very Low | Very Low |
| TEAS+WM | Usual care | - | Very Low | Very Low |
| TEAS+WM | No treatment | - | Very Low | Very Low |
| WM | WA | - | Very Low | Very Low |
| WM | AA | - | Very Low | Very Low |
| WM | Placebo | - | Very Low | Very Low |
| WM | Usual care | - | Very Low | Very Low |
| WM | No treatment | - | Very Low | Very Low |
| WA | AA | - | Very Low | Very Low |
| WA | Placebo | - | Very Low | Very Low |
| WA | Usual care | Very Low | Very Low | Very Low |
| WA | No treatment | - | Very Low | Very Low |
| AA | Placebo | Moderate | Moderate | Moderate |
| AA | Usual care | Very Low | Very Low | Very Low |
| AA | No treatment | Very Low | Very Low | Very Low |
| Placebo | Usual care | - | Very Low | Very Low |
| Placebo | No treatment | Moderate | Moderate | Moderate |
| Usual care | No treatment | - | Very Low | Very Low |

Abbreviation: AA: auricular acupuncture, MA: Manual acupuncture, EA: electro acupuncture, TEAS: transcutaneous electric acupoint stimulation, WM: Western medicine, WA: Warm acupuncture
